# Supplementary material for: Whole-genome sequencing reveals a possible molecular basis of sex determination in the dioecious wild yam Dioscorea tokoro
Source: PLoS Genet. 2026 Apr 20;22(4):e1012123. doi: 10.1371/journal.pgen.1012123 (PMC13128126; doi:10.1371/journal.pgen.1012123)
Supplement: S1 Text — 1. Materials. 2. Reference assembly. 3. Generation of chromosomes using pseudo-testcross methods. 4. Identification of sex-linked regions by association analysis and mapping coverage analysis. 5. Genome structures of the X and Y chromosomes. 6. Identification of candidate genes for sex determination. 7. Identification of candidate miRNAs for sex determination. 8. Overexpression of BLH9 and AtBLH9 in Arabidopsis thaliana. (DOCX) [file pgen.1012123.s001.docx]

**S1 Text. Supplementary materials and methods**

Table of contents

1. Materials

1.1 Plant materials

1.2 Whole-genome sequencing of female and male individuals using Oxford Nanopore Technology

1.3 Illumina library construction and sequencing of female and male individuals

1.4 RAD-seq for linkage mapping and association analysis

1.5 RNA library construction and sequencing

1.6 Small RNA library construction and sequencing

2. Reference assembly

2.1 Estimation of genome size

2.2 Quality control

2.3 *De novo* assembly

2.4 Polishing and removal of duplicated contigs

2.5 TE annotation

2.6 Transcriptome-based gene identification

2.7 *Ab initio* gene prediction

2.8 Gene annotation

3. Generation of chromosomes using pseudo-testcross methods

3.1 Identification of parental line­–specific heterozygous markers

3.2 Linkage mapping

3.3 Integration of two parental-specific linkage maps into the chromosome-scale physical map

4. Identification of sex-linked regions by association analysis and mapping coverage analysis

4.1 Association analysis

4.2 Mapping coverage analysis

4.3 Confirmation of the assembly continuity around the X- and Y- specific regions

4.4 SDRs confirmation by SDpop and RADSex

5. Genome structures of the X and Y chromosomes

5.1 Gene density and repetitive sequence accumulation in X- and Y-specific regions

5.2 Estimation of divergence between X- and Y-linked gametologs

5.3 Comparison of sex chromosomes in the genus *Dioscorea*

6. Identification of candidate genes for sex determination

6.1 Identification of highly expressed genes in male flowers during early stages of development

6.2 Identification of candidate genes for sex determination

6.3 PCR amplification of the candidate genes

6.4 Phylogenetic analysis of candidate genes and their homologous genes

7. Identification of candidate miRNAs for sex determination

7.1 Sequence processing

7.2 miRNA prediction and annotation

7.3 Identification of highly expressed miRNAs in male flowers

7.4 Identification of candidate miRNAs for sex determination

8. Overexpression of *BLH9* and *AtBLH9* in *Arabidopsis thaliana*

8.1 Phylogenetic analysis of BLH9 and TALE superfamily proteins

8.2 Plant materials

8.3 Cloning and plasmid construction

8.4 Plant transformation

8.5 RT-qPCR

8.6 Measurement of inflorescence phenotypes

**1. Materials**

**1.1 Plant materials**

To construct the reference genome, a female *D. tokoro* Waka1 (original code: DT49) plant was collected from Tahara, Wakayama Pref., Japan. A male *D. tokoro* Kita1 (original code: 110628-5) plant was collected from Waga-Sennin, Kitakami, Iwate Pref., Japan. The 186 F_1_ progeny, comprising 38 females, 89 males, and 59 non-flowering individuals, were derived from a cross between female parent Waka1 and male parent Kita1. F_1_ seeds were obtained from the cross in 2011, and 206 F_1_ individuals were planted in 2012 at Iwate Biotechnology Research Center. The sex phenotypes of the 186 F_1_ individuals were obtained in 2014 and 2015. To analyze sex-linked regions, female and male individuals were collected from three wild populations: one each in northern Japan (KTKM; Kitakami, Iwate Pref., Japan), central Japan (SHG; Koka, Shiga Pref., Japan), and southern Japan (FKOK; Kasuya, Fukuoka Pref., Japan). To confirm sex-linked regions, five females and five males were collected from a wild population (KZGW; Kozagawa, Wakayama Pref., Japan). To conduct transcriptome analysis, 18 tissue samples were collected for RNA-seq. Male and female flowers were collected from wild populations in Takizawa and Kitakami, Iwate Prefecture, Japan, and non-reproductive organs were collected from Kita1. Fifteen samples were collected for small RNA-seq from the wild population in Koka, Shiga Pref., Japan. To check the male specificity of the candidate genes, five females and five males were collected from two wild populations: one in northern Japan (HNMK; Hanamaki, Iwate Pref., Japan) and one in Southern Japan (KMMT; Kumamoto, Kumamoto Pref., Japan). The latitudes and longitudes of the sampling sites are listed in Table A.

**1.2 Whole-genome sequencing of female and male individuals using Oxford Nanopore Technology**

Genomic DNA was extracted from fresh leaves of Waka1 (female) and Kita1 (male) plants using NucleoBond HMW DNA (Macherey-Nagel, Düren, Germany). The DNA was subjected to size selection and purification with Short Read Eliminator XL (Circulomics, Baltimore, MD, USA). Libraries were constructed using a Ligation Sequencing Kit SQK-LSK114. The libraries were sequenced on R10.4.1 flow cells (FLO-PRO114M) with the PromethION 2 Solo device at Iwate Biological Research Center. The raw sequencing data were subjected to base calling using dorado v0.8.1 with the dna_r10.4.1_e8.2_400bps_sup@v5.0.0 model and the option: “--min-qscore 8 --emit-fastq -c 10000 -r”.

**1.3 Illumina library construction and sequencing of female and male individuals**

Genomic DNA was extracted from Waka1 (female) and Kita1 (male) using a NucleoSpin Plant II Kit (Macherey-Nagel). Libraries for Waka1 (female) were constructed using a Collibri ES DNA Library Prep Kit for Illumina Systems (Invitrogen, Camarillo, CA, USA) and a TruSeq DNA PCR-Free LT Library Prep Kit (Illumina, San Diego, CA, USA). Sequencing libraries for Kita1 (male) were constructed using a TruSeq DNA PCR-Free LT Library Prep Kit. The libraries were sequenced via MiSeq and HiSeqX. Genomic DNA was extracted from female and male individuals from northern (KTKM), central (SHG), and southern (FKOK) Japan using a DNeasy Plant Maxi Kit (Qiagen, Hilden, Germany) following the manufacturer’s protocol. Contaminating proteins in the extracted DNA lysate were removed by phenol/chloroform extraction. The DNA was purified by ethanol precipitation. Sequencing libraries were constructed using a Collibri ES DNA Library Prep Kit for Illumina Systems with a fragment length of ~350 bp. The quality and quantity of the sequencing libraries were assessed using a Qubit fluorometer (Invitrogen), an Agilent Bioanalyzer with Agilent High Sensitivity DNA Kit (Agilent Technologies, Waldbronn, Germany), and a qPCR with Library Quantification Kit (Takara Bio, Mountain View, CA, USA). The libraries were sequenced on the HiSeqX platform (150 bp paired-end reads) by Rhelixa, Tokyo, Japan. Genomic DNA was extracted from five females and five males from KZGW population using Maxwell RSC Plant DNA Kit (Promega, Madison, WI, USA) following the manufacturer’s protocol. Sequencing libraries were constructed using a Collibri PCR-free ES DNA Library Prep Kit for Illumina Systems with a fragment length of ~350 bp and sequenced using the Novaseq X Plus system (150 bp paired-end reads) by Nippon Genetics, Tokyo, Japan (Table B).

**1.4 RAD-seq for linkage mapping and association analysis**

RAD-seq was performed as previously described [1]. Genomic DNA was extracted from fresh leaves of Waka1, Kita1, and 186 F_1_ individuals using a NucleoSpin Plant II Kit (Macherey-Nagel). The DNA was digested with the restriction enzymes PacI and NlaIII, and the libraries for 75-bp paired-end reads were sequenced on the Illumina NextSeq 500 platform. Adapters and unpaired reads were removed using FaQCs and PRINSEQ lite. The filtered RAD-seq reads were used to construct linkage maps and for association analysis (Data S1).

**1.5 RNA library construction and sequencing**

RNA-seq data were obtained from 18 samples, including male and female flowers and non-reproductive organs of *D. tokoro.* The samples included male and female flowers at five stages of development: inflorescence stage 0, inflorescence stage 1, inflorescence stage 2, buds, and flowers. The samples also included eight non-reproductive organs: vegetative shoot apex, leaves, stems, root apex, rhizome root, rhizome stem, rhizome bud, and rhizome storage tissues. Total RNA was extracted from the samples using an RNeasy Plant Mini Kit (Qiagen) as described previously [1] with slight modifications. The cDNA library was constructed using a TruSeq RNA Sample Prep Kit V2 (Illumina) and sequenced on the Illumina NextSeq 500 platform (Table C).

**1.6 Small RNA library construction and sequencing**

Small RNA-seq data were obtained from 15 samples, including male and female flowers and non-reproductive organs of *D. tokoro.* The samples included male and female flowers at five stages of development: inflorescence stage 0, inflorescence stage 1, inflorescence stage 2, buds, and flowers. The samples also included three non-reproductive organs: the vegetative shoot apex, male and female leaves, and male and female stems. Total RNA was extracted from the samples using an Ambion Plant RNA Isolation Aid (Ambion, Austin, TX, USA) following the manufacturer’s protocol with slight modifications. The frozen and powdered samples were mixed with 100 μL of Plant RNA Isolation Aid and 1 mL lysis solution

and thoroughly homogenized. The homogenized samples were centrifuged at 15,000 g in a microcentrifuge for 5 min at room temperature. The supernatants with total RNA were transferred to new tubes. The small RNA fraction was isolated from total RNA using a mirVana miRNA Isolation kit (Ambion) following the manufacturer’s protocol. The small RNA libraries were constructed using a NEBNext Multiplex Small RNA Library Prep Set for Illumina (New England BioLabs, Ipswich, MA, USA) following the manufacturer’s protocol from the adapter ligation step to the PCR amplification step. For quality control, the PCR-amplified cDNA constructs were purified using DNA Clean & Concentrator-5 (Zymo Research, CA, USA) following the manufacturer’s protocol. After eluting the purified DNA, size selection using AMPure XP Beads was conducted following the NEBNext Multiplex Small RNA Library Prep Set for Illumina (New England BioLabs) protocol. The quality and quantity of the libraries were assessed using a Qubit fluorometer (Invitrogen), Agilent BioAnalyzer with Agilent High Sensitivity DNA Kit (Agilent Technologies), and qPCR with Library Quantification Kit (Takara Bio). The libraries were sequenced on the NovaSeq 6000 platform at Genebay, Yokohama, Japan (Table D).

**2. Reference assembly**

**2.1 Estimation of genome size**

The genome size of *D. tokoro* individual Kita1 was estimated by flow cytometry using nuclei prepared from fresh leaf samples. *D. rotundata* accession TDr96-F1 (570 Mb) [1] was used as an internal reference. DNA from isolated nuclei was stained with propidium iodide and analyzed using a Cell Lab Quanta SC Flow Cytometer (Beckman Coulter, USA) following the manufacturer’s protocol. The G1 peak mean value of *D. tokoro* was 206.5, and that of *D. rotundata* was 303.6. Based on the ratio between the two species of 0.68 (206.5/303.6), the genome size of *D. tokoro* was estimated to be ∼388 Mb (570 Mb × 0.68) (S2 Fig).

**2.2 Quality control**

Whole-genome assembly of female and male individuals was conducted using long reads generated by Oxford Nanopore Technology. The long-read data for Waka1 (female) and Kita1 (male) were obtained as described in section 1.2 above. As the first step in the pipeline for the reference assembly, the raw sequencing data were filtered. For both of Waka1 (female) and Kita1 (male) reads, those with an average read quality score of <10 and length of <1,000 bases were removed with chopper v0.8.0 [2] (Table E).

**2.3 *De novo* assembly**

The filtered long reads of Waka1 (female) and Kita1 (male) were assembled using PECAT [3] with the options genome_size=388000000, prep_min_length=3000, prep_output_coverage=80, and corr_output_coverage=80. This assembly step generated two phased assemblies for Waka1 (female): 180 contigs with N50 of 19,154,656 bp and a total size of 425.8 Mb for haplotype 1 and 326 contigs with N50 of 3,042,110 bp and a total size of 342.7 Mb for haplotype 2. This assembly step also generated two phased assemblies for Kita1 (male): 128 contigs with N50 of 33,851,599 bp and a total size of 415.0 Mb for haplotype 1 and 415 contigs with N50 of 1,379,312 bp and a total size of 300.8 Mb for haplotype 2.

**2.4 Polishing and removal of duplicated contigs**

The four assembled contig sets were polished (Fig A). To correct the assembled contigs, a consensus module was generated using Racon v1.5.0 [4]. The assembled contigs were polished and corrected using Medaka v1.7.2 (Oxford Nanopore Technologies, 2018) with the option “-m r1041_e82_400bps_sup_g615”. Finally, the scaffolds were polished twice using each Illumina short read from Waka1 (female) and Kita1 (male) with Hypo v1.0.3 [5]. For quality control of the Illumina short reads, adapters, reads of <50 bp and low-quality reads with an average quality score < 20 were removed using FaQCs v2.08 [6]. In the Hypo step, the coverage was set to 63 for Waka1 (female) haplotype 1 contigs and 71 for Waka1 (female) haplotype 2 contigs based on a calculation by CoverM v0.6.1 [7]. The coverage was set to 146 for Kita1 (male) haplotype 1 contigs and 181 for Kita1 (male) haplotype 2 contigs. The genome size was set to 443 Mb based on a previous assembly available at the DNA Databank of Japan database under BioProject PRJDB12945 because the newly constructed male primary scaffolds and female scaffolds were larger than 388 Mb, estimated as described in section 2.1. To evaluate the completeness of the gene set in each step, BUSCO (Bench-Marking Universal Single Copy) v5.2.2 and v5.5.0 [8] was utilized with “genome” as the assessment mode and Embryophyta odb10 as the database (Table F).

**
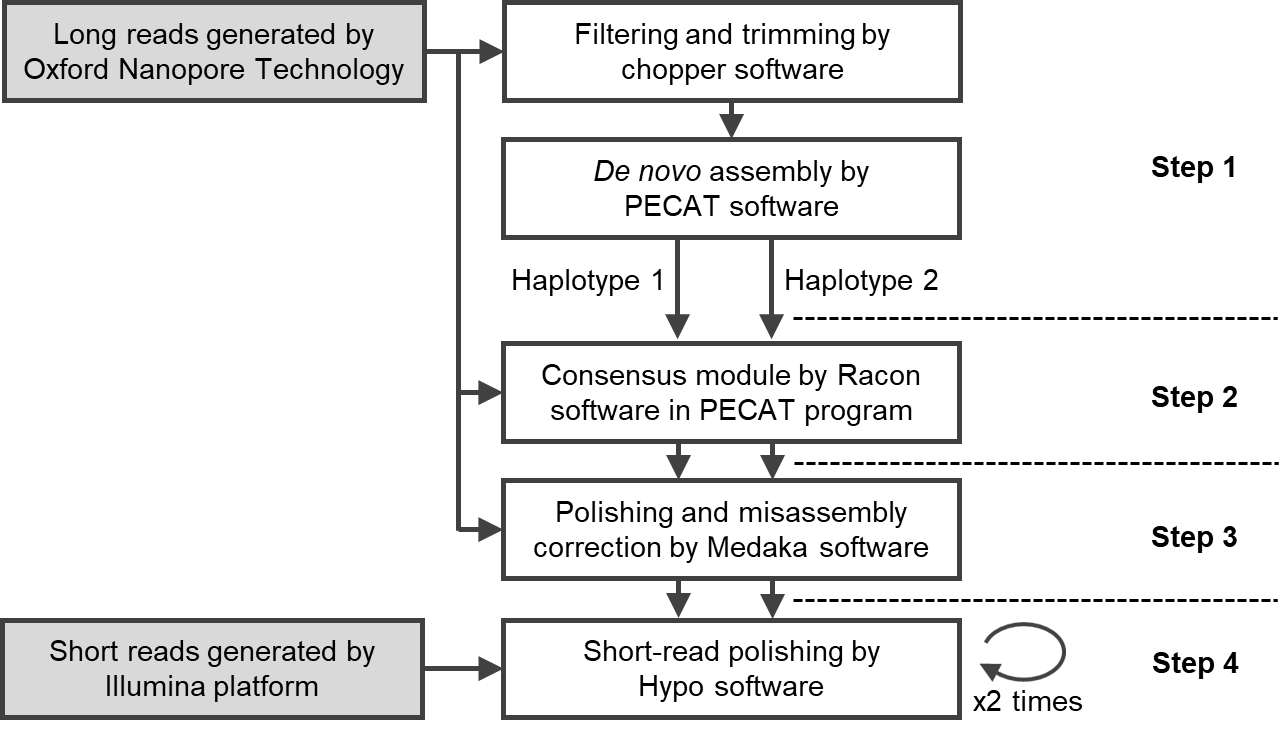
**

**Fig A.** **Pipeline for genome assembly**

**2.5 TE annotation**

To annotate repetitive sequences, *de novo* repeat libraries were constructed for each assembly using EDTA (The Extensive *de novo* TE Annotator) v2.1.0 [9] with the options --anno 1, --species others, and --step all. Using the resulting files, each genome FASTA file was soft-masked with the perl script make_masked.pl provided in EDTA with the options -maxdiv 30 -minscore 1000 -minlen 1000 -misschar N -hardmask 0.

**2.6** **Transcriptome-based gene identification**

For transcriptome-based gene identification using RNA-seq data from 18 samples of *D. tokoro*, poly(A) sequences of <50 bp were removed from raw RNA-seq reads with FaQCs v2.10 [6]. Low-quality bases on read ends with an average quality score of <20 were trimmed using PRINSEQ lite v0.20.4 [10] using a window size of 5. Low-quality reads with an average read quality score of <20 were also removed using PRINSEQ lite v0.20.4.

The filtered RNA-seq reads were aligned to the assembled contigs with HISAT2 v2.2.1 [11] with the option “--max-intronlen 15000 --dta”. The aligned transcriptomes were assembled using StringTie v3.0.0 [12], and open reading frame regions were identified using TransDecoder v5.5.0 [13]. Gene models were excluded from further analysis if their coding sequences (CDSs) contained an internal stop codon, if the CDS lengths were not multiples of three, or if they encoded proteins of <50 amino acids.

**2.7 *Ab initio* gene prediction**

BRAKER2 v2.1.6 [14] was used for *ab initio* gene prediction using the filtered RNA-seq data from *D. tokoro* and protein homology information from *D. alata* and *D. rotundata* with the options --addUTR=on, --softmasking, and --etpmode. Protein sequences from *D. alata* TDa95/00328 and *D. rotundata* TDr96_F1 were downloaded from NCBI (accession numbers GCA_020875875.1 and GCF_009730915.1, respectively). The predicted genes were categorized into three groups—gene models fully supported by protein hints, gene models at least partially supported by protein hints, and gene models without any support—using the Python script selectSupportedSubsets.py in BRAKER. Gene models fully supported by protein hints were selected, and gene models whose CDS contained an internal stop codon, gene models whose the CDS lengths were not multiples of three, and gene models whose CDS encoded proteins of <50 amino acids were removed.

**2.8 Gene annotation**

Finally, all predicted genes were merged using GffCompare v0.12.6 [15]. *Ab initio* predicted genes were selected with no or less overlap with genes predicted by StringTie using overlapping class codes: u (no overlap), i (fully contained within a reference intron), and y (contained a reference within its introns). The selected *ab initio* predicted genes were combined with gene annotations generated by Stringtie. Gene predictions obtained from GeneMark in BRAKER2 were excluded. The final annotations include 21,308 genes in the Waka1 (female) haplotype 1 assembly, 17,710 genes in the Waka1 (female) haplotype 2 assembly, 23,200 genes in the Kita1 (male) haplotype 1 assembly, and 18,145 genes in the Kita1 (male) haplotype 2 assembly. To evaluate the completeness of the gene sets in the final scaffolds, BUSCO (Bench-Marking Universal Single Copy) v5.5.5 [8] was utilized with “transcriptome” as the assessment mode and Embryophyta odb10 as the database (S2 Table).

**3. Generation of chromosomes using pseudo-testcross methods**

**3.1 Identification of parental line–specific heterozygous markers**

Chromosome-scale genome sequences from the assembled contigs were developed using SNP-type heterozygous markers and presence/absence-type heterozygous markers from RAD-seq data from Waka1, Kita1, and 186 F_1_ individuals. Parental line–specific heterozygous markers were identified as described previously [16] with the following modifications.

***SNP-type heterozygous markers***

To obtain SNP-type heterozygous markers, the RAD-seq data were aligned to each reference genome using BWA v0.7.18-r1243-dirty [17]. The alignments with high mapping quality (≥60) and properly paired were selected using the Samtools view command. Based on these alignments, SNP-based genotypes were obtained using Bcftools v1.15 [18] with the following commands: (i) mpileup command with the option “-t DP,AD -B -Q 13 -C 50”; (ii) call command with the option “-P 0 -v -m -a GQ,GP”; (iii) filter command with the options “-i ‘INFO/MQ≥40, INFO/MQ0F≤0.1, and AVG(GQ)≥10”; and (iv) norm command with the option “-m+any.” Biallelic SNPs were selected based on the ALT field of VCF. In addition, variants with low read depth (<9) or low genotype quality scores (<14) in the two parents were removed. Variants with low read depth (<5) or low genotype quality scores (<14) in F_1_ progeny considered to be missing were also removed, and only variants with low missing rates (<0.1) were retained. After obtaining SNP-based genotypes, a binomial test was performed to reject SNPs affected by segregating distortion in the F_1_ progeny. This binomial test assumes that the probability of success is 0.5 based on the two-side hypothesis, and variants with *p*-values < 0.01 were regarded as having segregation distortion. The following SNP-type heterozygous markers were ultimately obtained: 1,227 female-parent-heterozygous SNP markers and 1,219 male-parent-heterozygous SNP markers for the Waka1 haplotype 1 (female) reference; 1,259 female-parent-heterozygous SNP markers and 919 male-parent-heterozygous SNP markers for the Waka1 (female) haplotype 2 reference; 1,618 female-parent-heterozygous SNP markers and 964 male-parent-heterozygous SNP markers for the Kita1 (male) haplotype 1 reference; and 1,108 female-parent-heterozygous SNP markers and 985 male-parent-heterozygous SNP markers for the Kita1 (male) haplotype 2 reference.

***Presence/absence-type heterozygous markers***

To obtain presence/absence-type heterozygous markers, the RAD-seq data were aligned to each reference genome using BWA v0.7.18-r1243-dirty [17]. The alignments with high mapping quality (≥60) and properly paired were selected using the Samtools view command. Based on the alignment read depth of the two parental plants, Waka1 (female) and Kita1 (male), presence/absence-based genotypes were obtained using Bcftools v1.15 [18] with the following commands: (i) mpileup command with the option “DP,AD”; (ii) call command with the option “-P 0 -A -m -f GQ,GP”; and (iii) view command with the options “-i ‘MAX(FMT/DP)≥4 & MIN(FMT/DP) ≤0’ -g miss -V indels”. These presence/absence-based markers included genotypes in which one parent (Waka1 or Kita1) has sufficient read depth (≥4) and the other parent has no read depth. After obtaining the genotypes in VCF format, continuous positions in VCF format were converted to a feature that provides a region’s start and end coordinate information using the BEDtools v2.31.1 [19] merge command with the option “-d 10 -c 1 -o count”. Only wide features (≥50 bp) were retained in the BED file. The total read base count (i.e., the sum of per-base read depth) was obtained using the Samtools bedcov command. Based on the depth value, presence/absence-based genotypes were determined. For Waka1 (female) and Kita1 (male), genotypes with depth ≥ 4 were regarded as present genotypes, meaning heterozygosity of presence and absence, and genotypes with depth = 0 were regarded as absent genotypes, meaning homozygosity of absence. For F_1_ progeny, markers with depth ≥ 2 and depth = 0 were classified as present and absent markers, respectively. After obtaining presence/absence-based genotypes, a binomial test assumes that the probability of success is 0.5 based on the two-side hypothesis, and variants with *p* < 0.01 were regarded as having segregation distortion. As a result, 479 female-parent-heterozygous presence/absence markers and 170 male-parent-heterozygous presence/absence markers were obtained for the Waka1 (female) haplotype 1 reference, 426 female-parent-heterozygous presence/absence markers and 126 male-parent-heterozygous presence/absence markers for the Waka1 (female) haplotype 2 reference, 236 female-parent-heterozygous presence/absence markers and 404 male-parent-heterozygous presence/absence markers for the Kita1 haplotype 1 (male) reference, and 161 female-parent-heterozygous presence/absence markers and 309 male-parent-heterozygous presence/absence markers for the Kita1 (male) haplotype 2 reference.

**3.2 Linkage mapping**

Linkage maps were constructed based on the SNP-type heterozygous markers and presence/absence-type heterozygous markers. For each marker set (female-parent-heterozygous and male-parent-heterozygous marker sets), the markers were converted into genotype-formatted data for linkage map construction using MSTmap v1.0 [20] with the following parameters: “populationtype DH; distancefunction kosambi; cutoffpvalue 0.000000000001; nomapdist 15.0; nomapsize 0; missingthreshold 25.0; estimationbeforeclustering no; detectbaddata no; objective_function ML”. After performing MSTmap, complemented-phased duplex linkage groups were generated by coupling- and repulsion-type markers under the pseudo-testcross approach. In this step, reversed marker datasets were prepared by inverting the genotypes of all markers. We combined the original and reversed datasets and reconstructed the linkage maps using MSTmap. Finally, we pruned correlated flanking markers to remove redundant markers. In this step, markers locating on same contig and positioned at identical genetic distances (cM) on the linkage map were condensed by retaining only the first and last markers of each consecutive group. Orphan linkage groups were trimmed out. After all filtering steps, the final number of markers for linkage map construction were as follows: for the Waka1 (female) haplotype 1 reference, 1,097 SNP markers and 406 presence/absence markers were obtained as female-parent-heterozygous markers. 1,133 SNP markers and 158 presence/absence markers were obtained as male-parent-heterozygous markers. For the Waka1 (female) haplotype 2 reference, 1,161 SNP markers and 385 presence/absence markers were obtained as female-parent-heterozygous markers. 880 SNP markers and 121 presence/absence markers were obtained as male-parent-heterozygous markers. For the Kita1 (male) haplotype 1 reference, 1,483 SNP markers and 221 presence/absence markers were obtained as female-parent-heterozygous markers. 896 SNP markers and 328 presence/absence markers were obtained as male-parent-heterozygous markers. For the Kita1 (male) haplotype 2 reference, 1,057 SNP markers and 159 presence/absence markers were obtained as female-parent-heterozygous markers. 933 SNP markers and 283 presence/absence markers were obtained as male-parent-heterozygous markers. Final two parental-specific linkage maps were reconstructed and visualized by R/qtl [21] and Asmap [22].

**3.3 Integration of two parental-specific linkage maps into the chromosome-scale physical map**

Based on two parental-specific linkage maps, the contigs were anchored and linearly ordered as pseudochromosomes using ALLMAPS [23]. Gene order–based synteny of the three newly constructed reference genomes was detected using MCscan [24]. The orthologous regions were identified using the “jcvi.compara.catalog ortholog” function with the option “--cscore=.99” and the “jcvi.compara.synteny screen” function with at least 50 collinear gene blocks. The detected collinearity was visualized using the “jcvi.graphics.karyotype” function. The repeat libraries and gene annotations were lifted over from each assembly to the chromosome-scale genome sequence using the liftOver function in ALLMAPS. The telomere repeats were identified using quarTeT v1.2.5 [25] with the option TeloExplorer -c plant. The numbers of anchored contigs and telomere repeats are shown in Table G.

**4. Identification of sex-linked regions by association analysis and mapping coverage analysis**

**4.1 Association analysis**

To identify sex-linked regions, association analysis was performed using markers obtained by RAD-seq data from the F_1_ progeny. SNP-type markers and presence/absence-type markers were identified as genotype data as described in section 3.1 with liberal thresholds.

***SNP-type heterozygous markers for association analysis***

To obtain SNP-type heterozygous markers, the RAD-seq data were aligned to each reference genome using BWA v0.7.18-r1243-dirty [17]. The alignments with high mapping quality (≥60) and properly paired were selected using the Samtools view command. Based on these alignments, SNP-based genotypes were obtained using Bcftools v1.15 [18] with the following commands: (i) mpileup command with the option “-t DP,AD,SP -B -Q 18 -C 50”; (ii) call command with the option “-P 0 -v -m -a GQ,GP”; (iii) filter command with the options “-i ‘INFO/MQ≥40, INFO/MQ0F≤0.1, and AVG(GQ)≥10”; and (iv) norm command with the option “-m+any.” Biallelic SNPs were selected with Bcftools view commands with the option “-m 2 -M 2 -v snps”. In addition, variants with low read depth (<10) or low genotype quality scores (<10) in the two parents were removed. Variants with low read depth (<8) or low genotype quality scores (<5) in F_1_ progeny considered to be missing were also removed, and only variants with low missing rates (<0.3) were retained. After obtaining SNP-based genotypes, heterozygous genotypes with unbalanced allele frequency (out of 0.4–0.6 in F_1_ progeny) were filtered out. Finally, a binomial test was performed to reject SNPs affected by segregating distortion in the F_1_ progeny. This binomial test assumes that the probability of success is 0.5 based on the two-side hypothesis, and variants with *p*-values < 0.01 were regarded as having segregation distortion. The following SNP-type heterozygous markers were ultimately obtained: 4,185 female-parent-heterozygous SNP markers and 6,041 male-parent-heterozygous SNP markers for the Waka1 (female) haplotype 1 reference; 4,019 female-parent-heterozygous SNP markers and 4,798 male-parent-heterozygous SNP markers for the Waka1 (female) haplotype 2 reference; 6,707 female-parent-heterozygous SNP markers and 3,963 male-parent-heterozygous SNP markers for the Kita1 (male) haplotype 1 reference; and 4,779 female-parent-heterozygous SNP markers and 3,672 male-parent-heterozygous SNP markers for the Kita1 (male) haplotype 2 reference.

***Presence/absence-type heterozygous markers for association analysis***

To obtain presence/absence-type heterozygous markers, the RAD-seq data were aligned to each reference gnome using BWA v0.7.18-r1243-dirty [17]. The alignments with high mapping quality (≥60) and properly paired were selected using the Samtools view command. Based on the alignment read depth of the two parental plants, Waka1 (female) and Kita1 (male), presence/absence-based genotypes were obtained using Bcftools v1.15 [18] with the following commands: (i) mpileup command with the option “DP,AD,SP,ADF,ADR,INFO/ADF,INFO/ADR -B -Q 18 -C 50”; (ii) call command with the option “-P 0 -A -m -f GQ,GP”; and (iii) view command with the options “-i ‘MAX(FMT/DP)≥4 & MIN(FMT/DP) ≤0’ -g miss -V indels”. These presence/absence-based markers included genotypes in which one parent (Waka1 or Kita1) has sufficient read depth (≥4) and the other parent has no read depth. After obtaining the genotypes in VCF format, continuous positions in VCF format were converted to a feature that provides a region’s start and end coordinate information using the BEDtools v2.31.1 [19] merge command with the option “-d 10 -c 1 -o count”. Only wide features (≥50 bp) were retained in the BED file. The total read base count (i.e., the sum of per-base read depth) was obtained using the Samtools bedcov command. Based on the depth value, presence/absence-based genotypes were determined. For Waka1 (female) and Kita1 (male), genotypes with depth ≥ 4 were regarded as present genotypes, meaning heterozygosity of presence and absence, and genotypes with depth = 0 were regarded as absent genotypes, meaning homozygosity of absence. For F_1_ progeny, markers with depth ≥ 2 and depth = 0 were classified as present and absent markers, respectively. After obtaining presence/absence-based genotypes, heterozygous genotypes with unbalanced allele frequency (out of 0.4–0.6 in F_1_ progeny) were filtered out. Finally, a binomial test was performed to reject SNPs affected by segregating distortion in the F_1_ progeny. This binomial test assumes that the probability of success is 0.5 based on the two-side hypothesis, and variants with *p* < 0.01 were regarded as having segregation distortion. As a result, 4,483 female-parent-heterozygous presence/absence markers and 1,301 male-parent-heterozygous presence/absence markers were obtained for the Waka1 (female) haplotype 1 reference, 3,712 female-parent-heterozygous presence/absence markers and 1,008 male-parent-heterozygous presence/absence markers were obtained for the Waka1 (female) haplotype 2 reference, 2,391 female-parent-heterozygous presence/absence markers and 2,988 male-parent-heterozygous presence/absence markers for the Kita1 (male) haplotype 1 reference, and 1,719 female-parent-heterozygous presence/absence markers and 2,153 male-parent-heterozygous presence/absence markers for the Kita1 (male) haplotype 2 reference.

The associations between the genotypes and sex phenotypes of the 127 flowering F_1_ individuals were calculated using Fisher’s exact test. The *q*-value for the Fisher’s exact test was obtained for each marker by comparing the frequencies of particular alleles and sex phenotypes categorized as female or male. The log transformed *q*-values (–log_10_(*q*)) for each position were visualized as Manhattan plots (Figs 2B, C; S15, S16). The false discovery rate (FDR) was set to 0.05 and was corrected by Benjamini-Hochberg correction. Each adjusted threshold is shown in S15, S16 Figs. When there were no values for rejected hypotheses before FDR correction in the analysis, threshold = –log_10_(0.05) was used. Statistical analysis and visualization were performed using an in-house Python script. In addition, the associations between the genotypes and sex phenotypes were calculated using Fisher’s exact test with equal numbers of randomly selected female and male F_1_ individuals (38 females and 38 males).

The associations between the genotypes and sex phenotypes of the 127 flowering F_1_ individuals were also checked by simple interval mapping (SIM) using R/qtl v1.70 [21]. The significance of each linkage was tested based on the likelihood-ratio statistic (LOD). The LOD scores were calculated by “scanone” with the following options: method="em", n.perm=1000. The LOD threshold for linkage was set to a 95% confidence level (alpha = 0.05).

The dot plot between the chromosome 3 of Waka1 (female) haplotype 1 and Kita1 (male) haplotype 1 was generated using D-Genies [26].

**4.2 Mapping coverage analysis**

To identify regions with different alignment depths between male and female individuals, Illumina short reads from four pairs of male and female were examined: the male parent Kita1 and the female parent Waka1, and three pairs of male and female individuals collected from northern, central, and southern Japan. For quality control of the Illumina short reads, adapters and reads of <50 bp, as well as low-quality reads with an average read quality score <20, were removed with FaQCs v2.10 [6]. The filtered short reads were aligned to each reference genome. Sequence alignment was conducted using BWA v0.7.17-r1188 [17] with the BWA-MEM algorithm setting. The alignment depths were calculated with Samtools v1.16.1 [18]; in this step, reads with mapping quality ≥ 40 were counted. The alignment depths were normalized by dividing by the mean depth of all positions on the chromosomes. To reduce the effect of error on the depth, a sliding window approach was employed (window size = 150 kb, step size = 10 kb). We identified regions in which the male depth was 0.5 (half of mean normalized depth) ± 0.15 and the female depth was 1 (mean normalized depth) ± 0.15, and. regions in which the male depth was 0.5 (half of mean normalized depth) ± 0.15 and the female depth was < 0.15.

**4.3 Confirmation of the assembly continuity around the X- and Y- specific regions**

To confirm the assembly continuity around the X- and Y- specific regions, we reconducted *De novo* assembly using another software and coverage confirmation using long reads generated by Oxford Nanopore Technology. The filtered long reads of Waka1 (female) and Kita1 (male) were assembled using Hifiasm v0.25.0-r726 [27] with the option --ont. This assembly generated two phased assemblies for Waka1 (female): 744 contigs with N50 of 34,733,904 bp and a total size of 471.8 Mb for the haplotype 1, and 40 contigs with N50 of 36,835,416 bp and a total size of 406.6 Mb for the haplotype 2. This assembly also generated two phased assemblies for Kita1 (male): 1,446 contigs with N50 of 35,381,014 bp and a total size of 459.7 Mb for haplotype 1, and 87 contigs with N50 of 32,591,320 bp and a total size of 392.1 Mb for haplotype 2. The new assemblies by Hifiasm included telomere-to-telomere contigs, and the comparison with the constructed assemblies of Kita1 (male) and Waka1 (female) suggested the constructed assemblies are not software dependent (Fig B). The dot plots were generated using D-Genies [26].

In addition, Y-specific regions were located on a continuous contig in both assemblies by PECAT and Hifiasm (Fig Ca). X-specific regions were also located on a continuous contig in both assemblies by PECAT and Hifiasm (Fig Da). For coverage confirmation using long reads generated with Oxford Nanopore Technology, the filtered long reads of Waka1 (female) and Kita1 (male) were aligned to the female and male reference genomes, respectively using minimap2 v 2.17-r941 [28]. The alignment depths were calculated with Samtools v1.16.1 [18] with the options -Q 40 and -m 100. The Y-regions were covered by male long reads (Fig Cb), and the X-regions were covered by female long reads (Fig Db). In addition, both of Y- and X- regions were covered without gaps of the long reads (Fig Cc, Dc). Close-up view of aligned long reads were generated by Integrative Genomics Viewer v2.16.2 [29].


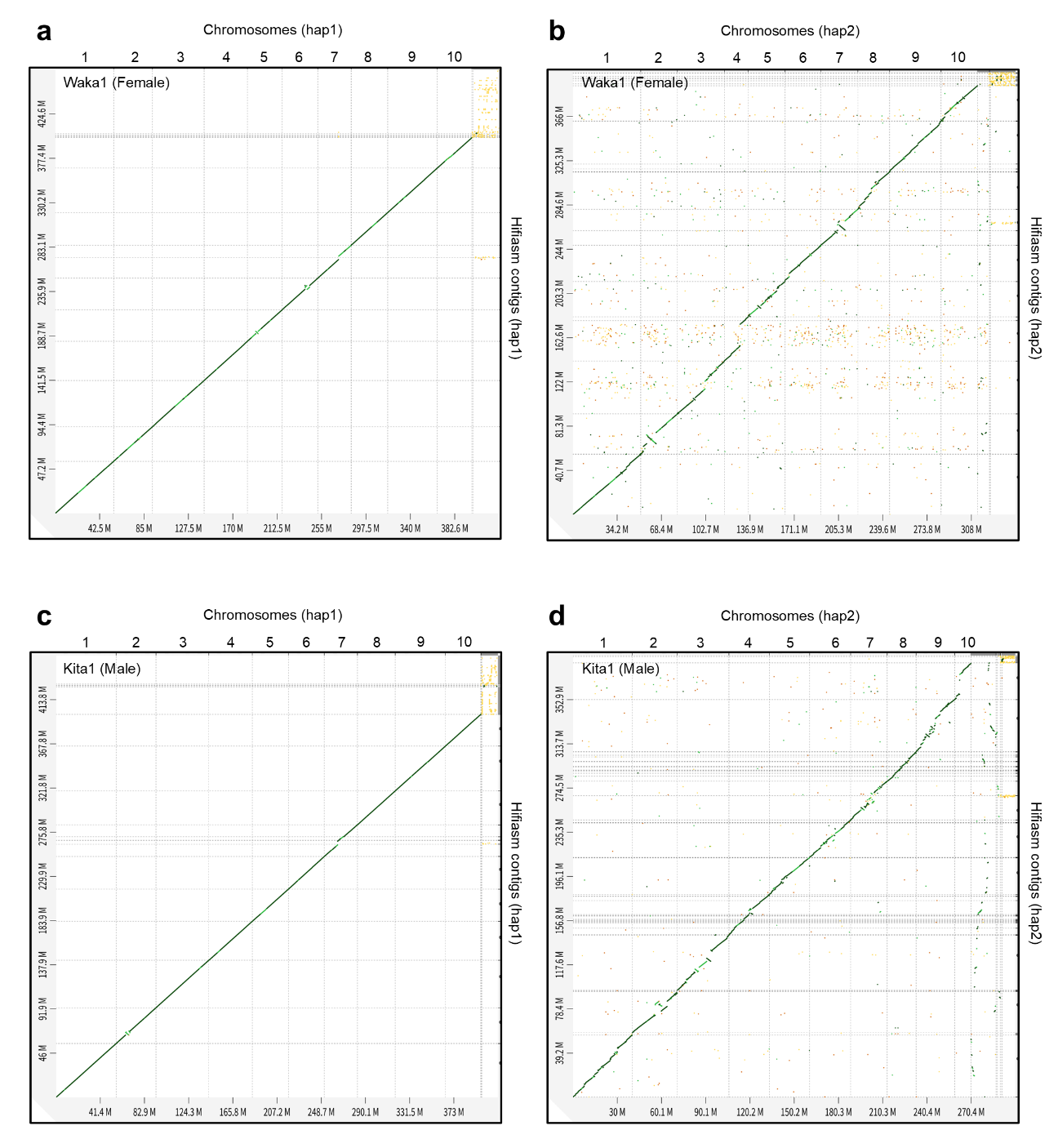


**Fig B.** **Comparison of assemblies from two different assembly tools, PECAT and Hifiasm. (a)** Comparison between the chromosomal-level haplotype 1 assembly generated by PECAT and the haplotype 1 assembly generated by Hifiasm for Waka1 (female). **(b)** Comparison between the chromosomal-level haplotype 2 assembly generated by PECAT and the haplotype 2 assembly generated by Hifiasm for Waka1 (female). **(c)** Comparison between the chromosomal-level haplotype 1 assembly generated by PECAT and the haplotype 1 assembly generated by Hifiasm for Kita1 (male). **(d)** Comparison between the chromosomal-level haplotype 2 assembly generated by PECAT and the haplotype 2 assembly generated by Hifiasm for Kita1 (male).


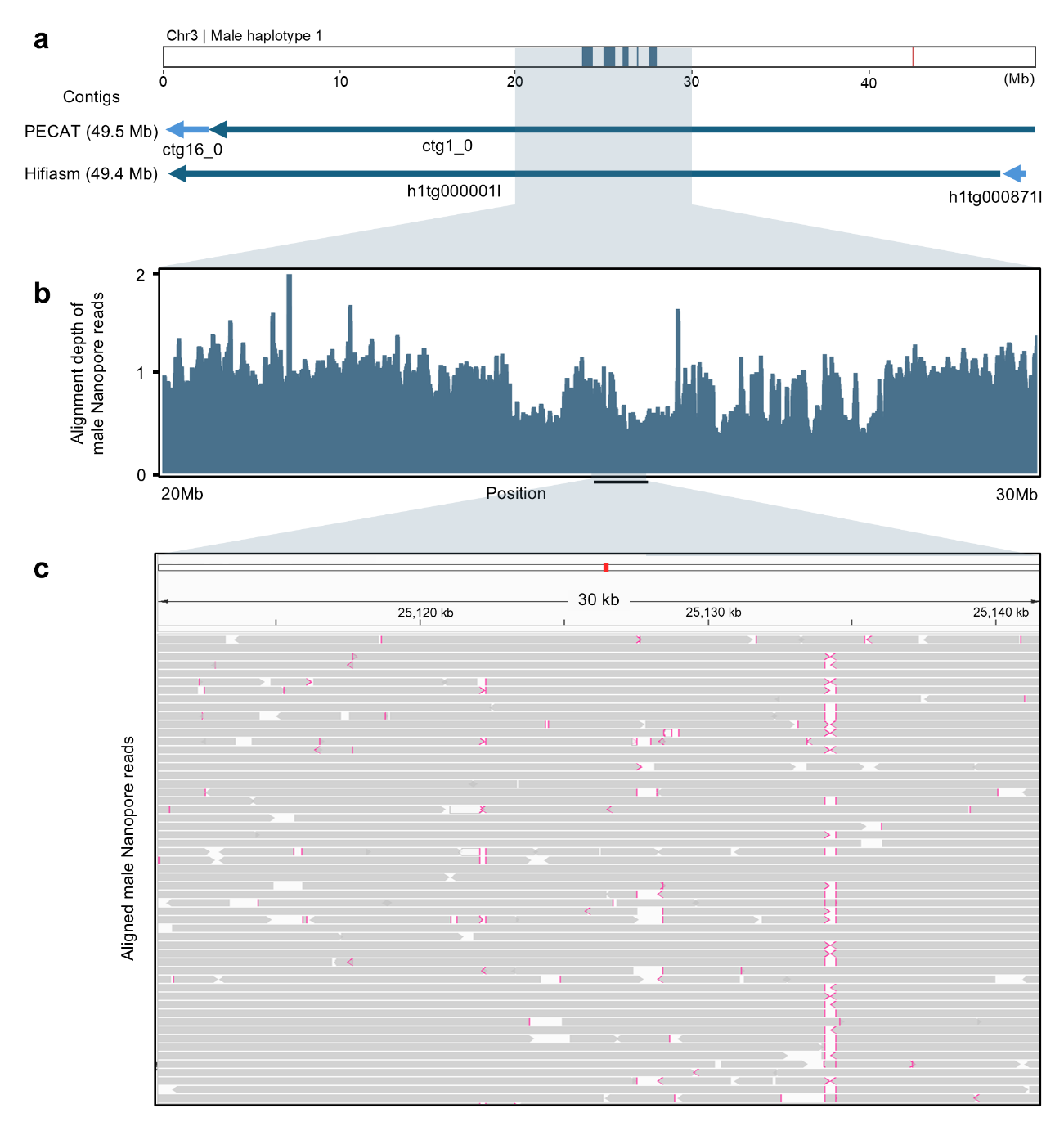


**Fig C.** **Aligned ONT reads showed that Y-specific regions are contiguous. (a)** Positions of contigs on the Y chromosome. Two different assembly tools, PECAT and Hifiasm, generated continuous contigs across Y-specific regions. **(b)** Alignment depth of male long reads generated with Oxford Nanopore Technology on the Y chromosome. No gaps were observed in Y-specific regions. **(c)** Close-up view of aligned long reads. Y-specific regions showed uninterrupted coverage with no gaps.


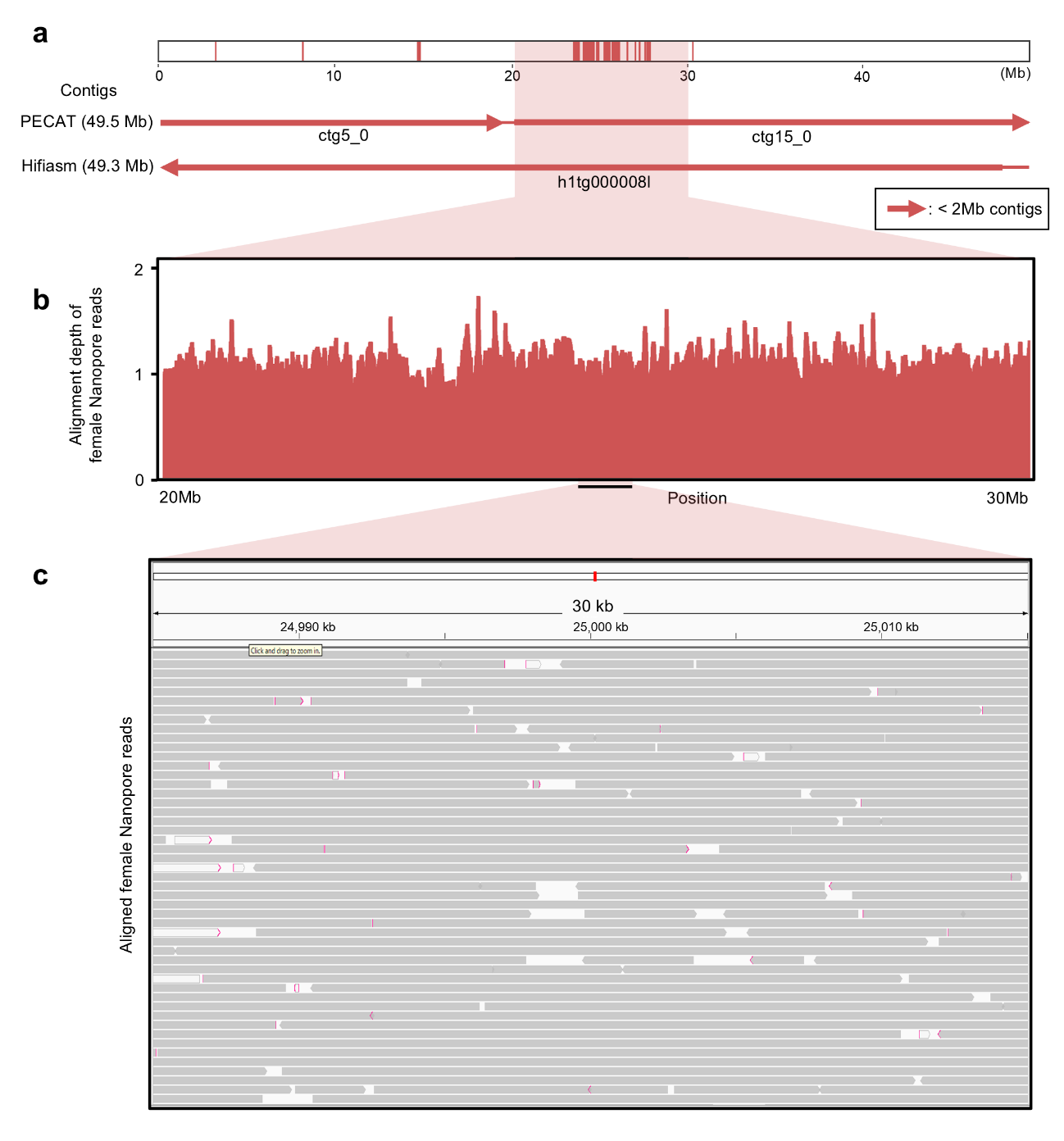


**Fig D.** **Aligned ONT reads showed that X-specific regions are contiguous. (a)** Positions of contigs on the X chromosome. Two different assembly tools, PECAT and Hifiasm, generated continuous contigs across X-specific regions. **(b)** Alignment depth of female long reads generated with Oxford Nanopore Technology on the X chromosome. No gaps were observed in X-specific regions. **(c)** Close-up view of aligned long reads. X-specific regions showed uninterrupted coverage with no gaps.

**4.4 SDRs confirmation by SDpop and RADSex**

The SDRs were also estimated using two previously reported methods, SDpop [30] and RADSex [31].

For SDpop analysis, we used Illumina short reads from five females and five males from KZGW population. For quality control of the Illumina short reads, adapters and reads of <50 bp, as well as low-quality reads with an average read quality score <20, were removed with FaQCs v2.10 [6]. The filtered reads were aligned to each reference genome: Waka1 (female) and Kita1 (male) haplotype 1. The alignment was conducted using BWA v0.7.17-r1188 [17] with the BWA-MEM algorithm setting. SNP-based genotypes were obtained using Bcftools v1.15 [18] with the following commands: (i) mpileup command with the option “-a DP,AD,SP -B -Q 18 -C 50”; (ii) call command with the option “-P 0 -m -a GQ,GP”. After obtaining SNP-based genotypes, the vcf files were converted to the SDpop format using popsum command in SDpop [30]. Finally, posterior probability for segregation types were calculated using sdpop command in SDpop with the option, “e x 0 0 s”. The distribution of the posterior probability values was visualized by R v4.1.1. In this analysis, only the polymorphic sites located within coding regions of orthologous gene pairs of chromosome 3Y (from Kita1 haplotype 1) and chromosome 3X (from Waka1 haplotype 1) were used. The orthologous gene pairs (<1 × 10^−50^ in BLASTP analyses, max_target_seqs = 2) were obtained following [32–34].

For RADSex analysis, we used RAD-seq data from 127 flowering F_1_ individuals. Based on the filtered RAD-seq data and sex phenotypes, probability of association with sex, -log10(*p*), were calculated by RADSex v1.2.0 [31] with the following commands: (i) radsex process command with the option “--min-depth 1”; (ii) radsex distrib command with the option “--min-depth 5 –groups M,F”; (iii) radsex signif command with the option “--min-depth 5 –groups M,F”. The distributions of probability of association with sex, -log_10_(*p*), were visualized using sgtr package in R 4.1.1.

Based on the results of our association analysis, and mapping coverage analysis and SDRs detection analysis, regions including depth differences within the 23–29 Mb interval on X chromosome and within the 20–30 Mb interval on Y chromosomes were defined as X and Y-specific regions, respectively. The start position (23,395,001 bp) to end position (28,209,000 bp) of regions, in which the male depth was 0.5 (half of mean normalized depth) ± 0.15 and the female depth was 1 (mean normalized depth) ± 0.15, in X chromosome were defined as X-specific regions. The start position (23,759,001 bp) to end position (28,256,000 bp) of regions, in which the male depth was 0.5 (half of mean normalized depth) ± 0.15 and the female depth was < 0.15, in Y chromosome were defined as Y-specific regions. In this step, start and end positions of the depth differences were detected using higher-resolution sliding windows (window size = 20 kb, step size = 1 kb).

**5. Genome structures of the X and Y chromosomes**

**5.1 Gene density and repetitive sequence accumulation in X- and Y-specific regions**

Information about gene density and repetitive sequence accumulation was obtained from gene annotations and predicted repetitive sequences of the Waka1 (female) and Kita1 (male) haplotype 1 assembly. Repetitive sequences including *Copia* LTR retrotransposons, Ty3 (*gypsy*) LTR retrotransposons, *hAT* TIR transposons, and Helitrons were predicted using EDTA v2.1.0. Gene density and retrotransposon accumulation were visualized using the “jcvi.graphics.landscape heatmap” function of the jcvi package [24].

**5.2 Estimation of divergence between X- and Y-linked gametologs**

Synonymous divergence (*d_S_*) between X- and Y-linked gametologs was obtained following [32–34] with slight modifications. Orthologous or paralogous gene pairs (<1 × 10^−50^ in BLASTP analyses, max_target_seqs = 2) obtained from three comparison: female individual (Waka1) and male individual (Kita1) of *D. tokoro*, male (Kita1) *D. tokoro* and *D. alata* [35], male (Kita1) *D. tokoro* and *D. rotundata* [16]. The gene pairs were aligned in codon frames using MAFFT v7.475 [36] with the option “--localpair --maxiterate 1000” and Pal2Nal v14 [37]. Based on the in-codon-frame alignments, we calculate the Jukes and Cantor corrected values of *d_S_* using KaKs_Calculator v2.0 [38]. Gene pairs located in Y-specific regions of male *D. tokoro* and the X chromosome of female *D. tokoro* were defined as XY gametologs. The gametologs with *d_S_* values > 1 were removed from XY gametologs. The comparison and the distributions of *d_S_* values were visualized by R v4.4.1. In the distribution of *d_S_* values of all gene pairs across X and Y chromosomes, we applied a threshold of *d_S_* < 0.5 based on the maximum *d_S_* values reported in previous studies [32,39], and less stringent threshold of *d_S_* < 1.0.

**5.3** **Comparison of sex chromosomes in the genus *Dioscorea***

To compare the sex chromosomes of *D. tokoro* (male), *D. alata* [35] and *D. rotundata* [16], Gene order–based synteny of chromosomes was detected using MCscan [24]. The orthologous regions were identified using the “jcvi.compara.catalog ortholog” and the “jcvi.compara.synteny screen” function with at least 30 collinear gene blocks. The detected collinearity was visualized using the “jcvi.graphics.karyotype” function.

**6. Identification of candidate genes for sex determination**

**6.1 Identification of highly expressed genes in male flowers during early stages of development**

Differential expression analysis was performed using the filtered RNA-seq data from male flowers, female flowers, and non-reproductive organs. The trimmed RNA-seq reads were aligned to the Kita1 (male) haplotype 1 assembly using HISAT v2.2.1 [11]. The mapped reads were counted with the featureCounts function in Subread v2.0.1 [40]. The minimum fragment length was set to 19, and the attribute type was set to transcript id. Differential expression analysis was performed using DESeq2 v3.15 [41] in R v4.1.1 for two comparisons: male vs. female flowers at three early stages of development (inflorescence stages 0, 1, and 2) and male flowers at three stages of early development vs. non-reproductive organs. The false discovery rate threshold was set to 0.05.

**6.2 Identification of candidate genes for sex determination**

As the first step in candidate gene identification, 56 genes located on Y-specific regions were identified (Table 1). In the second step, among the genes and miRNA on Y-specific regions, 14 genes that showed statistically significant differential expression during three early stages of male vs. female flower development were selected based on differential expression analysis using negative binomial generalized linear models (*p* < 0.05). Ten genes were also identified that were significantly upregulated in male vs. female flowers during three stages of early development based on the log_2_ ratio of the mean of normalized counts in each group (log_2_FC > 2). In the third step, among the ten genes, two genes were identified that showed statistically significant differential expression during three stages of early development compared to non-reproductive organs (*p* < 0.05). The two genes were also significantly upregulated in male flowers during three stages of early development compared to non-reproductive organs (log_2_FC > 2). Finally, two Y-specific genes were selected with significantly upregulated expression in male flowers during three stages of early development in both transcriptome comparisons (*p* < 0.05 and log_2_FC > 2). The similarity of the candidate gene products was compared to known proteins using BLASTX with the Swiss-Prot function.

**6.3 PCR amplification of the candidate genes**

The male specificity of the two candidate genes was also confirmed by PCR amplification using five females and five males from two wild populations, one in northern Japan and one in southern Japan. Genomic DNA was extracted from the samples using a Maxwell RSC Plant DNA Kit (Promega, Madison, WI, USA). DNA fragments of *BLH9* and *HSP90* were amplified with Quick Taq HS DyeMix (TOYOBO, Osaka, Japan) using primers for the first exons of *BLH9* and *HSP90*. The *xanthine dehydrogenase* (*Xdh*) gene located on the pseudoautosomal region of chromosome 3 was also amplified as a control. The *Xdh* gene has been used for nuclear phylogenetic analysis of the genus *Dioscorea* [42], and we obtained the *Xdh* sequence of *D. tokoro* based on the *Xdh* sequence of the related species *D. caucasica* (GenBank: KY712749.1) using BLASTX. All primers used in this step are listed in Table H.

**6.4 Phylogenetic analysis of candidate genes and their homologous genes**

Phylogenetic analysis of *BLH9* was performed using protein sequences of TALE superfamily proteins from *A. thaliana* and its putative homologs from *D. tokoro.* The sequences of *A. thaliana* TALE superfamily proteins were obtained from The Arabidopsis Information Resource (TAIR) (Table I) based on [43]. The putative homologues (<1 × 10^−10^ in BLASTP analyses, max_target_seqs = 5) of TALE superfamily proteins were obtained from the protein sequences of male individual (Kita1) of *D. tokoro*. Phylogenetic analysis of *HSP90* was performed using the mRNA sequences of *HSP90* family proteins from *A. thaliana* and its putative homologs from *D. tokoro.* The sequences of *A. thaliana* HSP90 family proteins were obtained from NCBI (Table J) based on [44]. The putative homologues (<1 × 10^−10^ in TBLASTN analyses, max_target_seqs = 20) of HSP90 family proteins were obtained from the mRNA sequences of male individual (Kita1) of *D. tokoro*. The sequences were aligned using MAFFT v7.475 with the L-INS-i strategy [45]. Based on the alignment, a phylogenetic tree was constructed with the maximum likelihood method using iqtree v2.1.2 [46]. The bootstrap values were calculated by the ultrafast bootstrap approximation with 1,000 replications. The tree was visualized using Interactive Tree Of Life (iTOL) v7.0 [47].

**7. Identification of candidate miRNAs for sex determination**

**7.1 Sequence processing**

For quality control of the small RNA-seq data, adapters and low-quality reads with an average read quality score > 20 were removed with FaQCs v2.10 [6]. Reads shorter than 19 bp and longer than 25 bp were removed using Seqkit v2.3.0 [48]. To predict miRNAs using miRDeep-P2 v1.1.4 [49], the filtered reads were preprocessed into the designated format. The filtered reads were parsed into FASTA format with Seqkit v2.3.0, and redundant sequences were removed.

**7.2 miRNA prediction and annotation**

The miRNAs were predicted as described previously [50] with several modifications. Novel miRNAs were detected from preprocessed reads using the miRDP2-v1.1.4_pipeline.bash script of miRDeep-P2 v1.1.4. The predicted miRNA sequences and their corresponding precursor and primary information were extracted using the perl script parse_miRDP2_prediction.pl [50]. The miRNA sequences were annotated to the Kita1 (male) haplotype 1 assembly. First, a BLAST database was created from the *D. tokoro* reference genome using the makeblastdb function of Blast+ v2.2.31 [51]. miRNAs hits in the *D. tokoro* BLAST database were selected using the blastn function of BLAST+ v2.2.31 with output format 6. To eliminate missing candidate miRNAs, the word size was set to 7 and the *e*-value threshold was set to 1,000. The output tab file was transferred to gff3 format using the blast2gff.py script of genomeGTFtools v1.3 [52].

**7.3 Identification of highly expressed miRNAs in male flowers**

The trimmed small RNA-seq reads were aligned to the predicted miRNA references with Bowtie v1.3.1 [53]. The mapped reads were counted using the perl script bam2ref_counts.pl, and the read counts data for each sample were combined using the perl script combine_htseq_counts.pl [50]. Differential expression analysis was performed using DESeq2 v3.15 [41] in R v4.1.1 for two comparisons: male vs. female flowers at three stages of early development (inflorescence stages 0, 1, and 2) and male flowers at three stages of early development vs. non-reproductive organs. The false discovery rate threshold was set to 0.05.

**7.4 Identification of candidate miRNAs for sex determination**

As the first step in candidate primary miRNA identification, one primary miRNA was located on Y-specific regions within 46 primary miRNAs of Y chromosome (S4 Table). Among the Y-specific miRNA, no miRNA was highly expressed in male vs. female flowers during early stages of development (*p* < 0.05).

**8. Overexpression of *BLH9* and *AtBLH9* in *Arabidopsis thaliana***

**8.1 Phylogenetic analysis of *D. tokoro* BLH9 and TALE superfamily proteins**

Phylogenetic analysis of *D. tokoro BLH9* and its homologs was performed using the sequences of BLH9, putative homologues of BLH9 from monocotyledonous species (*Asparagus officinalis, Dioscorea alara, D. rotundata, Oryza sativa* and *Phoenix dactylifera*) and TALE superfamily proteins from *A. thaliana.* The sequences of *A. thaliana* TALE superfamily proteins were obtained from The Arabidopsis Information Resource (TAIR) (Table I) based on [43]. The putative homologues (<1 × 10^−10^ in BLASTP analyses, max_target_seqs = 2) of *BLH9* were obtained from the protein sequences of the five species: *Asparagus officinalis* (NCBI RefSeq assembly; GCF_001876935.1)*, Dioscorea alara* [35]*, D. rotundata* [16]*, Oryza sativa* (NCBI RefSeq assembly; GCF_034140825.1) and *Phoenix dactylifera* (NCBI RefSeq assembly; GCF_009389715.1)*.* The sequences were aligned using MAFFT v7.475 with the L-INS-i strategy [45]. Based on the alignment, a phylogenetic tree was constructed with the maximum likelihood method using iqtree v2.1.2 [46]. The bootstrap values were calculated by the ultrafast bootstrap approximation with 1,000 replications. The tree was visualized using Interactive Tree Of Life (iTOL) v7.0 [47].

**8.2 Plant materials**

All *A. thaliana* lines are in the Col-0 accession background. Surface-sterilized *A. thaliana* seeds were incubated at 4°C in the dark for 2 to 5 days and grown on Murashige and Skoog (MS) medium (1/2 MS, 0.5% sucrose, B5 vitamin solution, 2 mM MES, pH 5.7) under controlled conditions (23°C, 10-h photoperiod). After germination, the plants were transferred to soil and grown under controlled conditions (23°C, 16-h photoperiod, 57 µmol m^−2^ s^−1^ of light).

**8.3 Cloning and plasmid construction**

Two full-length cDNA clones were used for In-Fusion cloning: *BLH9* from inflorescence stage 2 of the *D. tokoro* Kita1 male individual and *AtBLH9* from the first internode of *A. thaliana* Col-0. Total RNA was extracted from the samples using a Maxwell RSC Plant RNA Kit (Promega) and reverse-transcribed into cDNA using a ReverTra Ace qPCR RT Kit FSQ-101 (TOYOBO). *BLH9* fragment was amplified from the *D. tokoro* cDNA using KOD FX Neo (TOYOBO). *AtBLH9* fragment was amplified from *A. thaliana* cDNA using PrimeSTAR GXL DNA Polymerase (Takara Bio). After purification using NucleoSpin Gel and PCR Clean-up (Macherey-Nagel), each cDNA fragment was cloned into EcoRI and BamHI sites of the pBICP35 binary vector, which contains the CaMV35S promoter [54] using an In-Fusion HD Cloning Kit (Takara Bio). The insertion was confirmed using Quick Taq HS DyeMix (TOYOBO), and the inserted sequences were confirmed by DNA sequencing (Eurofin Genomics, Tokyo, Japan). All primers used in this step are listed in Table K.

**8.4 Plant transformation**

*pBICP35::BLH9* and *pBICP35::AtBLH9* were transformed into *Agrobacterium tumefaciens* strain *GV3101::pMP90* by electroporation. *A. thaliana* transformation was performed by the floral dip method with *A. tumefaciens* strains carrying the binary expression plasmids, and kanamycin-resistant T_1_ plants were selected. T_2_ lines that showed a 3:1 segregation ratio for kanamycin resistance were selected, as they were thought to contain a single T-DNA. The selected T_2_ lines and Col-0 were used to examine inflorescence phenotypes.

**8.5 RT-qPCR**

To examine inflorescence phenotypes, 16 plants each were grown from eight lines: Col-0, three T_2_ lines transformed with the plasmid bearing the *BLH9* construct, and four T_2_ lines transformed with the plasmid bearing the *AtBLH9* construct. To eliminate the effects of kanamycin selection on plant growth, all seeds of T_2_ plants and Col-0 were grown in MS medium without kanamycin. The expression levels of *BLH9* and *AtBLH9* in T_2_ plants were confirmed by RT-qPCR (Data S4). Total RNA was extracted from each plant using a Maxwell RSC Plant RNA Kit (Promega), and cDNAs were synthesized using a ReverTra Ace qPCR RT Kit FSQ-101 and ReverTra Ace qPCR RT Master Mix FSQ-201S (TOYOBO). RT-qPCR was conducted using PowerUp SYBR Green Master Mix and a StepOne Real-Time PCR System with the following settings: Quantitation comparative C_T_ (ΔΔC_T_) and Standard cycling mode with StepOne Software v2.3 (Applied Biosystems, Waltham, MA, USA). The relative expression levels of the inserted genes were calculated by the 2^−ΔΔCT^ method, and 2^−ΔΔCT^ values were < 2 without overexpression (S30 Fig). T_2_ plants were separated into four groups: *BLH9* Control (2^−ΔΔCT^ ≤ 2), *BLH9* *OX* (2^−ΔΔCT^ > 2), *AtBLH9* Control (2^−ΔΔCT^ ≤ 2), and *AtBLH9* *OX* (2^−ΔΔCT^ > 2). *Ubiquitin C* (*UBC*) was used as a reference gene. The primers used in RT-qPCR are listed in Table K.

**8.6 Measurement of inflorescence phenotypes**

At 30 days after seedlings were transplanted to soil, three phenotypes were measured: inflorescence height, mean fruit length, and internode length. Inflorescence height represents the length of the main inflorescence stem from the rosette to apex. The mean fruit length was obtained from 10 fruits of the main inflorescence at positions 2 to 11 counting from the lowest fruit on the main stem. When there were fewer than 10 fruits, all fruits at positions 2 to 11 were examined to obtain mean fruit length. Aborted buds were skipped. Internode length represents the stem length between two fruits. For each plant, 10 internodes were obtained from the main inflorescence at positions 2 to 11 counted from the lowest fruit on the main stem. When there were fewer than 10 internodes, all internodes at positions 2 to 11 were included to obtain internode length data. All phenotypic data are listed in Data S5, S6, and S7. The mean inflorescence heights and mean fruit lengths were compared by Wilcoxon rank sum test between the five groups: Col-0, *BLH9* Control, *BLH9* *OX*, *AtBLH9* Control, and *AtBLH9* *OX*. The variations in internode lengths were compared by performing an *F*-test between the five groups. The *p*-values were adjusted by Bonferroni correction.

**Table A. Sampling sites of *Dioscorea tokoro***

| Sample | Sex | Site | Latitude | Longitude |
| --- | --- | --- | --- | --- |
| **1.2 Whole-genome sequencing of female and male individuals using Oxford Nanopore Technology** | | | | |
| Waka1 | Female | Tahara, Wakayama Pref., Japan | 33°32'16.8"N | 135°51'36.0"E |
| Kita1 | Male | Kitakami, Iwate Pref., Japan | 39°17'42.0"N | 140°53'45.6"E |
| **1.3 Illumina library construction and sequencing of male and female individuals** | | | | |
| Waka1 | Female | Tahara, Wakayama Pref., Japan | 33°32'16.8"N | 135°51'36.0"E |
| Kita1 | Male | Kitakami, Iwate Pref., Japan | 39°17'42.0"N | 140°53'45.6"E |
| Female from KTKM (northern area) | Female | Kitakami, Iwate Pref., Japan | 39°18'25.0"N | 140°54'07.0"E |
| Male from KTKM (northern area) | Male | Kitakami, Iwate Pref., Japan | 39°18'25.0"N | 140°54'07.0"E |
| Female from SHG (central area) | Female | Koka, Shiga Pref., Japan | 34°56'24.1"N | 136°13'05.3"E |
| Male from SHG (central area) | Male | Koka, Shiga Pref., Japan | 34°56'24.1"N | 136°13'05.3"E |
| Female from FKOK (southern area) | Female | Kasuya, Fukuoka Pref., Japan | 33°38'08.7"N | 130°30'38.2"E |
| Male from FKOK (southern area) | Male | Kasuya, Fukuoka Pref., Japan | 33°38'08.7"N | 130°30'38.2"E |
| Females from KZGW | Female | Kozagawa, Wakayama Pref., Japan | 33°32'24.1"N | 135°46'29.3"E |
| Males from KZGW | Male | Kozagawa, Wakayama Pref., Japan | 33°32'24.1"N | 135°46'29.3"E |
| **1.5 RNA libraries and sequencing** |  |  |  |  |
| Kita1 | Male | Kitakami, Iwate Pref., Japan | 39°17'42.0"N | 140°53'45.6"E |
| Female from KTKM (northern area) | Female | Takizawa and Kitakami, Iwate Pref., Japan |  |  |
| Male from KTKM (northern area) | Male | Takizawa and Kitakami, Iwate Pref., Japan |  |  |
| **1.6 Small RNA libraries and sequencing** |  |  |  |  |
| Female from SHG (central area) | Female | Koka, Shiga Pref., Japan | 34°56'00.8"N | 136°13'30.5"E |
| Male from SHG (central area) | Male | Koka, Shiga Pref., Japan | 34°56'00.8"N | 136°13'30.5"E |
| **6.3 PCR amplification of the candidate genes** |  |  |  |  |
| Female from HNMK (northern area) | Female | Hanamaki, Iwate Pref., Japan | 39°22'10.0"N | 141°09'16.0"E |
| Male from HNMK (northern area) | Male | Hanamaki, Iwate Pref., Japan | 39°22'10.0"N | 141°09'16.0"E |
| Female from KMMT (southern area) | Female | Kumamoto, Kumamoto Pref., Japan | 32°53'34.8"N | 130°39'22.7"E |
| Male from KMMT (southern area) | Male | Kumamoto, Kumamoto Pref., Japan | 32°53'34.8"N | 130°39'22.7"E |

**Table B. Summary of Illumina short reads from the whole genome.**

| Sample | Original fastq |  |  | Filtered fastq |  |  | Genome  coverage | Sequencing  platform | Accession no. |
| --- | --- | --- | --- | --- | --- | --- | --- | --- | --- |
|  | Number of  reads | Total base  pairs (Gbp) |  | Number of  reads | Total base  pairs (Gbp) |  |  |  |  |
| Waka1 (female) | 185,338,998 | 27.9 |  | 180,836,694 | 27.1 |  | 69.9× | HiSeqX | SRR32328377  SRR32328330 |
| Kita1 (male) | 350,412,788 | 62.4 |  | 347,141,258 | 61.6 |  | 159.0× | MiSeq, HiseqX | DRX333479  DRX335960 |
| Female (northern area) | 41,867,422 | 6.28 |  | 41,769,731 | 5.95 |  | 15.3× | HiSeqX | SRR32328338 |
| Male (northern area) | 54,337,944 | 8.15 |  | 54,187,471 | 7.81 |  | 20.1× | HiSeqX | SRR32328337 |
| Female (central area) | 64,740,690 | 9.71 |  | 64,587,572 | 8.95 |  | 23.1× | HiSeqX | SRR32328334 |
| Male (central area) | 71,080,268 | 10.7 |  | 70,966,105 | 9.73 |  | 25.1× | HiSeqX | SRR32328333 |
| Female (southern area) | 72,491,790 | 10.9 |  | 72,369,383 | 9.76 |  | 25.2× | HiSeqX | SRR32328336 |
| Male (southern area) | 47,610,492 | 7.14 |  | 47,496,663 | 6.72 |  | 17.3× | HiSeqX | SRR32328335 |
| Five females (KZGW) | 59,479,346 | 8.92 |  | 58,822,182 | 8.72 |  | 22.5× | NovaSeq X Plus | SRR36002286 |
|  | 85,253,992 | 12.8 |  | 83,960,650 | 12.0 |  | 31.0× | NovaSeq X Plus | SRR36002285 |
|  | 55,715,376 | 8.36 |  | 55,049,756 | 8.11 |  | 20.9× | NovaSeq X Plus | SRR36002284 |
|  | 72,253,160 | 10.8 |  | 71,394,716 | 10.4 |  | 26.7× | NovaSeq X Plus | SRR36002283 |
|  | 42,721,792 | 6.41 |  | 42,277,156 | 6.23 |  | 16.0× | NovaSeq X Plus | SRR36002282 |
| Five males (KZGW) | 59,910,524 | 8.99 |  | 59,104,690 | 8.74 |  | 22.5× | NovaSeq X Plus | SRR36002281 |
|  | 43,894,216 | 6.58 |  | 43,390,150 | 6.41 |  | 16.5× | NovaSeq X Plus | SRR36002280 |
|  | 49,311,592 | 7.40 |  | 48,592,238 | 7.21 |  | 18.6× | NovaSeq X Plus | SRR36002279 |
|  | 58,922,134 | 8.84 |  | 58,299,690 | 8.57 |  | 22.1× | NovaSeq X Plus | SRR36002278 |
|  | 63,429,112 | 9.51 |  | 62,730,110 | 9.09 |  | 23.4× | NovaSeq X Plus | SRR36002277 |

Genome coverage was estimated based on the genome size of *D. tokoro* (388 Mb).

**Table C. RNA-seq data generated from libraries constructed from different tissues of *Dioscorea tokoro*.**

| Sample | Sex | Original fastq |  |  | Filtered fastq |  |  | Sequencing platform | Accession no. |
| --- | --- | --- | --- | --- | --- | --- | --- | --- | --- |
|  |  | Number of reads | Total base pairs  (Gbp) |  | Number of reads | Total base pairs  (Gbp) |  |  |  |
| Inflorescence stage 0 | Male | 24,276,259 | 1.81 |  | 24,141,016 | 1.79 |  | NextSeq500 | SRR32328332 |
| Inflorescence stage 1 | Male | 24,765,787 | 1.84 |  | 24,637,348 | 1.83 |  | NextSeq500 | SRR32328331 |
| Inflorescence stage 2 | Male | 27,102,871 | 2.02 |  | 26,962,941 | 2.00 |  | NextSeq500 | SRR32328328 |
| Bud | Male | 26,096,017 | 1.94 |  | 25,945,931 | 1.93 |  | NextSeq500 | SRR32328327 |
| Flower | Male | 24,042,221 | 1.79 |  | 23,890,712 | 1.77 |  | NextSeq500 | SRR32328326 |
| Inflorescence stage 0 | Female | 26,613,535 | 1.98 |  | 26,475,567 | 1.97 |  | NextSeq500 | SRR32328325 |
| Inflorescence stage 1 | Female | 24,109,359 | 1.80 |  | 23,993,280 | 1.78 |  | NextSeq500 | SRR32328324 |
| Inflorescence stage 2 | Female | 25,689,180 | 1.91 |  | 25,564,897 | 1.90 |  | NextSeq500 | SRR32328323 |
| Bud | Female | 26,346,910 | 1.96 |  | 26,211,849 | 1.95 |  | NextSeq500 | SRR32328322 |
| Flower | Female | 24,003,719 | 1.79 |  | 23,896,870 | 1.78 |  | NextSeq500 | SRR32328321 |
| Vegetative shoot apex | Kita1 (male) | 23,051,829 | 1.72 |  | 22,935,643 | 1.70 |  | NextSeq500 | SRR32328320 |
| Leaf | Kita1 (male) | 24,299,715 | 1.81 |  | 24,144,324 | 1.79 |  | NextSeq500 | SRR32328319 |
| Stem | Kita1 (male) | 19,216,778 | 1.43 |  | 19,079,127 | 1.42 |  | NextSeq500 | SRR32328317 |
| Root apex | Kita1 (male) | 23,592,409 | 1.76 |  | 23,473,872 | 1.74 |  | NextSeq500 | SRR32328316 |
| Rhizome bud | Kita1 (male) | 26,479,350 | 1.97 |  | 26,346,195 | 1.96 |  | NextSeq500 | SRR32328315 |
| Rhizome root | Kita1 (male) | 22,952,532 | 1.71 |  | 22,819,053 | 1.70 |  | NextSeq500 | SRR32328314 |
| Rhizome stem | Kita1 (male) | 25,506,326 | 1.90 |  | 25,375,473 | 1.89 |  | NextSeq500 | SRR32328313 |
| Rhizome storage | Kita1 (male) | 25,238,872 | 1.88 |  | 25,120091 | 1.87 |  | NextSeq500 | SRR32328312 |

**Table D. Small RNA-seq data generated from libraries constructed from different tissues of *Dioscorea tokoro*.**

| Sample | Sex | Original fastq |  |  | Filtered fastq |  |  | Sequencing platform | Accession no. |
| --- | --- | --- | --- | --- | --- | --- | --- | --- | --- |
|  |  | Number of reads | Total base  pairs (Gbp) |  | Number of reads | Total base  pairs (Gbp) |  |  |  |
| Inflorescence stage 0 | Male | 35,195,175 | 1.35 |  | 35,092,102 | 1.34 |  | NovaSeq6000 | SRR32328179 |
| Inflorescence stage 1 | Male | 50,547,350 | 1.79 |  | 50,377,342 | 1.78 |  | NovaSeq6000 | SRR32328178 |
| Inflorescence stage 2 | Male | 17,635,303 | 0.64 |  | 17,575,869 | 0.64 |  | NovaSeq6000 | SRR32328177 |
| Bud | Male | 48,611,510 | 1.71 |  | 48,424,940 | 1.70 |  | NovaSeq6000 | SRR32328176 |
| Flower | Male | 40,153,242 | 1.40 |  | 39,999,479 | 1.39 |  | NovaSeq6000 | SRR32328174 |
| Leaf | Male | 52,426,728 | 1.89 |  | 52,155,143 | 1.88 |  | NovaSeq6000 | SRR32328173 |
| Stem | Male | 32,635,855 | 1.20 |  | 32,507,263 | 1.19 |  | NovaSeq6000 | SRR32328172 |
| Inflorescence stage 0 | Female | 5,404,699 | 0.21 |  | 5,390,903 | 0.21 |  | NovaSeq6000 | SRR32328171 |
| Inflorescence stage 1 | Female | 42,777,693 | 1.57 |  | 42,648,118 | 1.57 |  | NovaSeq6000 | SRR32328170 |
| Inflorescence stage 2 | Female | 44,021,412 | 1.62 |  | 43,891,162 | 1.61 |  | NovaSeq6000 | SRR32328169 |
| Bud | Female | 29,790,989 | 1.05 |  | 29,708,920 | 1.05 |  | NovaSeq6000 | SRR32328168 |
| Flower | Female | 20,022,247 | 0.69 |  | 19,951,825 | 0.69 |  | NovaSeq6000 | SRR32328167 |
| Leaf | Female | 67,975,326 | 2.40 |  | 67,650,255 | 2.38 |  | NovaSeq6000 | SRR32328166 |
| Stem | Female | 40,637,806 | 1.52 |  | 40,457,258 | 1.51 |  | NovaSeq6000 | SRR32328165 |
| Vegetative shoot apex | - | 34,742,713 | 1.30 |  | 34,638,091 | 1.30 |  | NovaSeq6000 | SRR32328163 |

**Table E. Summary of filtered Oxford Nanopore Technology reads of female *Dioscorea tokoro.***

| Feature | Waka1 (female) | Kita1 (male) |
| --- | --- | --- |
| Number of reads | 2,175,454 | 3,890,192 |
| Total base pairs (Gbp) | 48.5 | 42.6 |
| Genome coverage | 125.1× | 109.8× |
| Average fragment size (bp) | 22,316.5 | 10,943 |
| Longest fragment (bp) | 449,806 | 540,057 |
| Shortest fragment (bp) | 1,000 | 1,000 |
| Fragment N50 (bp) | 37,577 | 27,222 |
| Accession number | SRR36698040 | SRR32328156 |

Genome coverage was estimated based on the genome size of *D. tokoro* (388 Mb).

**Table F. Assembly summary of Oxford Nanopore Technology reads of male and female *Dioscorea tokoro*.**

|  | Contigs | Total number of contigs | Total  base pairs (bp) |  | Contig size (bp) | | | |  | Complete BUSCOs (%) |
| --- | --- | --- | --- | --- | --- | --- | --- | --- | --- | --- |
|  |  |  |  |  | Average | Longest | Shortest | N50 |  |  |
| Step 1 | Waka1 (female) haplotype 1 | 180 | 425,816,851 |  | 2,365,649.2 | 55,928,193 | 1,346 | 19,154,656 |  | 98.3 |
|  | Waka1 (female) haplotype 2 | 326 | 342,705,429 |  | 1,051,243.6 | 8,408,871 | 4,988 | 3,042,110 |  | 81.9 |
|  | Kita1 (male) haplotype 1 | 128 | 415,009,815 |  | 3,242,264.2 | 56,343,901 | 559 | 33,851,599 |  | 98.4 |
|  | Kita1 (male) haplotype 2 | 415 | 300,822,613 |  | 724,873.8 | 5,182,372 | 17,347 | 1,379,312 |  | 76.6 |
| Step 2 | Waka1 (female) haplotype 1 | 169 | 425,737,164 |  | 2,519,154.8 | 55,969,189 | 2,113 | 19,163,377 |  | 98.2 |
|  | Waka1 (female) haplotype 2 | 321 | 342,867,201 |  | 1,068,122.1 | 8,416,596 | 4,989 | 3,042,742 |  | 81.9 |
|  | Kita1 (male) haplotype 1 | 120 | 414,981,487 |  | 3,458,179.1 | 56,355,674 | 5,090 | 33,873,162 |  | 98.4 |
|  | Kita1 (male) haplotype 2 | 414 | 300,776,275 |  | 726,512.7 | 5,183,242 | 17,360 | 1,380,047 |  | 76.6 |
| Step 3 | Waka1 (female) haplotype 1 | 212 | 425,422,506 |  | 2,006,709.9 | 56,002,209 | 113 | 19,172,893 |  | 98.3 |
|  | Waka1 (female) haplotype 2 | 346 | 342,612,608 |  | 990,209.8 | 8,424,838 | 784 | 3,046,925 |  | 81.9 |
|  | Kita1 (male) haplotype 1 | 172 | 414,788,701 |  | 2,411,562.2 | 56,391,428 | 1,051 | 33,885,780 |  | 98.3 |
|  | Kita1 (male) haplotype 2 | 414 | 300,776,275 |  | 726,512.7 | 5,183,242 | 17,360 | 1,380,047 |  | 76.7 |
| Step 4 | Waka1 (female) haplotype 1 | 212 | 425,077,992 |  | 2,005,084.9 | 55,940,541 | 113 | 19,155,052 |  | 98.2 |
| First hypo | Waka1 (female) haplotype 2 | 346 | 342,253,952 |  | 989,173.3 | 8,416,815 | 784 | 3,042,827 |  | 81.9 |
|  | Kita1 (male) haplotype 1 | 172 | 414,446,737 |  | 2,409,574.1 | 56,339,659 | 1,051 | 33,852,229 |  | 98.4 |
|  | Kita1 (male) haplotype 2 | 440 | 300,437,562 |  | 682,812.6 | 5,181,085 | 868 | 1,379,306 |  | 76.7 |
| Step 4 | Waka1 (female) haplotype 1 | 212 | 425,057,702 |  | 2,004,989.2 | 55,937,547 | 113 | 19,154,004 |  | 98.2 |
| Second hypo | Waka1 (female) haplotype 2 | 346 | 342,229,965 |  | 989,103.9 | 8,416,269 | 784 | 3,042,808 |  | 81.9 |
|  | Kita1 (male) haplotype 1 | 172 | 414,436,429 |  | 2,409,514.10 | 56,338,240 | 1,051 | 33,850,955 |  | 98.0 |
|  | Kita1 (male) haplotype 2 | 440 | 300,420,452 |  | 682,773.80 | 5,180,395 | 868 | 1,379,077 |  | 76.0 |

**Table G. Number of anchored contigs and telomere repeats.**

| Reference | Chromosome | Length (bp) | Number of |  | Telomere |  |  |
| --- | --- | --- | --- | --- | --- | --- | --- |
|  |  |  | anchored contigs |  | Side(s) containing telomeres | Number of | Number of |
|  |  |  |  |  |  | repeats on left | repeats on right |
|  |  |  |  |  |  | side | side |
| Waka1 (female) | Chr1 | 55,937,547 | 1 |  | Both | 729 | 718 |
| haplotype 1 | Chr2 | 37,134,381 | 3 |  | Both | 878 | 1,094 |
|  | Chr3 | 49,482,640 | 3 |  | Both | 733 | 1,499 |
|  | Chr4 | 41,477,714 | 2 |  | Left | 1,012 | 0 |
|  | Chr5 | 33,346,315 | 1 |  | Both | 650 | 950 |
|  | Chr6 | 34,110,054 | 2 |  | Both | 753 | 719 |
|  | Chr7 | 31,908,015 | 2 |  | Both | 1,172 | 791 |
|  | Chr8 | 34,957,596 | 2 |  | Both | 887 | 817 |
|  | Chr9 | 47,425,819 | 4 |  | Both | 1,295 | 860 |
|  | Chr10 | 33,199,548 | 4 |  | Both | 815 | 823 |
| Waka1 (female) | Chr1 | 52,437,916 | 15 |  | Neither | 0 | 0 |
| haplotype 2 | Chr2 | 28,334,014 | 21 |  | Neither | 0 | 0 |
|  | Chr3 | 36,432,038 | 26 |  | Right | 0 | 1,543 |
|  | Chr4 | 18,287,848 | 16 |  | Right | 0 | 1,453 |
|  | Chr5 | 28,491,932 | 11 |  | Both | 830 | 788 |
|  | Chr6 | 27,618,455 | 15 |  | Neither | 0 | 0 |
|  | Chr7 | 28,694,074 | 26 |  | Both | 1,219 | 755 |
|  | Chr8 | 24,490,416 | 11 |  | Right | 0 | 1,202 |
|  | Chr9 | 39,839,559 | 16 |  | Right | 0 | 1,945 |
|  | Chr10 | 28,182,205 | 21 |  | Right | 0 | 1,197 |
| Kita1 (male) | Chr1 | 56,338,240 | 1 |  | Both | 946 | 536 |
| haplotype 1 | Chr2 | 37,381,473 | 4 |  | Right | 0 | 737 |
|  | Chr3 | 49,521,298 | 2 |  | Both | 563 | 573 |
|  | Chr4 | 40,747,576 | 1 |  | Both | 692 | 859 |
|  | Chr5 | 33,850,955 | 1 |  | Both | 921 | 858 |
|  | Chr6 | 33,716,633 | 1 |  | Both | 1,256 | 733 |
|  | Chr7 | 30,996,712 | 3 |  | Both | 591 | 721 |
|  | Chr8 | 35,498,219 | 1 |  | Both | 749 | 1,196 |
|  | Chr9 | 47,071,449 | 3 |  | Both | 750 | 668 |
|  | Chr10 | 33,223,422 | 1 |  | Both | 700 | 860 |
| Kita1 (male) | Chr1 | 40,262,596 | 39 |  | Right | 0 | 1,057 |
| haplotype 2 | Chr2 | 30,550,755 | 24 |  | Left | 462 | 0 |
|  | Chr3 | 35,038,069 | 33 |  | Left | 755 | 0 |
|  | Chr4 | 27,502,088 | 26 |  | Left | 1,143 | 0 |
|  | Chr5 | 27,344,658 | 30 |  | Left | 761 | 0 |
|  | Chr6 | 27,862,551 | 22 |  | Neither | 0 | 0 |
|  | Chr7 | 24,808,377 | 26 |  | Neither | 0 | 0 |
|  | Chr8 | 19,658,301 | 18 |  | Left | 748 | 0 |
|  | Chr9 | 26,170,306 | 33 |  | Both | 748 | 695 |
|  | Chr10 | 11,064,507 | 18 |  | Neither | 0 | 0 |

**Table H. Primers used for PCR amplification.**

| Primer | Sequence (5' to 3') |
| --- | --- |
| *BLH9*_F | TCATCAAGCATGGTGACGAGC |
| *BLH9*_R | AGGATATGAAGGCGAGGCAC |
| *HSP90*_F | GGAAACAGAGAGAAAACGG |
| *HSP90*_R | AAACAACAACAACCAGAGCG |
| *Xdh*_F | CCAATTATTGATGCATTCCG |
| *Xdh*_R | CTGTCAATCTAACAGATGCC |

**Table I. TALE superfamily members in *Arabidopsis thaliana* obtained from TAIR.**

| Gene | TAIR Accession | Protein name | Protein length (aa) |
| --- | --- | --- | --- |
| *ATH1* | Locus:2005494 | AT4G32980.1 | 473 |
| *BEL1* | Locus:2177856 | AT5G41410.1 | 611 |
| *BLH1* | Locus:2039250 | AT2G35940.1 | 680 |
| *BLH2* | Locus:2115000 | AT4G36870.1 | 739 |
| *BLH3* | Locus:2018398 | AT1G75410.1 | 524 |
| *BLH4* | Locus:2049035 | AT2G23760.4 | 679 |
| *BLH5* | Locus:2039605 | AT2G27220.2 | 449 |
| *BLH6* | Locus:2139614 | AT4G34610.1 | 532 |
| *BLH7* | Locus:2042609 | AT2G16400.1 | 482 |
| *BLH8* | Locus:2057856 | AT2G27990.1 | 584 |
| *BLH9* | Locus:2185183 | AT5G02030.1 | 575 |
| *BLH10* | Locus:2013154 | AT1G19700.1 | 538 |
| *BLH11* | Locus:2018457 | AT1G75430.1 | 290 |
| *KNATM* | Locus:2006782 | AT1G14760.1 | 142 |
| *STM* | Locus:2027089 | AT1G62360.1 | 382 |
| *KNAT1* | Locus:2128828 | AT4G08150.1 | 398 |
| *KNAT2* | Locus:2026810 | AT1G70510.2 | 314 |
| *KNAT3* | Locus:2146945 | AT5G25220.1 | 431 |
| *KNAT4* | Locus:2184911 | AT5G11060.1 | 393 |
| *KNAT5* | Locus:2116632 | AT4G32040.1 | 383 |
| *KNAT6* | Locus:2028075 | AT1G23380.2 | 329 |
| *KNAT7* | Locus:2015554 | AT1G62990.1 | 291 |

**Table J. HSP90 family members in *Arabidopsis thaliana* obtained from NCBI.**

| Gene | NCBI Accession | Locus name in TAIR | Sequence length (bp) |
| --- | --- | --- | --- |
| HSP90.1 | NM_124642.4 | AT5G52640 | 2,103 |
| HSP90.2 | NM_001203624.1 | AT5G56030 | 2,316 |
| HSP90.3 | NM_124983.4 | AT5G56010 | 2,613 |
| HSP90.4 | NM_124982.3 | AT5G56000 | 2,449 |
| HSP90.5 | NM_126439.4 | AT2G04030 | 2,807 |
| HSP90.6 | NM_111656.4 | AT3G07770 | 3,053 |
| HSP90.7 | NM_118552.4 | AT4G24190 | 3,029 |

**Table K. Primers used for overexpression of *BLH9* and *AtBLH9* in *Arabidopsis thaliana.***

| Primer | Sequence (5' to 3') | Description |
| --- | --- | --- |
| BLH9_BamHI_IF_F | GAGGCCTACGGGGATCCATGTCTTCCGAGGTCGGAGGATA | In-Fusion cloning |
| BLH9_EcoRI_IF_R | CGGGGTACCCGGAATTCTTATTCACCAACACCGAGGCCCAA | In-Fusion cloning |
| AtBLH9_BamHI_IF_F | GAGGCCTACGGGGATCCATGGCTGATGCATACGAGCCTTATC | In-Fusion cloning |
| AtBLH9_EcoRI_IF_R | CGGGGTACCCGGAATTCTCAACCTACAAAATCATGTAGAAACTG | In-Fusion cloning |
| pBICP_35S_F | GATGTGATATCTCCACTGACG | Confirmation of inserted sequence |
| pBICP_35S_R | CTTATCTGGGAACTACTCACA | Confirmation of inserted sequence |
| BLH9_seq_F | GGCGATGCAGGACGTGAATG | Confirmation of inserted sequence |
| AtBLH9_seq_F | GAATGCTATAACGGACCAGC | Confirmation of inserted sequence |
| UBC_qPCR_F | CTGCGACTCAGGGAATCTTCTAA | qPCR |
| UBC_qPCR_R | TTGTGCCATTGAATTGAACCC | qPCR |
| BLH9_qPCR_F | GCTCCTACTTCTCGCTAAACCC | qPCR |
| BLH9_qPCR_R | GGGGAGGGCGTGGATGTGT | qPCR |
| AtBLH9_qPCR_F | CCTAGCTACAGTAATTTCATGGG | qPCR |
| AtBLH9_qPCR_R | CGGCGGCGTTGGCTTCACC | qPCR |

References

1. Tamiru M, Natsume S, Takagi H, White B, Yaegashi H, Shimizu M, et al. Genome sequencing of the staple food crop white Guinea yam enables the development of a molecular marker for sex determination. BMC Biol. 2017;15: 86.

2. De Coster W, Rademakers R. NanoPack2: population-scale evaluation of long-read sequencing data. Bioinformatics. 2022;39. doi:10.1093/bioinformatics/btad311

3. Nie F, Ni P, Huang N, Zhang J, Wang Z, Xiao C, et al. De novo diploid genome assembly using long noisy reads. Nat Commun. 2024;15: 2964.

4. Vaser R, Sović I, Nagarajan N, Šikić M. Fast and accurate de novo genome assembly from long uncorrected reads. Genome Res. 2017;27: 737–746.

5. Kundu R, Casey J, Sung W-K. HyPo: Super Fast & Accurate Polisher for Long Read Genome Assemblies. bioRxiv. 2019. p. 2019.12.19.882506. doi:10.1101/2019.12.19.882506

6. Lo C-C, Chain PSG. Rapid evaluation and quality control of next generation sequencing data with FaQCs. BMC Bioinformatics. 2014;15: 366.

7. Aroney STN, Newell RJP, Nissen JN, Camargo AP, Tyson GW, Woodcroft BJ. CoverM: Read alignment statistics for metagenomics. arXiv [q-bio.GN]. 2025. Available: http://arxiv.org/abs/2501.11217

8. Manni M, Berkeley MR, Seppey M, Simão FA, Zdobnov EM. BUSCO update: Novel and streamlined workflows along with broader and deeper phylogenetic coverage for scoring of eukaryotic, prokaryotic, and viral genomes. Mol Biol Evol. 2021;38: 4647–4654.

9. Ou S, Su W, Liao Y, Chougule K, Agda JRA, Hellinga AJ, et al. Benchmarking transposable element annotation methods for creation of a streamlined, comprehensive pipeline. Genome Biol. 2019;20: 275.

10. Schmieder R, Edwards R. Quality control and preprocessing of metagenomic datasets. Bioinformatics. 2011;27: 863–864.

11. Kim D, Paggi JM, Park C, Bennett C, Salzberg SL. Graph-based genome alignment and genotyping with HISAT2 and HISAT-genotype. Nat Biotechnol. 2019;37: 907–915.

12. Shumate A, Wong B, Pertea G, Pertea M. Improved transcriptome assembly using a hybrid of long and short reads with StringTie. PLoS Comput Biol. 2022;18: e1009730.

13. Haas BJ. TransDecoder. 2025. Available: https://github.com/TransDecoder/TransDecoder

14. Stanke M, Diekhans M, Baertsch R, Haussler D. Using native and syntenically mapped cDNA alignments to improve de novo gene finding. Bioinformatics. 2008;24: 637–644.

15. Pertea G, Pertea M. GFF utilities: GffRead and GffCompare. F1000Res. 2020;9: 304.

16. Sugihara Y, Darkwa K, Yaegashi H, Natsume S, Shimizu M, Abe A, et al. Genome analyses reveal the hybrid origin of the staple crop white Guinea yam (Dioscorea rotundata). Proc Natl Acad Sci U S A. 2020;117: 31987–31992.

17. Li H. Aligning sequence reads, clone sequences and assembly contigs with BWA-MEM. arXiv [q-bio.GN]. 2013. Available: http://arxiv.org/abs/1303.3997

18. Danecek P, Bonfield JK, Liddle J, Marshall J, Ohan V, Pollard MO, et al. Twelve years of SAMtools and BCFtools. Gigascience. 2021;10: giab008.

19. Quinlan AR, Hall IM. BEDTools: a flexible suite of utilities for comparing genomic features. Bioinformatics. 2010;26: 841–842.

20. Wu Y, Bhat PR, Close TJ, Lonardi S. Efficient and accurate construction of genetic linkage maps from the minimum spanning tree of a graph. PLoS Genet. 2008;4: e1000212.

21. Broman KW, Wu H, Sen S, Churchill GA. R/qtl: QTL mapping in experimental crosses. Bioinformatics. 2003;19: 889–890.

22. Taylor J, Butler D. R package ASMap: Efficient genetic linkage map construction and diagnosis. arXiv [stat.CO]. 2017. Available: http://arxiv.org/abs/1705.06916

23. Tang H, Zhang X, Miao C, Zhang J, Ming R, Schnable JC, et al. ALLMAPS: robust scaffold ordering based on multiple maps. Genome Biol. 2015;16: 3.

24. Tang H, Krishnakumar V, Zeng X, Xu Z, Taranto A, Lomas JS, et al. JCVI: A versatile toolkit for comparative genomics analysis. Imeta. 2024;3: e211.

25. Lin Y, Ye C, Li X, Chen Q, Wu Y, Zhang F, et al. quarTeT: a telomere-to-telomere toolkit for gap-free genome assembly and centromeric repeat identification. Hortic Res. 2023;10: uhad127.

26. Cabanettes F, Klopp C. D-GENIES: dot plot large genomes in an interactive, efficient and simple way. PeerJ. 2018;6: e4958.

27. Cheng H, Asri M, Lucas J, Koren S, Li H. Scalable telomere-to-telomere assembly for diploid and polyploid genomes with double graph. Nat Methods. 2024;21: 967–970.

28. Li H. New strategies to improve minimap2 alignment accuracy. Bioinformatics. 2021;37: 4572–4574.

29. Robinson JT, Thorvaldsdottir H, Turner D, Mesirov JP. igv.js: an embeddable JavaScript implementation of the Integrative Genomics Viewer (IGV). Bioinformatics. 2023;39. doi:10.1093/bioinformatics/btac830

30. Käfer J, Lartillot N, Marais GAB, Picard F. Detecting sex-linked genes using genotyped individuals sampled in natural populations. Genetics. 2021;218. doi:10.1093/genetics/iyab053

31. Feron R, Pan Q, Wen M, Imarazene B, Jouanno E, Anderson J, et al. RADSex: A computational workflow to study sex determination using restriction site-associated DNA sequencing data. Mol Ecol Resour. 2021;21: 1715–1731.

32. Akagi T, Segawa T, Uchida R, Tanaka H, Shirasawa K, Yamagishi N, et al. Evolution and functioning of an X-A balance sex-determining system in hops. Nat Plants. 2025. doi:10.1038/s41477-025-02017-6

33. Horiuchi A, Masuda K, Shirasawa K, Onoue N, Fujita N, Ushijima K, et al. Ongoing rapid evolution of a post-Y region revealed by chromosome-scale genome assembly of a hexaploid monoecious persimmon (Diospyros kaki). Mol Biol Evol. 2023;40: msad151.

34. Akagi T, Shirasawa K, Nagasaki H, Hirakawa H, Tao R, Comai L, et al. The persimmon genome reveals clues to the evolution of a lineage-specific sex determination system in plants. PLoS Genet. 2020;16: e1008566.

35. Bredeson JV, Lyons JB, Oniyinde IO, Okereke NR, Kolade O, Nnabue I, et al. Chromosome evolution and the genetic basis of agronomically important traits in greater yam. Nat Commun. 2022;13: 2001.

36. Nakamura T, Yamada KD, Tomii K, Katoh K. Parallelization of MAFFT for large-scale multiple sequence alignments. Bioinformatics. 2018;34: 2490–2492.

37. Suyama M, Torrents D, Bork P. PAL2NAL: robust conversion of protein sequence alignments into the corresponding codon alignments. Nucleic Acids Res. 2006;34: W609-12.

38. Wang D, Zhang Y, Zhang Z, Zhu J, Yu J. KaKs_Calculator 2.0: a toolkit incorporating gamma-series methods and sliding window strategies. Genomics Proteomics Bioinformatics. 2010;8: 77–80.

39. Moraga C, Branco C, Rougemont Q, Jedlička P, Mendoza-Galindo E, Veltsos P, et al. The Silene latifolia genome and its giant Y chromosome. Science. 2025;387: 630–636.

40. Liao Y, Smyth GK, Shi W. The Subread aligner: fast, accurate and scalable read mapping by seed-and-vote. Nucleic Acids Res. 2013;41: e108.

41. Love MI, Huber W, Anders S. Moderated estimation of fold change and dispersion for RNA-seq data with DESeq2. Genome Biol. 2014;15: 550.

42. Viruel J, Forest F, Paun O, Chase MW, Devey D, Couto RS, et al. A nuclear Xdh phylogenetic analysis of yams (Dioscorea: Dioscoreaceae) congruent with plastid trees reveals a new Neotropical lineage. Bot J Linn Soc. 2018;187: 232–246.

43. Hamant O, Pautot V. Plant development: a TALE story. C R Biol. 2010;333: 371–381.

44. Krishna P, Gloor G. The Hsp90 family of proteins in Arabidopsis thaliana. Cell Stress Chaperones. 2001;6: 238–246.

45. Rozewicki J, Li S, Amada KM, Standley DM, Katoh K. MAFFT-DASH: integrated protein sequence and structural alignment. Nucleic Acids Res. 2019;47: W5–W10.

46. Minh BQ, Schmidt HA, Chernomor O, Schrempf D, Woodhams MD, von Haeseler A, et al. IQ-TREE 2: New models and efficient methods for phylogenetic inference in the genomic era. Mol Biol Evol. 2020;37: 1530–1534.

47. Letunic I, Bork P. Interactive Tree of Life (iTOL) v6: recent updates to the phylogenetic tree display and annotation tool. Nucleic Acids Res. 2024;52: W78–W82.

48. Shen W, Le S, Li Y, Hu F. SeqKit: A Cross-Platform and Ultrafast Toolkit for FASTA/Q File Manipulation. PLoS One. 2016;11: e0163962.

49. Kuang Z, Wang Y, Li L, Yang X. miRDeep-P2: accurate and fast analysis of the microRNA transcriptome in plants. Bioinformatics. 2019;35: 2521–2522.

50. Fan K, Wong-Bajracharya J, Lin X, Ni M, Ku Y-S, Li M-W, et al. Differentially expressed microRNAs that target functional genes in mature soybean nodules. Plant Genome. 2021;14: e20103.

51. Camacho C, Coulouris G, Avagyan V, Ma N, Papadopoulos J, Bealer K, et al. BLAST+: architecture and applications. BMC Bioinformatics. 2009;10: 421.

52. Mills DB, Francis WR, Vargas S, Larsen M, Elemans CP, Canfield DE, et al. The last common ancestor of animals lacked the HIF pathway and respired in low-oxygen environments. Elife. 2018;7. doi:10.7554/eLife.31176

53. Langmead B, Trapnell C, Pop M, Salzberg SL. Ultrafast and memory-efficient alignment of short DNA sequences to the human genome. Genome Biol. 2009;10: R25.

54. Mori M, Mise K, Kobayashi K, Okuno T, Furusawa I. Infectivity of plasmids containing brome mosaic virus cDNA linked to the cauliflower mosaic virus 35S RNA promoter. J Gen Virol. 1991;72 ( Pt 2): 243–246.
