# Supplementary figures and images for: Whole-genome sequencing reveals a possible molecular basis of sex determination in the dioecious wild yam Dioscorea tokoro
Source: PLoS Genet. 2026 Apr 20;22(4):e1012123. doi: 10.1371/journal.pgen.1012123 (PMC13128126; doi:10.1371/journal.pgen.1012123)

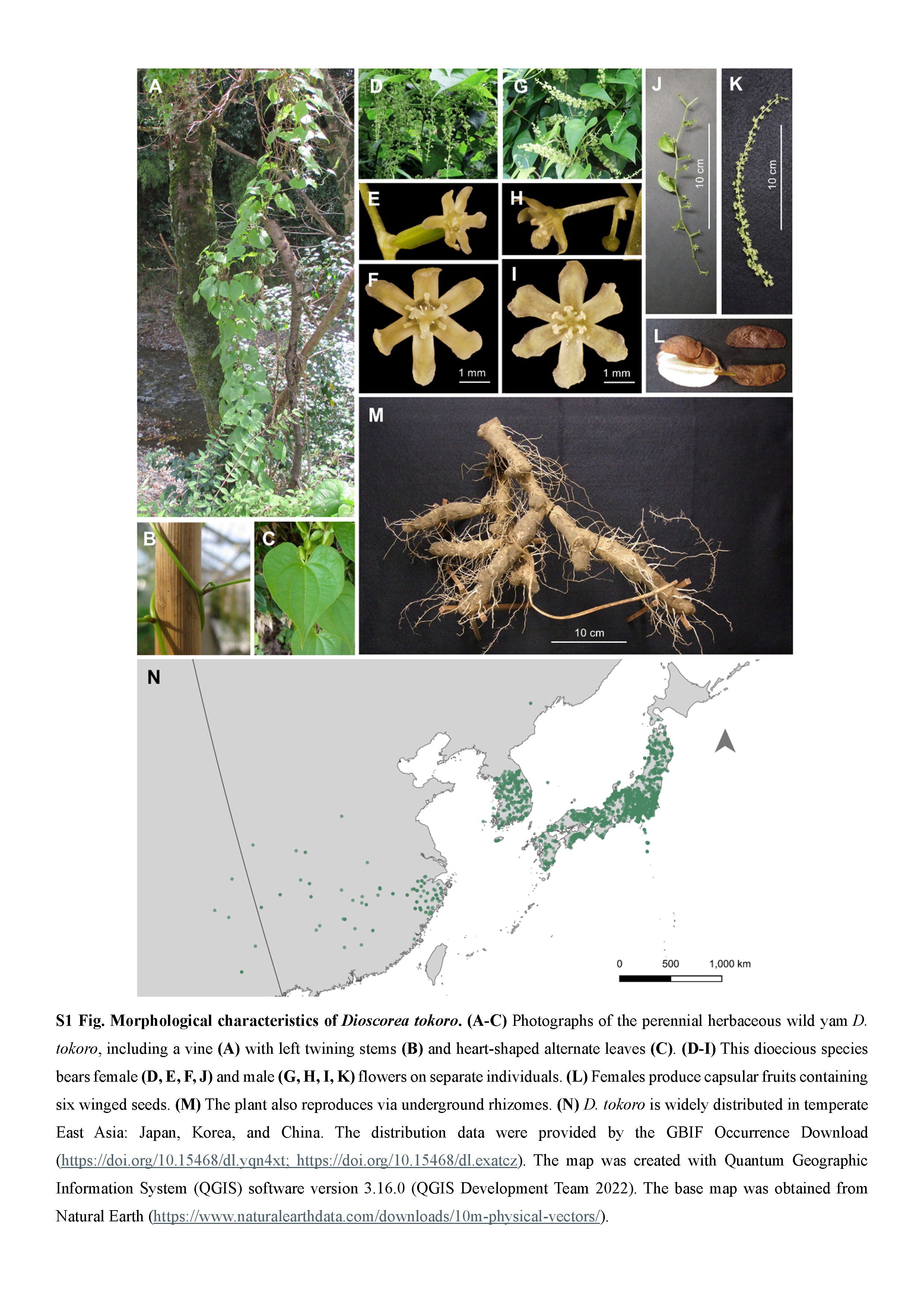

Supplement: S1 Fig — (TIF) [file pgen.1012123.s002.tif]

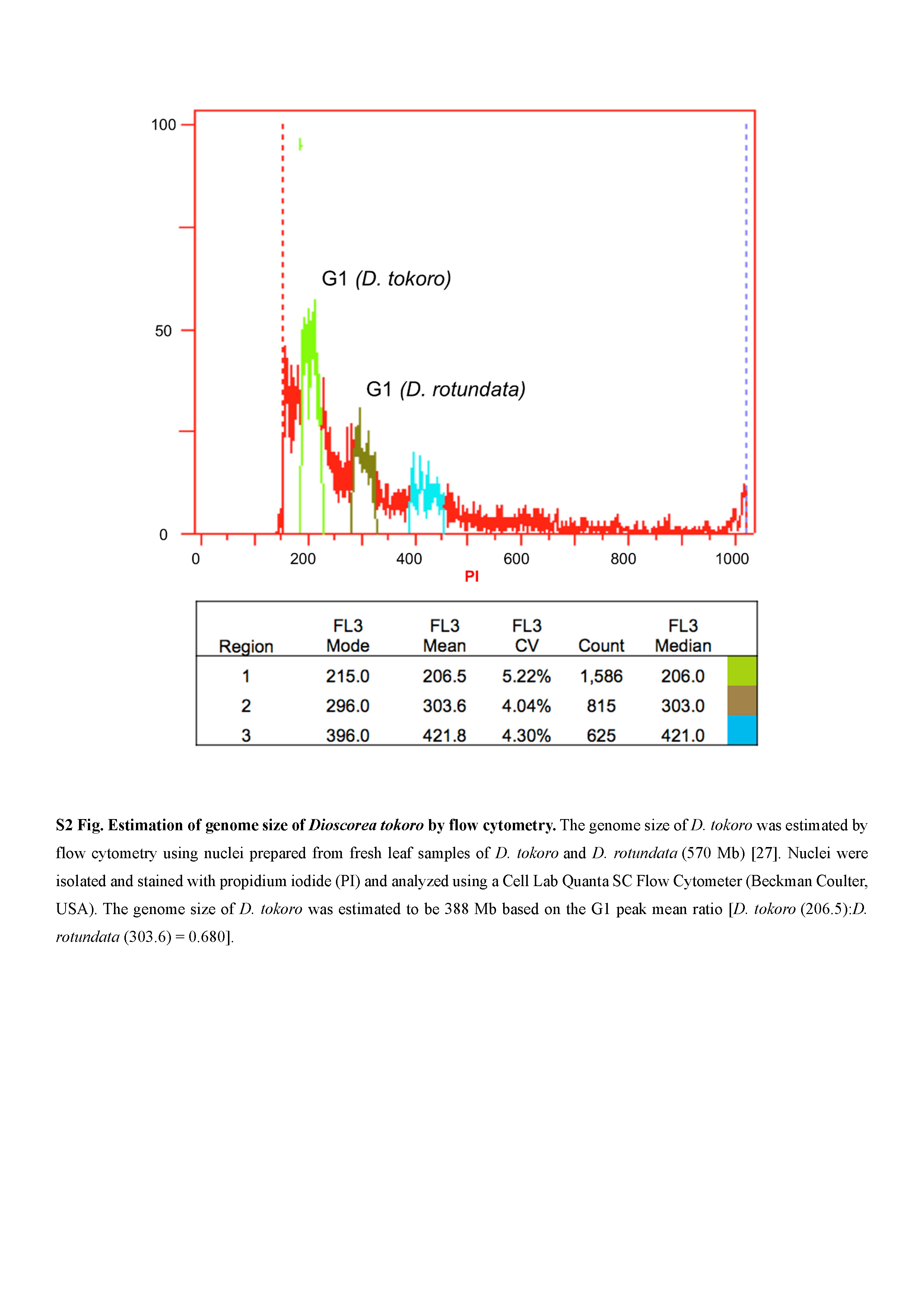

Supplement: S2 Fig — (TIF) [file pgen.1012123.s003.tif]

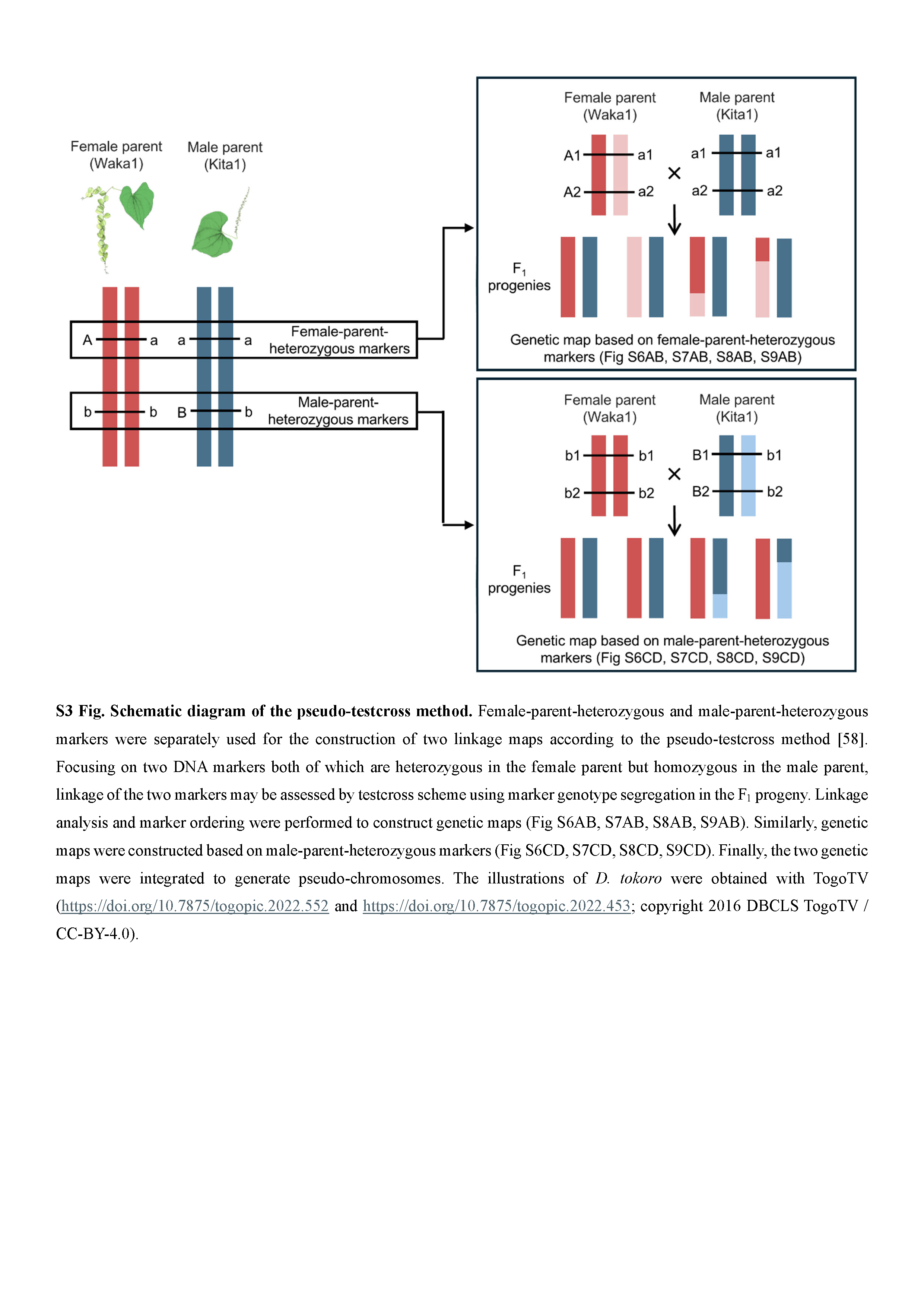

Supplement: S3 Fig — (TIF) [file pgen.1012123.s004.tif]

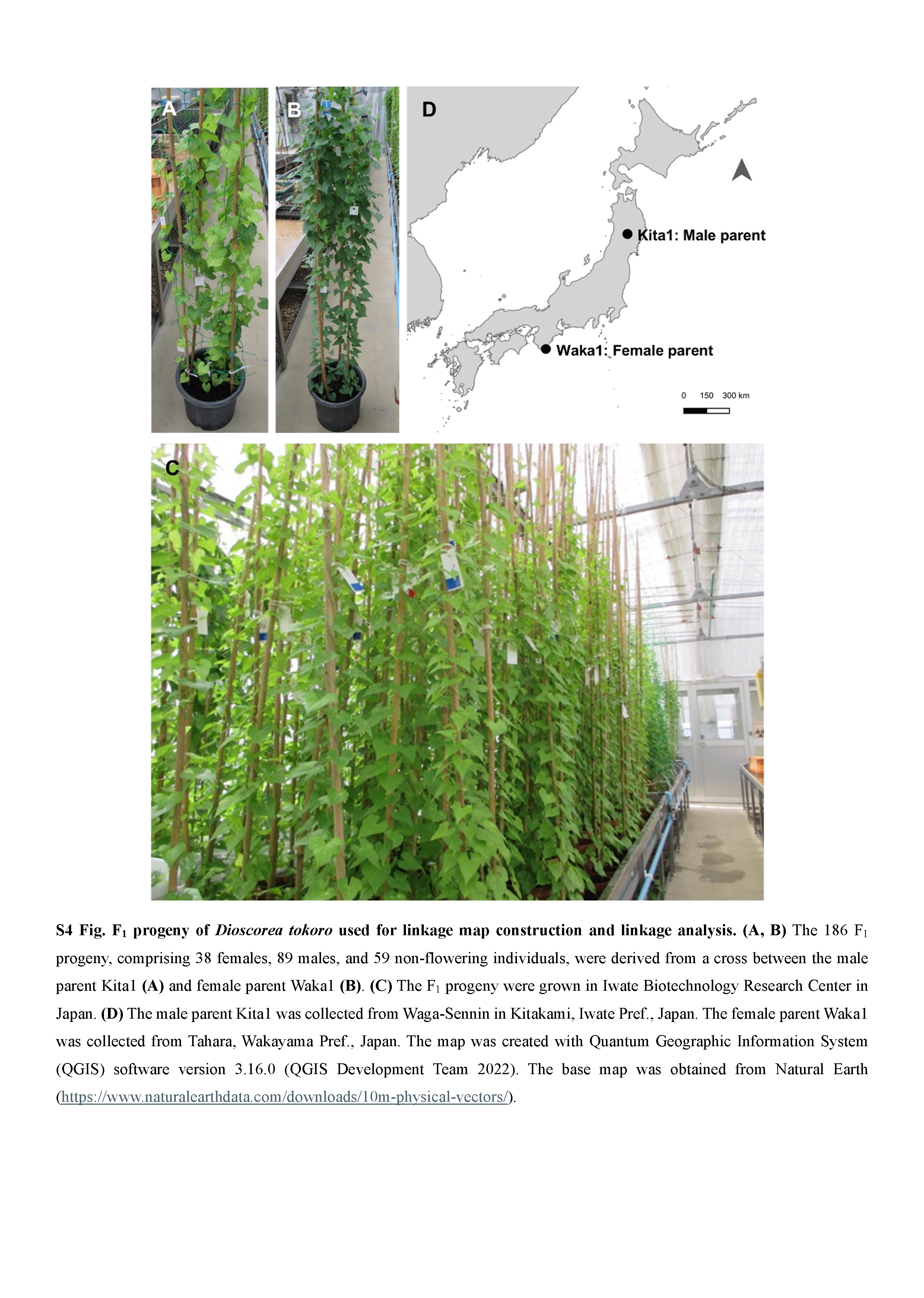

Supplement: S4 Fig — (TIF) [file pgen.1012123.s005.tif]

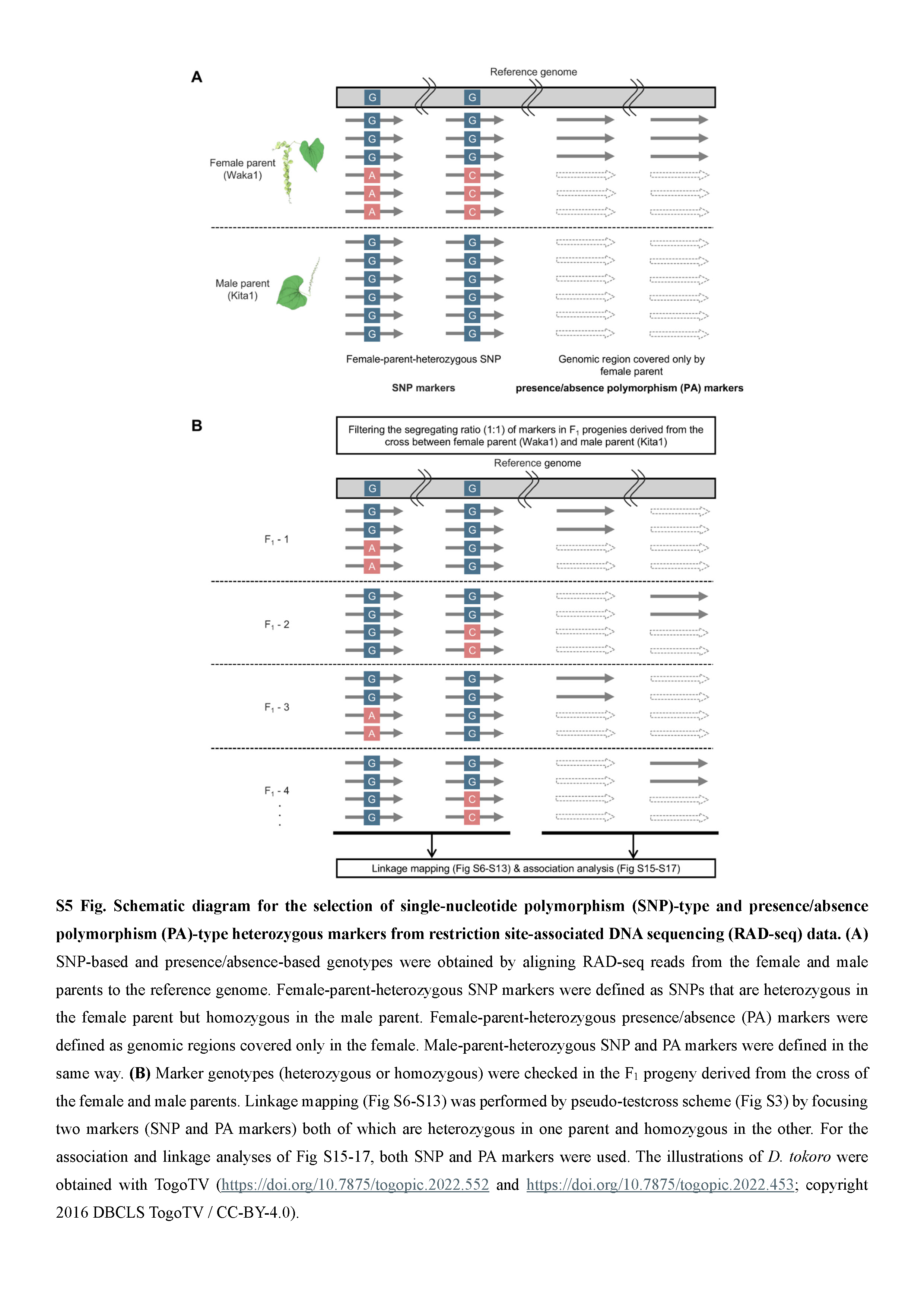

Supplement: S5 Fig — (TIF) [file pgen.1012123.s006.tif]

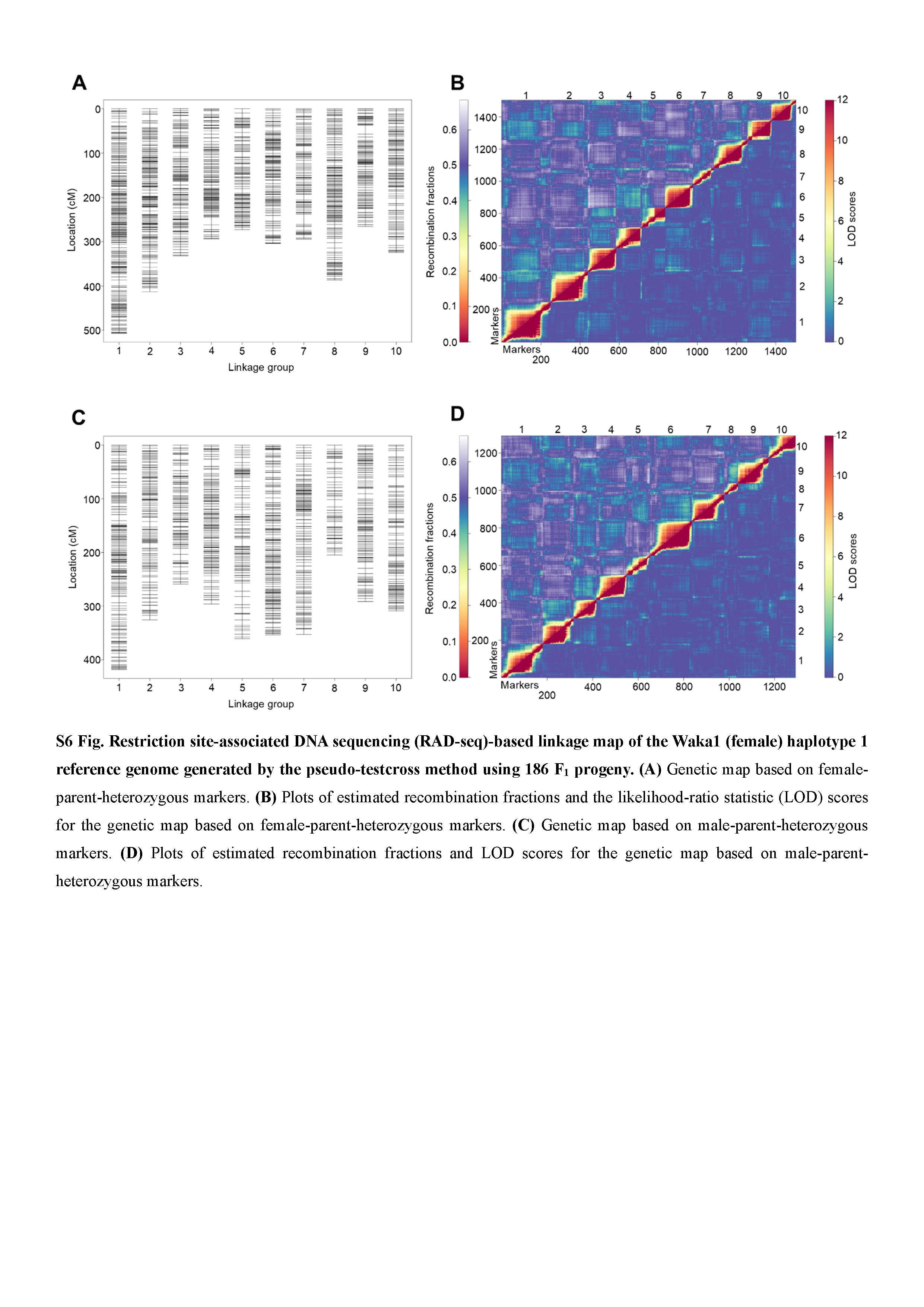

Supplement: S6 Fig — (TIF) [file pgen.1012123.s007.tif]

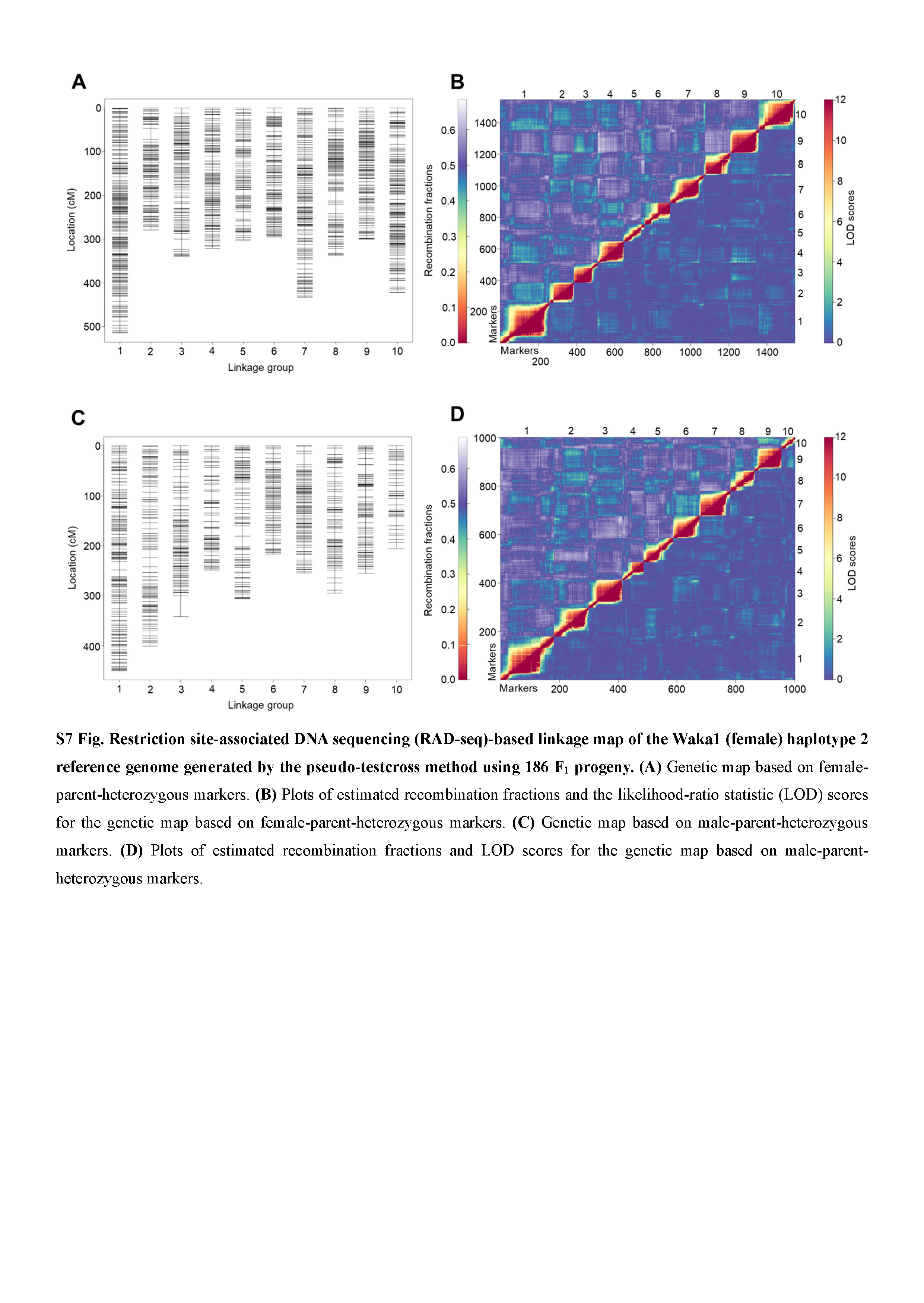

Supplement: S7 Fig — (TIF) [file pgen.1012123.s008.tif]

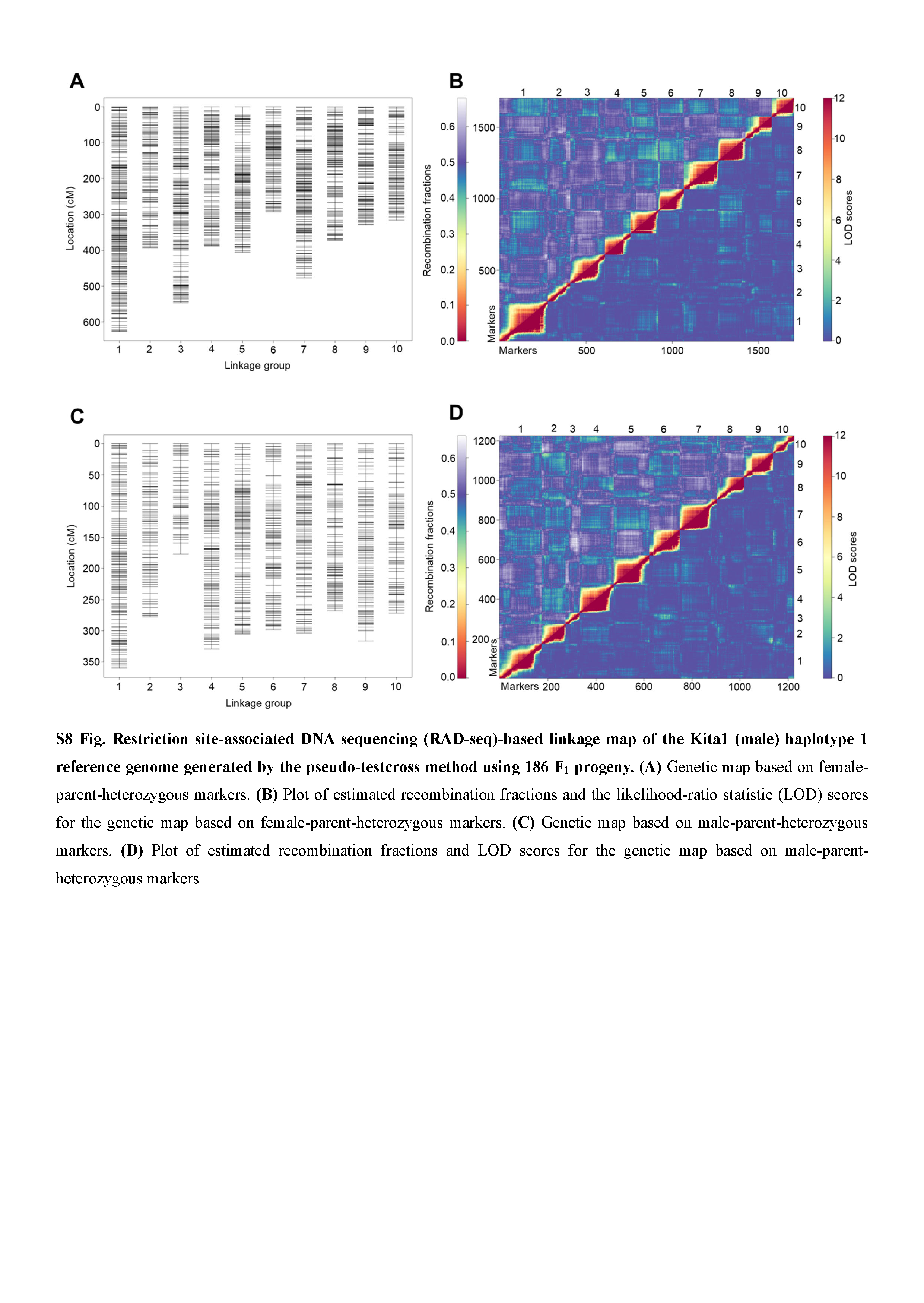

Supplement: S8 Fig — (TIF) [file pgen.1012123.s009.tif]

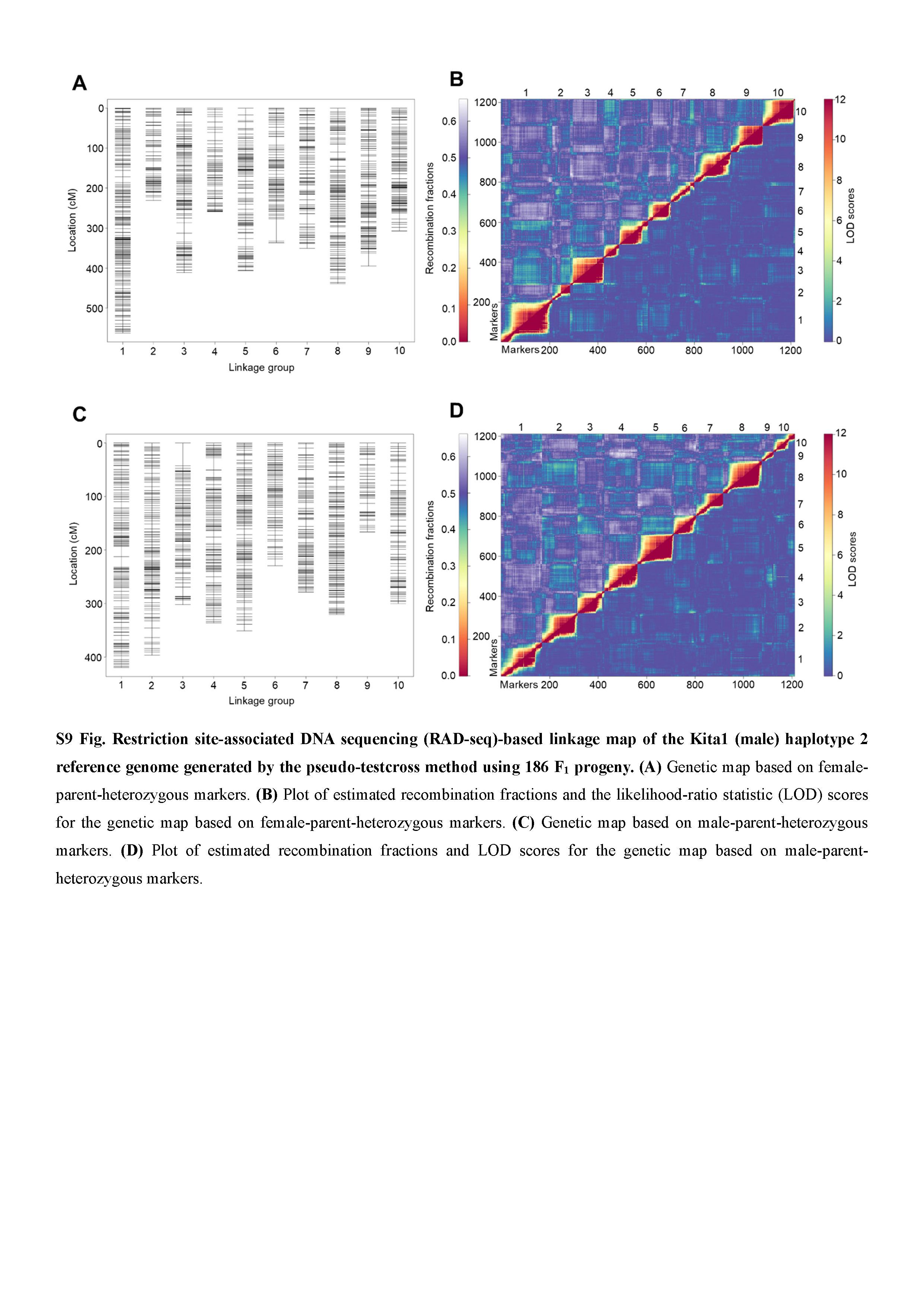

Supplement: S9 Fig — (TIF) [file pgen.1012123.s010.tif]

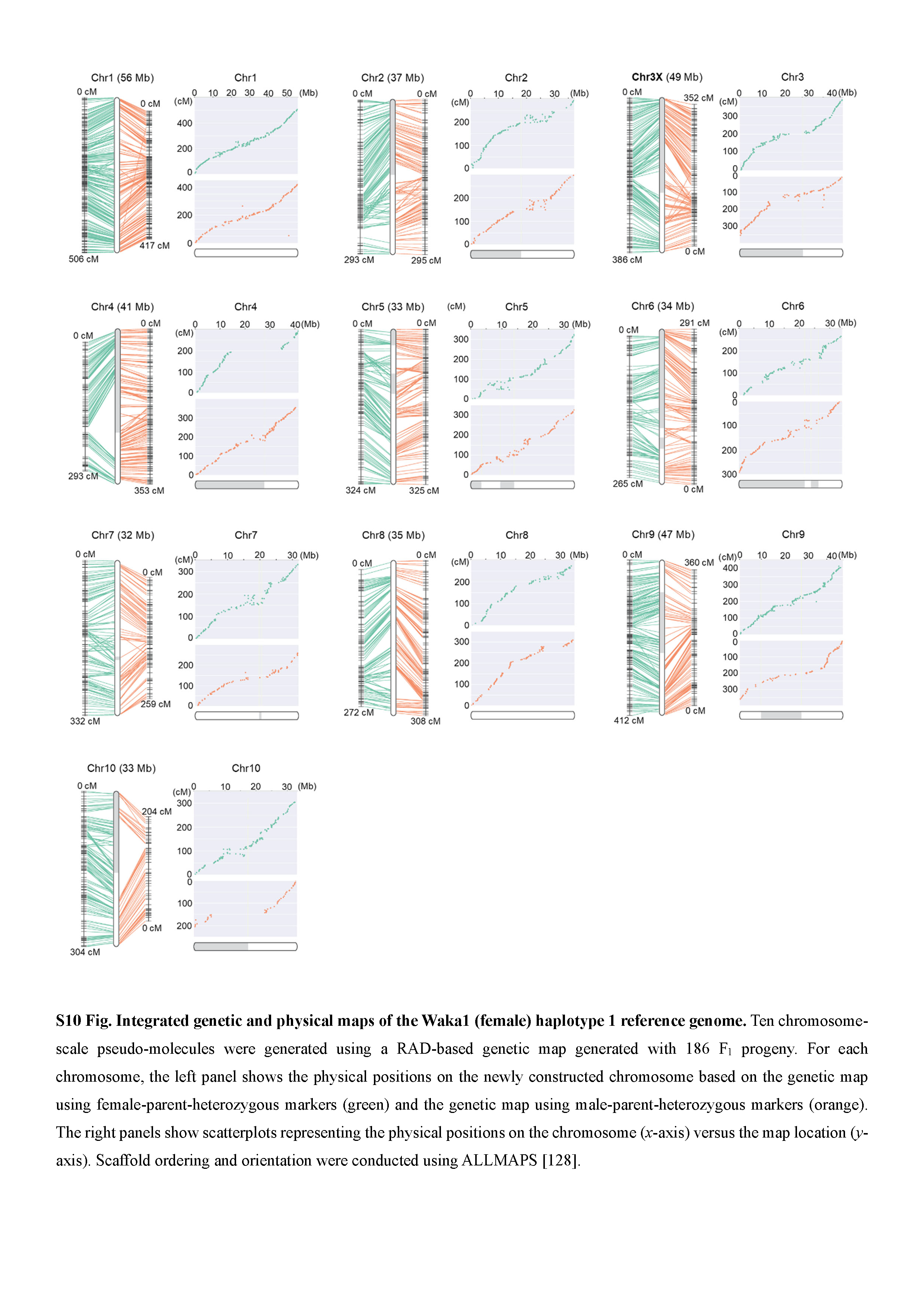

Supplement: S10 Fig — (TIF) [file pgen.1012123.s011.tif]

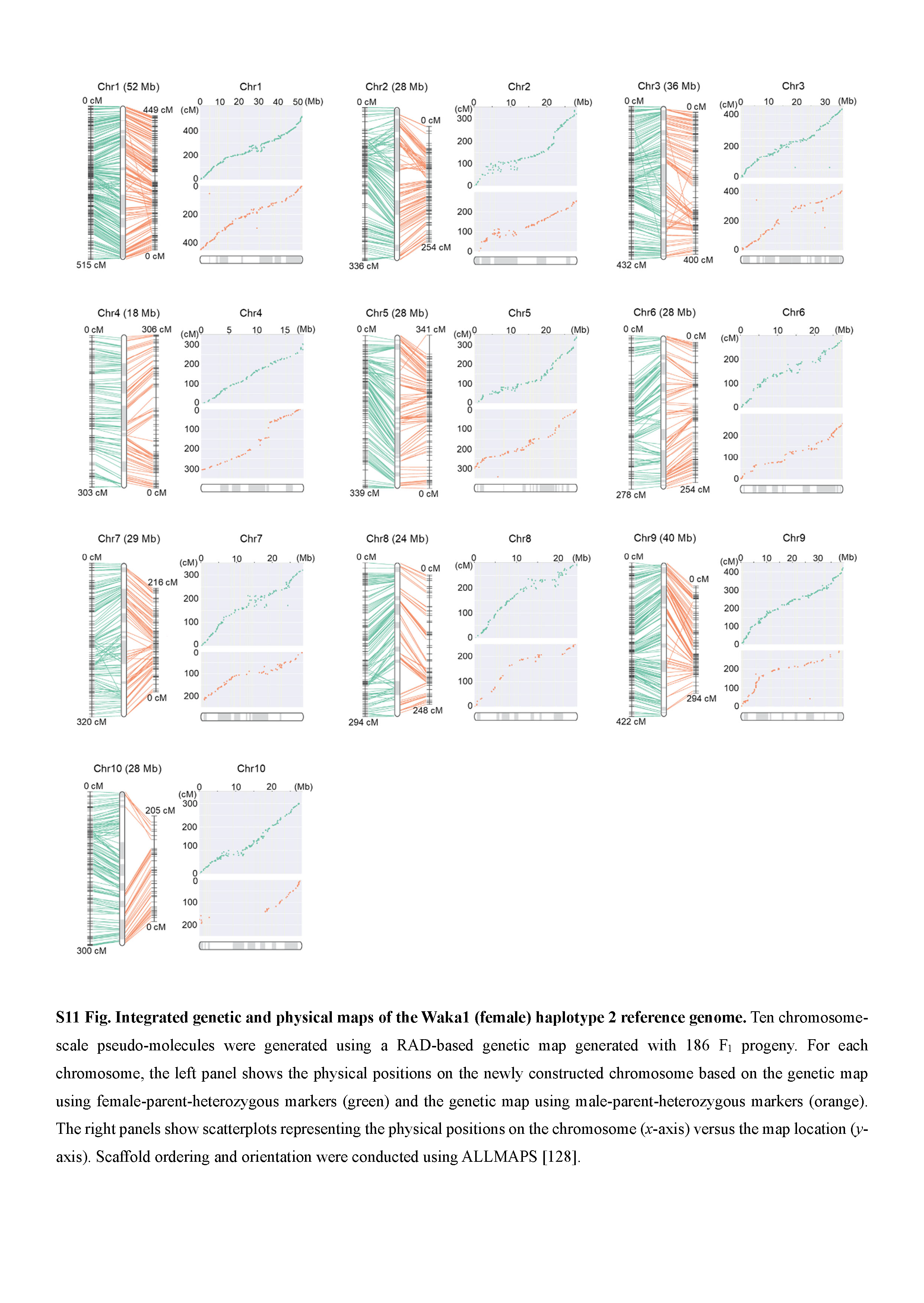

Supplement: S11 Fig — (TIF) [file pgen.1012123.s012.tif]

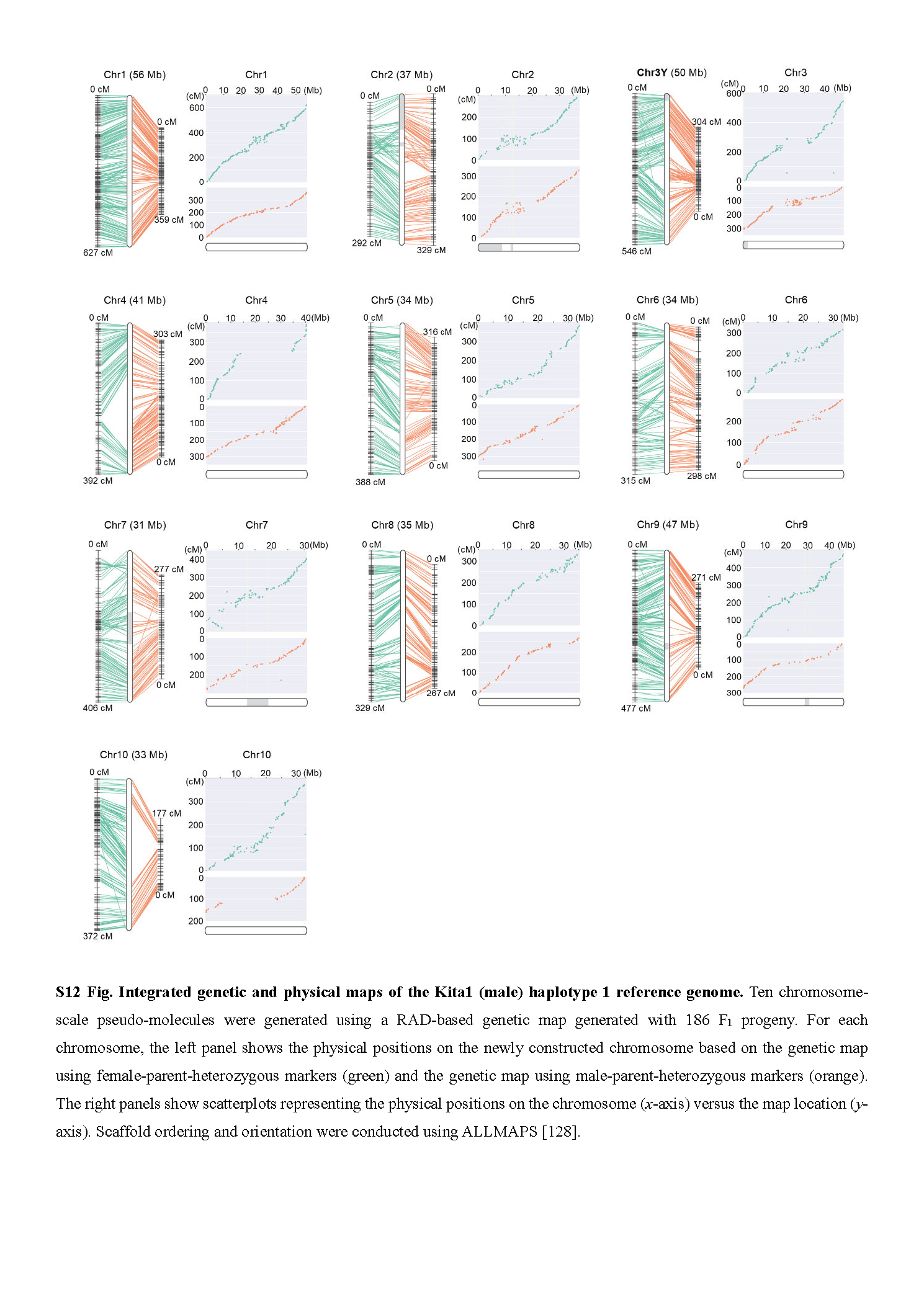

Supplement: S12 Fig — (TIF) [file pgen.1012123.s013.tif]

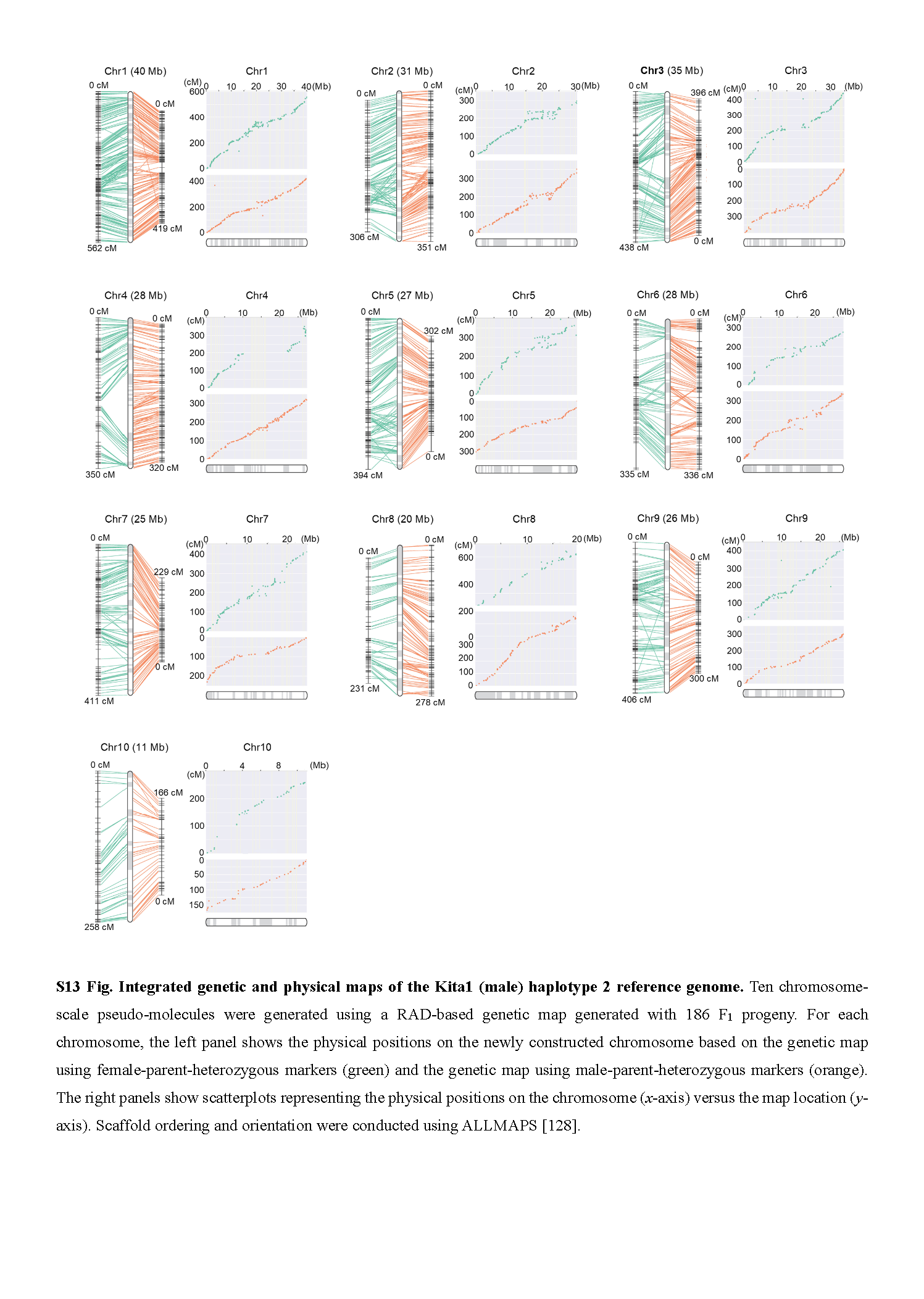

Supplement: S13 Fig — (TIF) [file pgen.1012123.s014.tif]

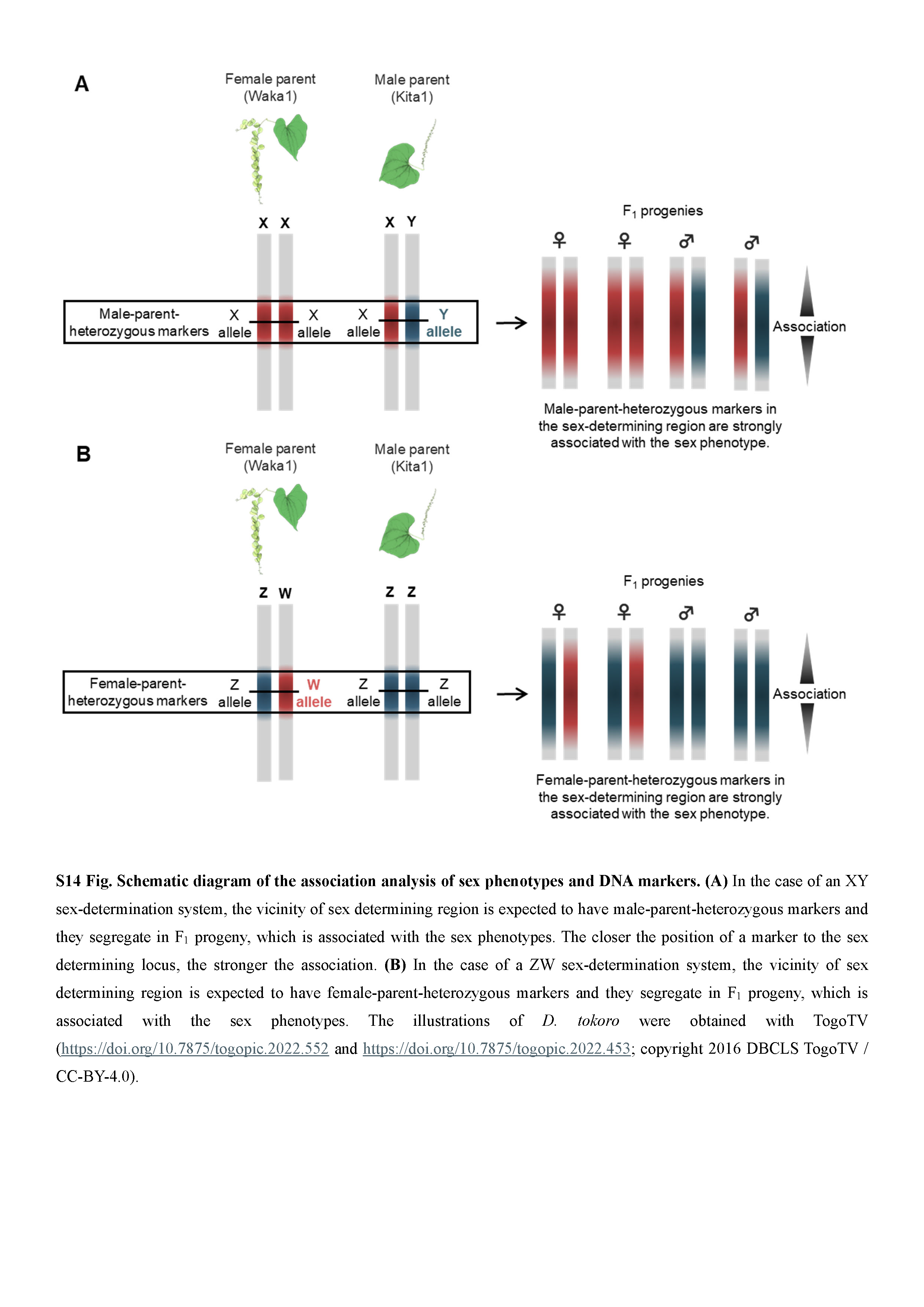

Supplement: S14 Fig — (TIF) [file pgen.1012123.s015.tif]

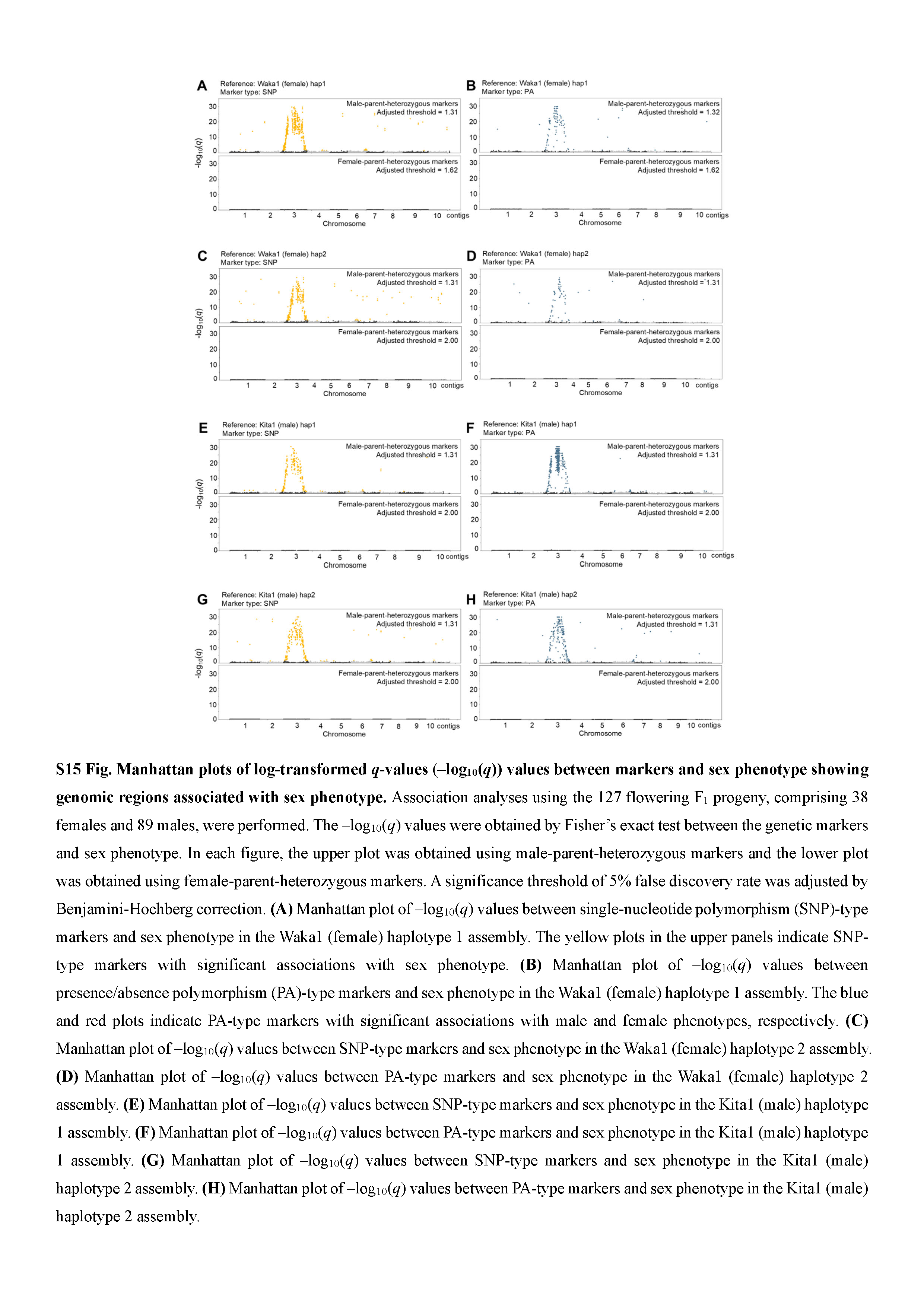

Supplement: S15 Fig — (TIF) [file pgen.1012123.s016.tif]

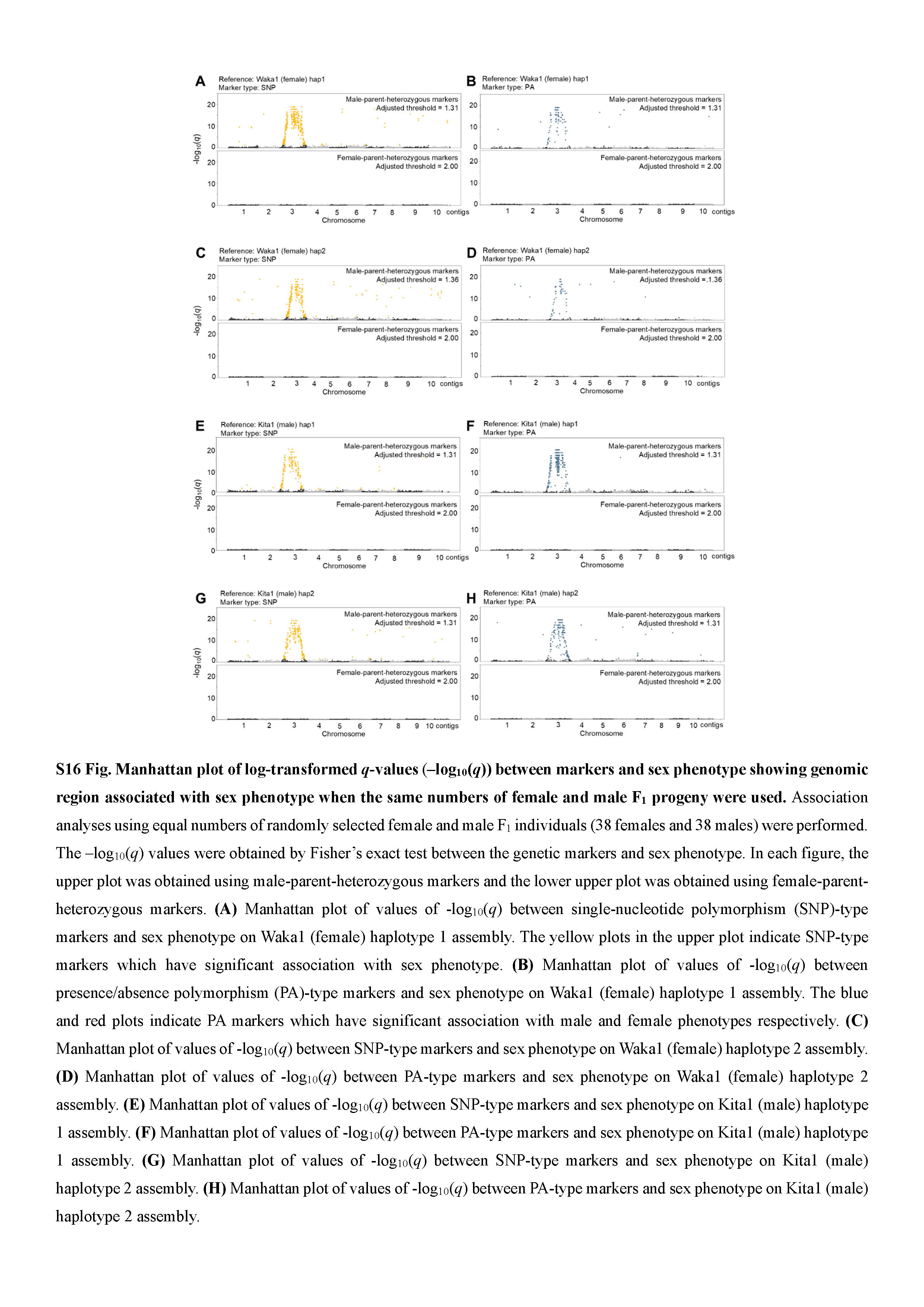

Supplement: S16 Fig — (TIF) [file pgen.1012123.s017.tif]

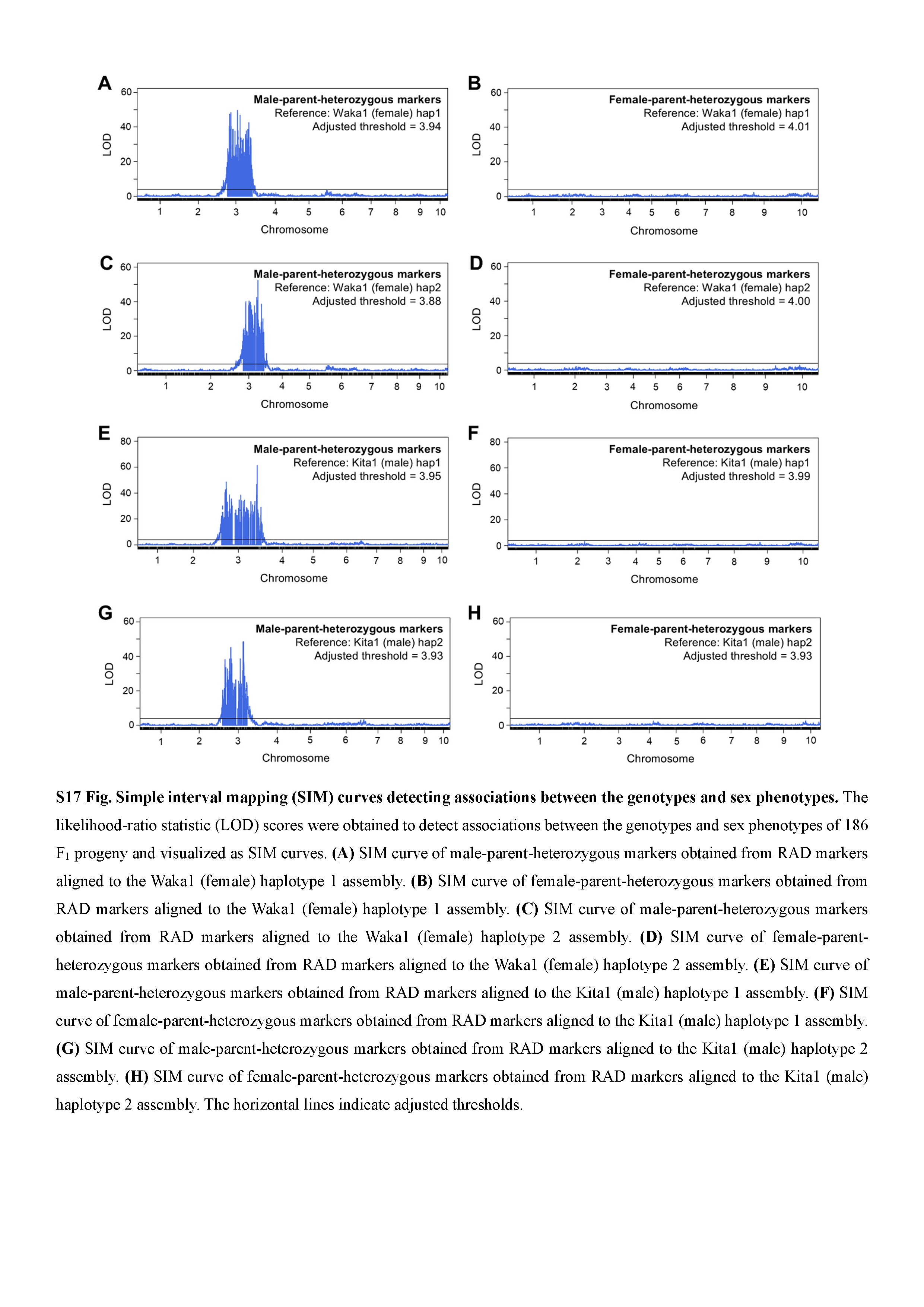

Supplement: S17 Fig — (TIF) [file pgen.1012123.s018.tif]

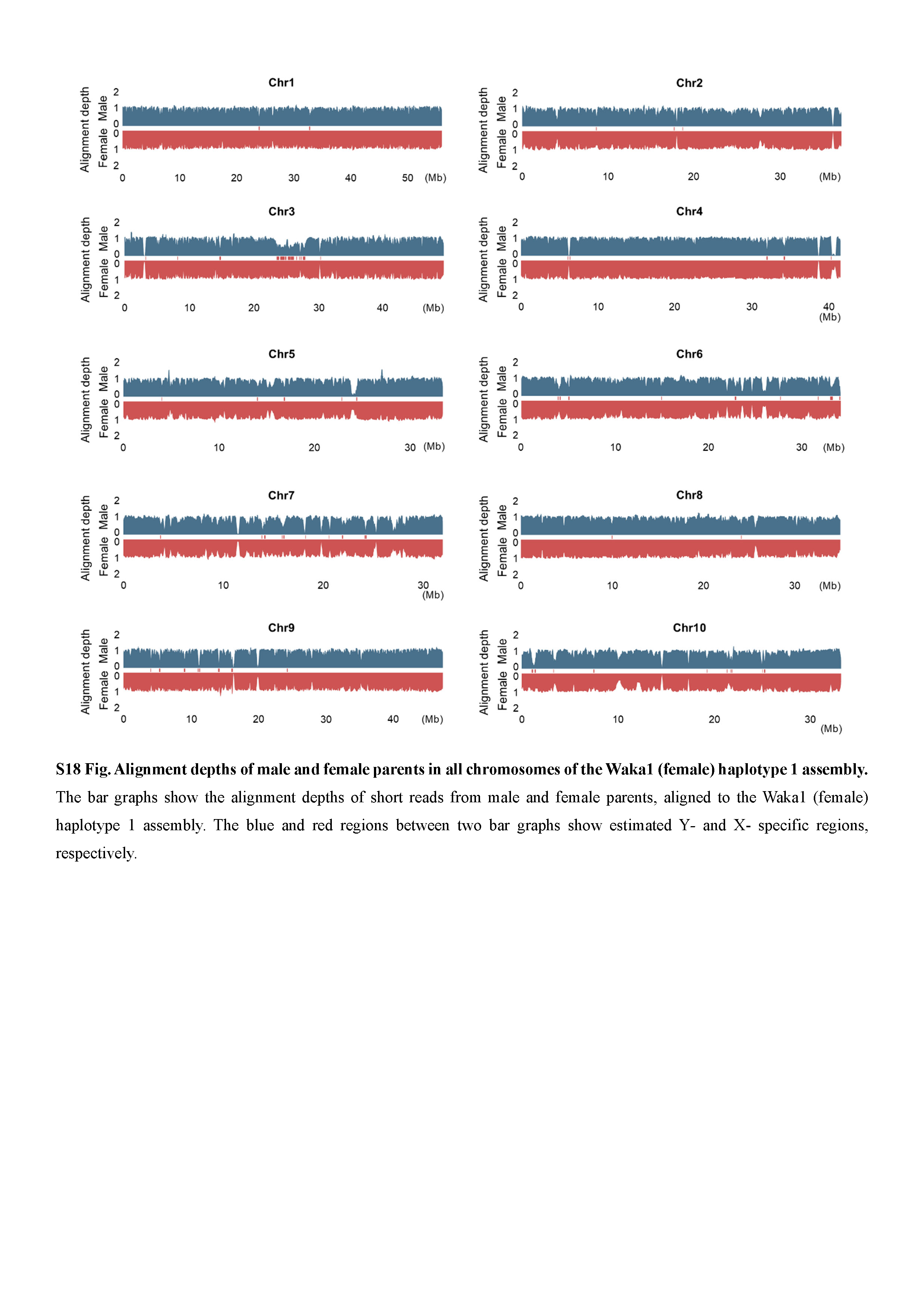

Supplement: S18 Fig — (TIF) [file pgen.1012123.s019.tif]

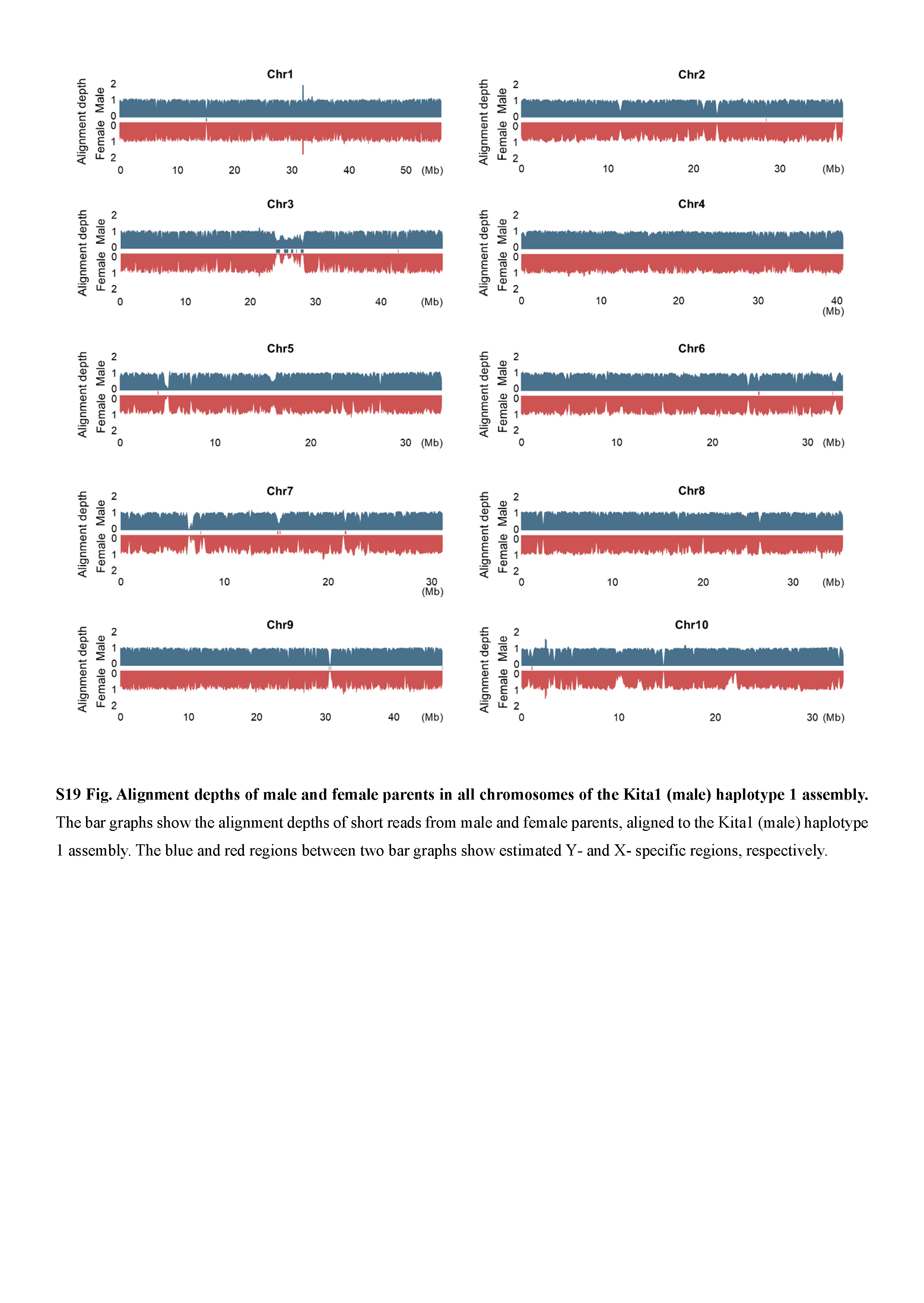

Supplement: S19 Fig — (TIF) [file pgen.1012123.s020.tif]

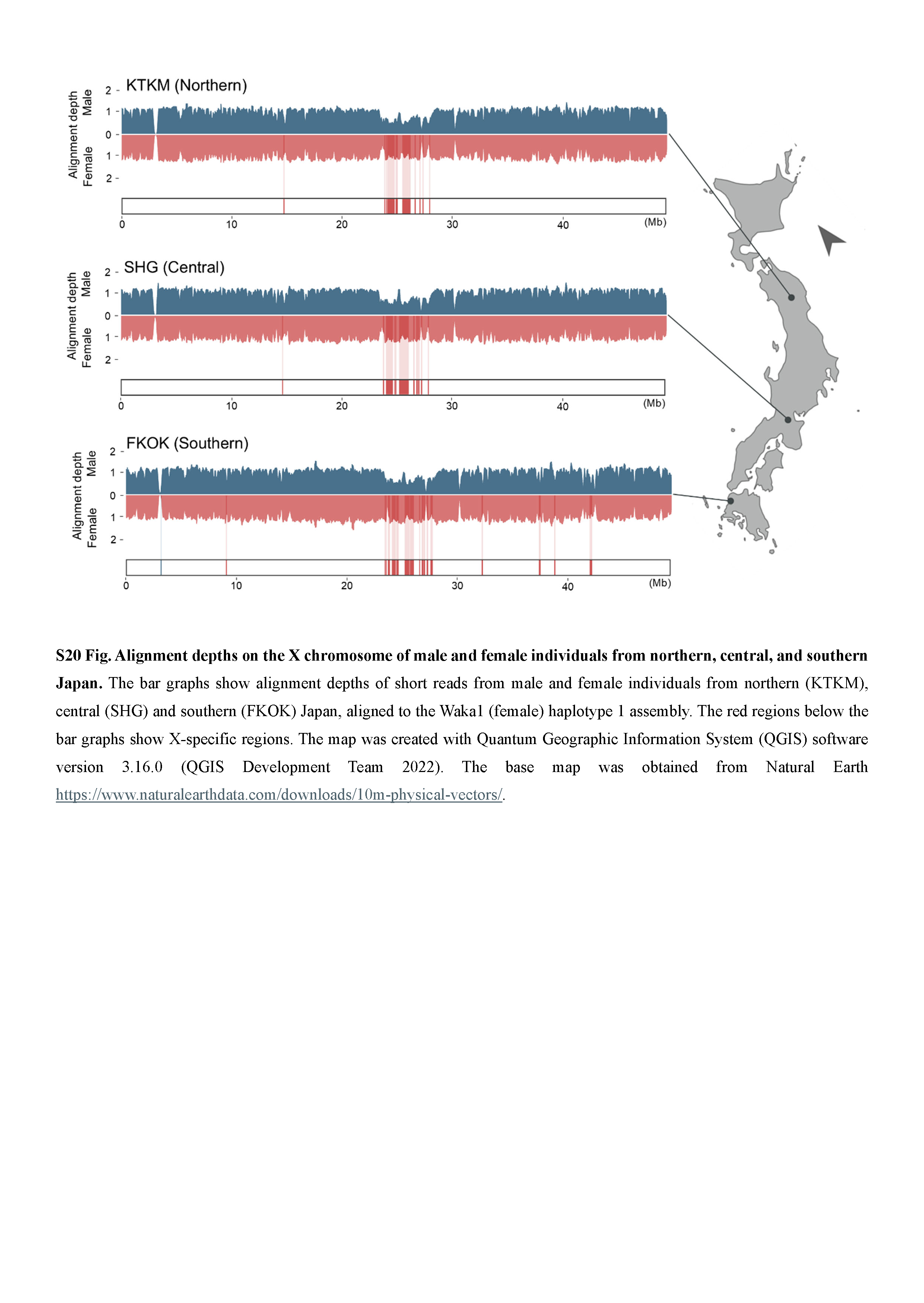

Supplement: S20 Fig — (TIF) [file pgen.1012123.s021.tif]

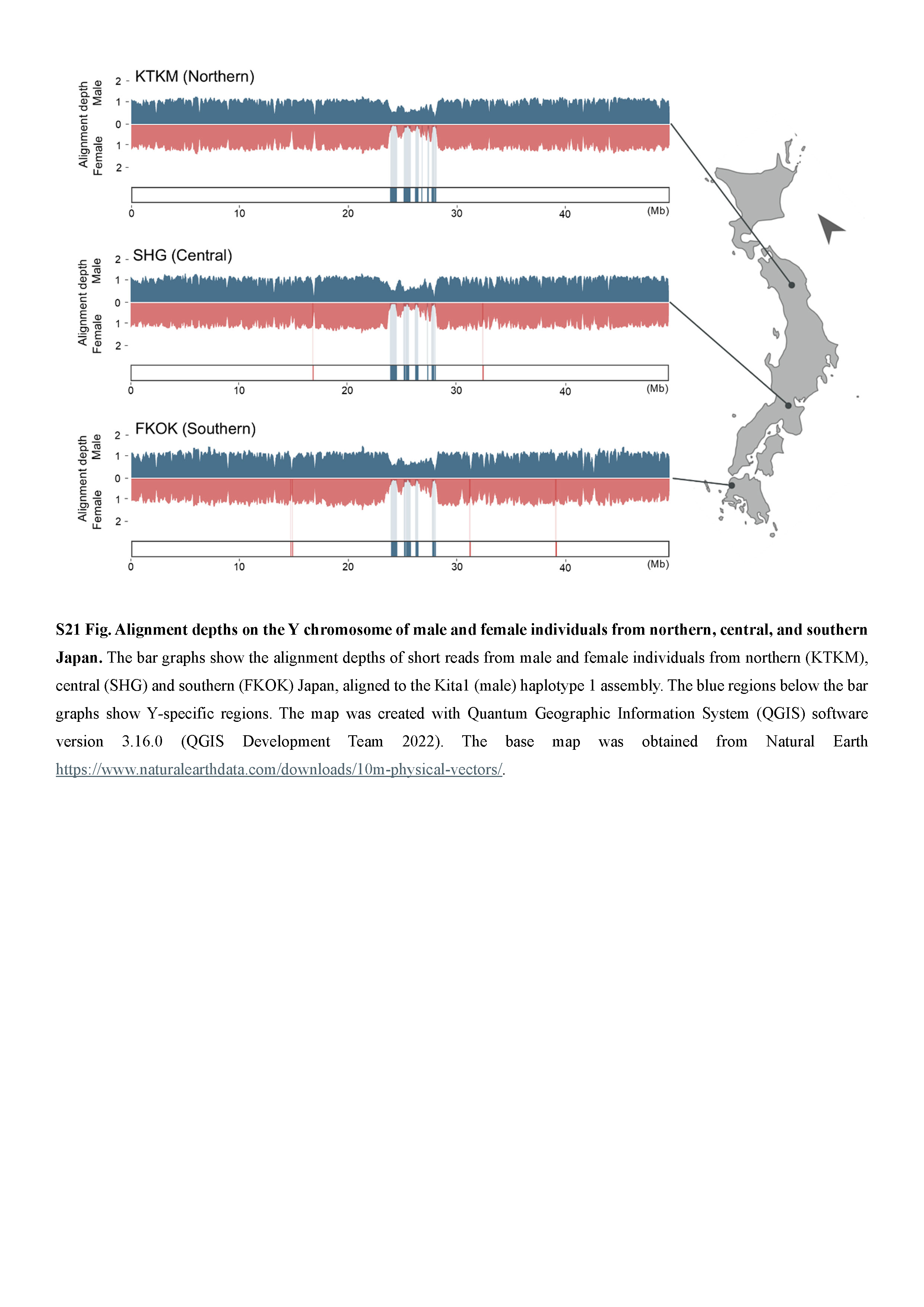

Supplement: S21 Fig — (TIF) [file pgen.1012123.s022.tif]

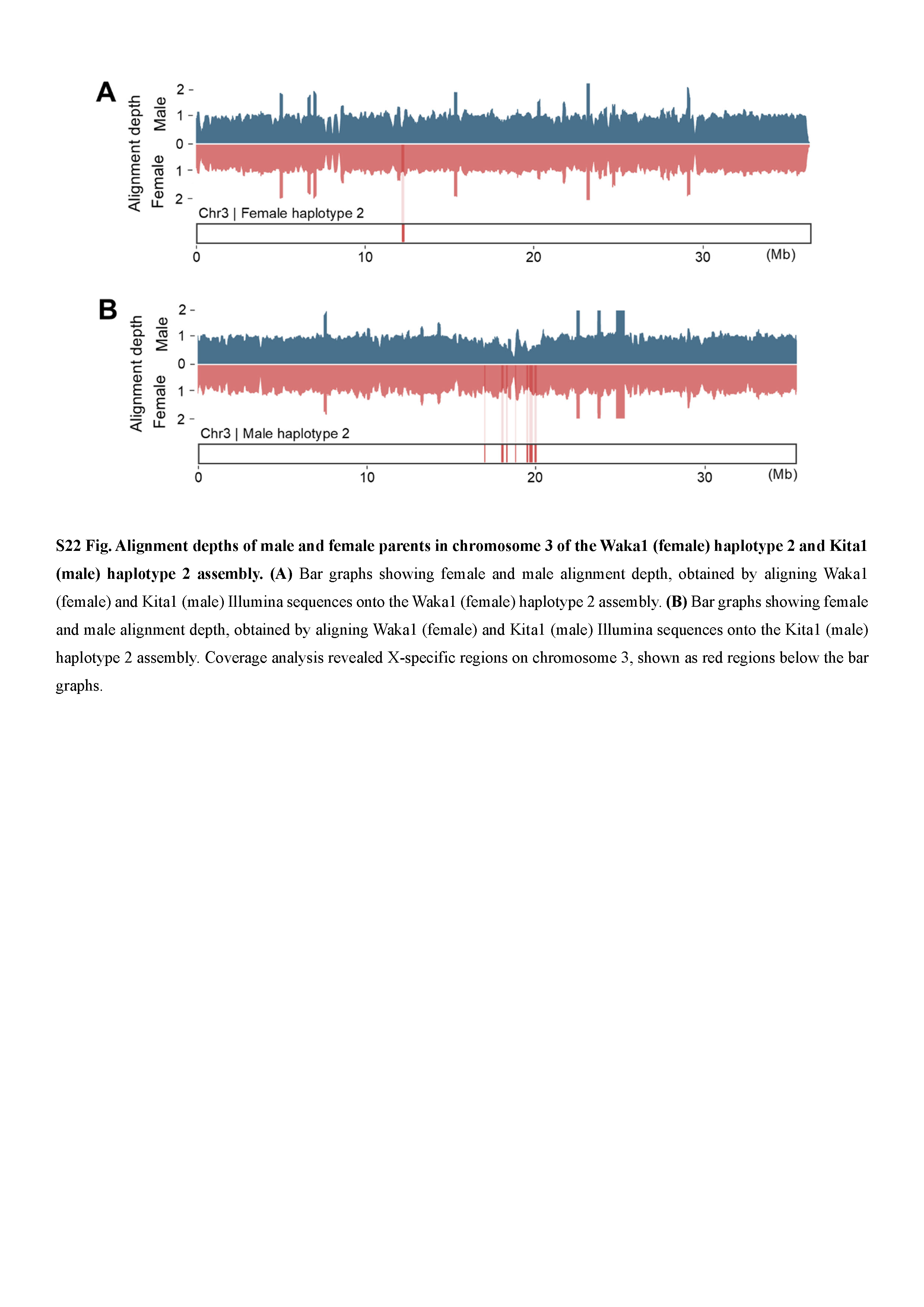

Supplement: S22 Fig — (TIF) [file pgen.1012123.s023.tif]

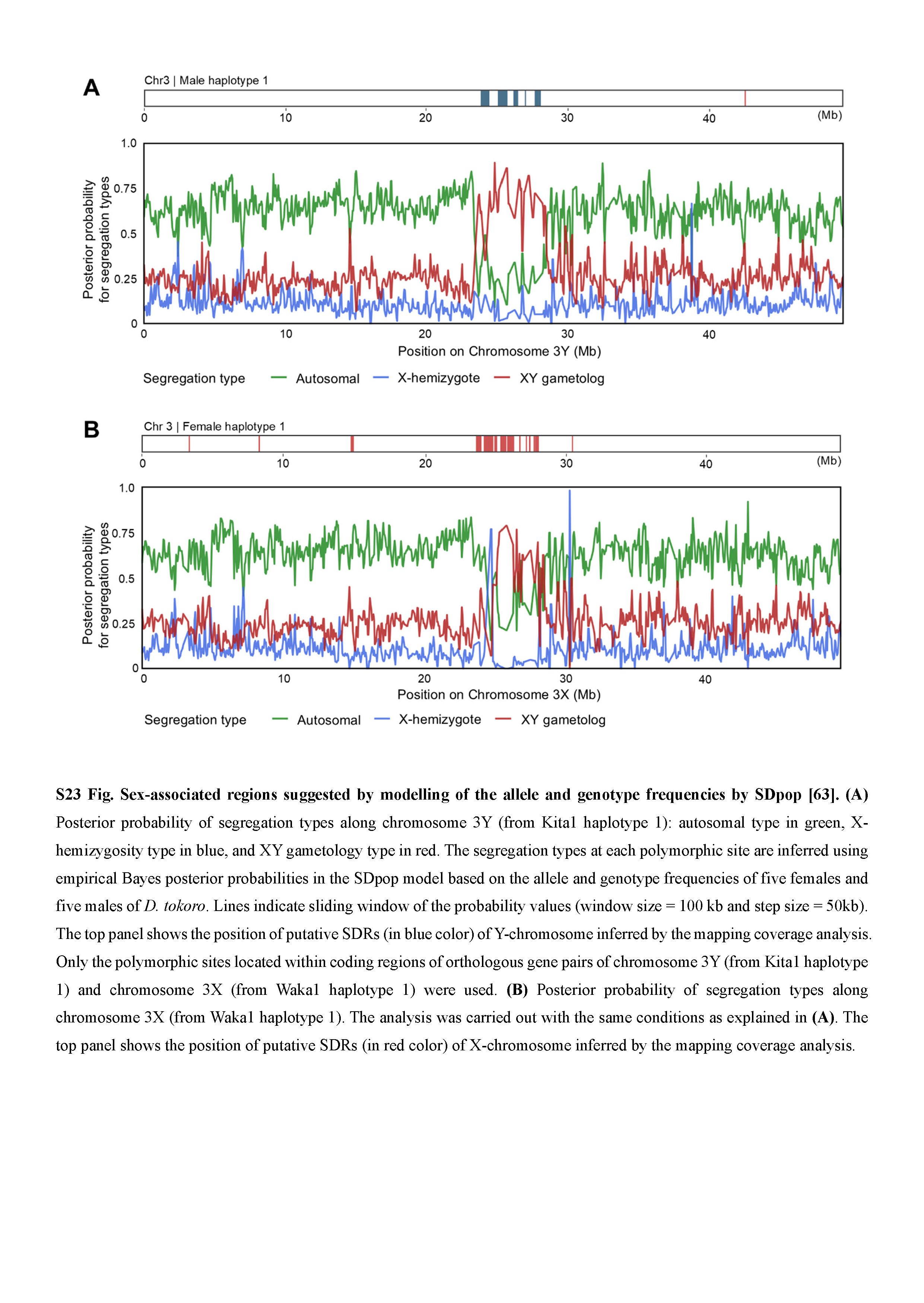

Supplement: S23 Fig — (TIF) [file pgen.1012123.s024.tif]

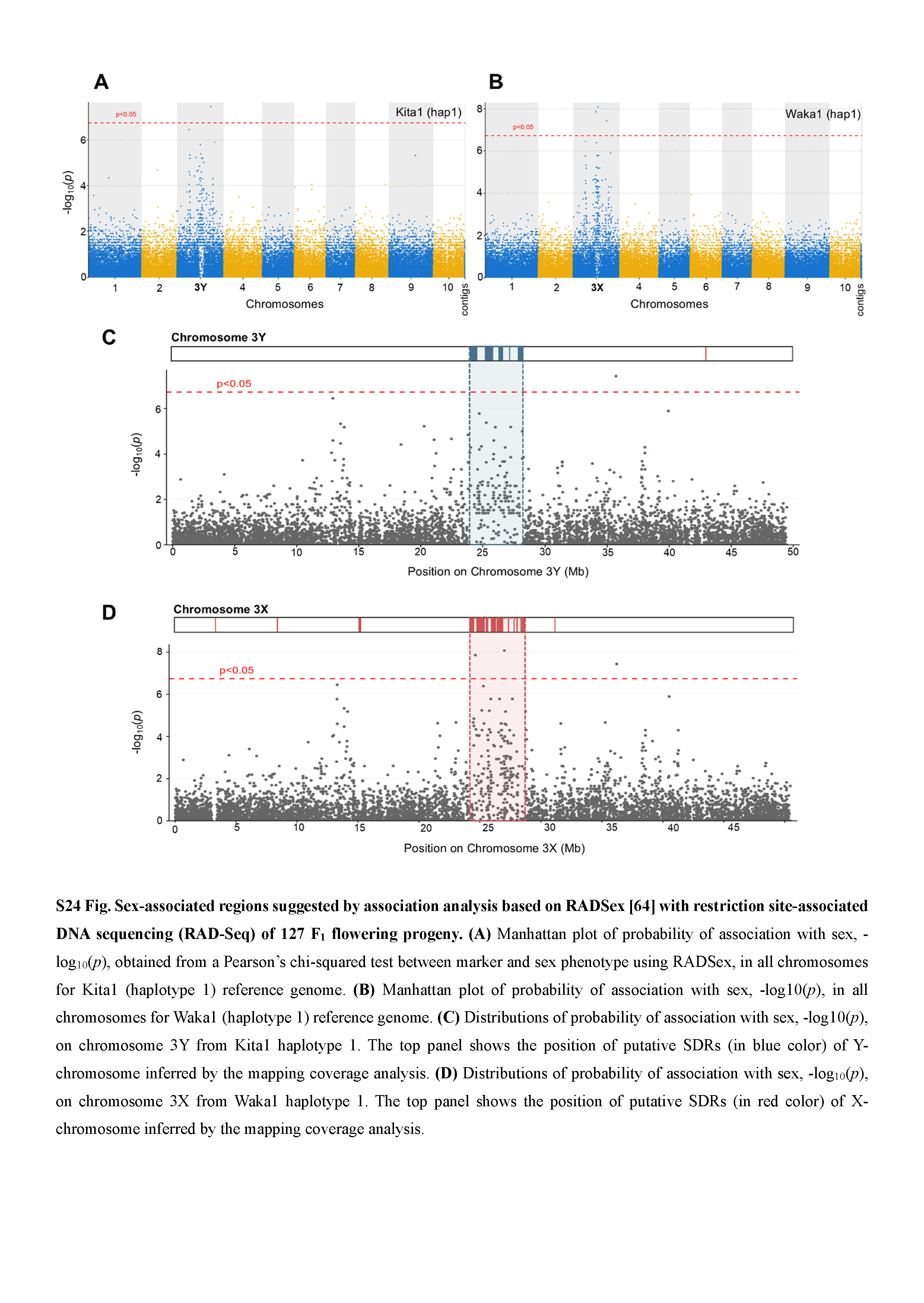

Supplement: S24 Fig — (TIF) [file pgen.1012123.s025.tif]

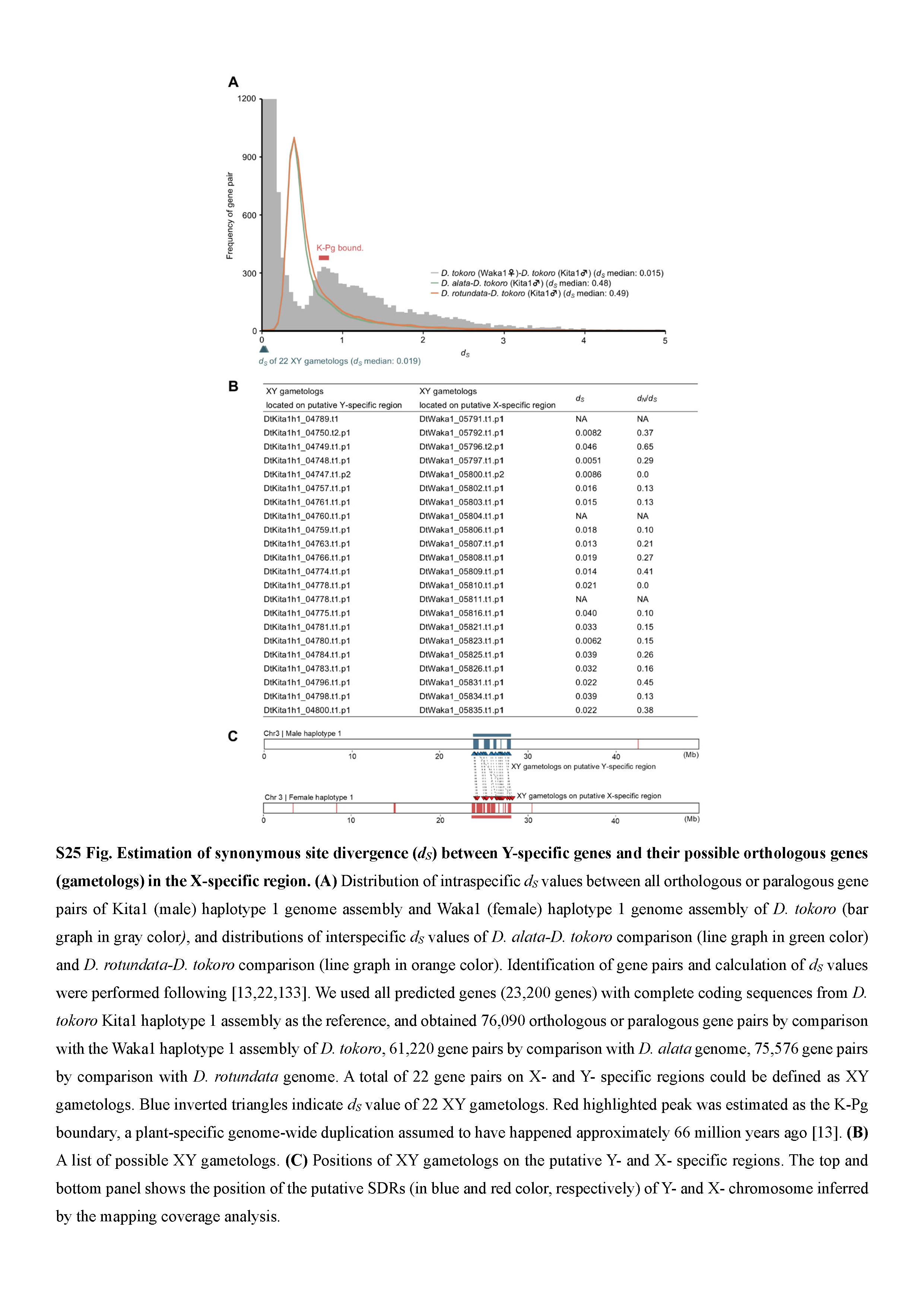

Supplement: S25 Fig — (TIF) [file pgen.1012123.s026.tif]

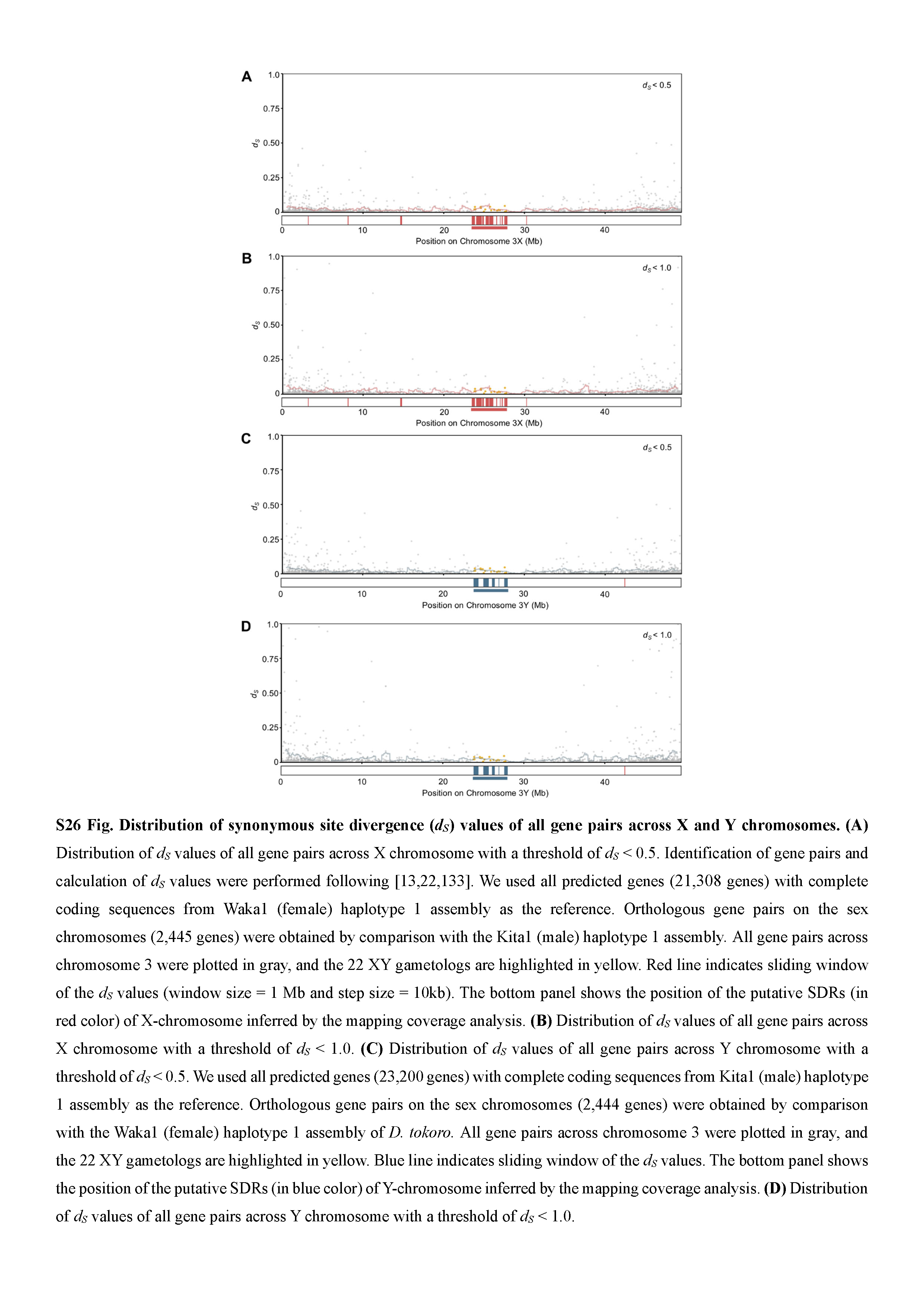

Supplement: S26 Fig — (TIF) [file pgen.1012123.s027.tif]

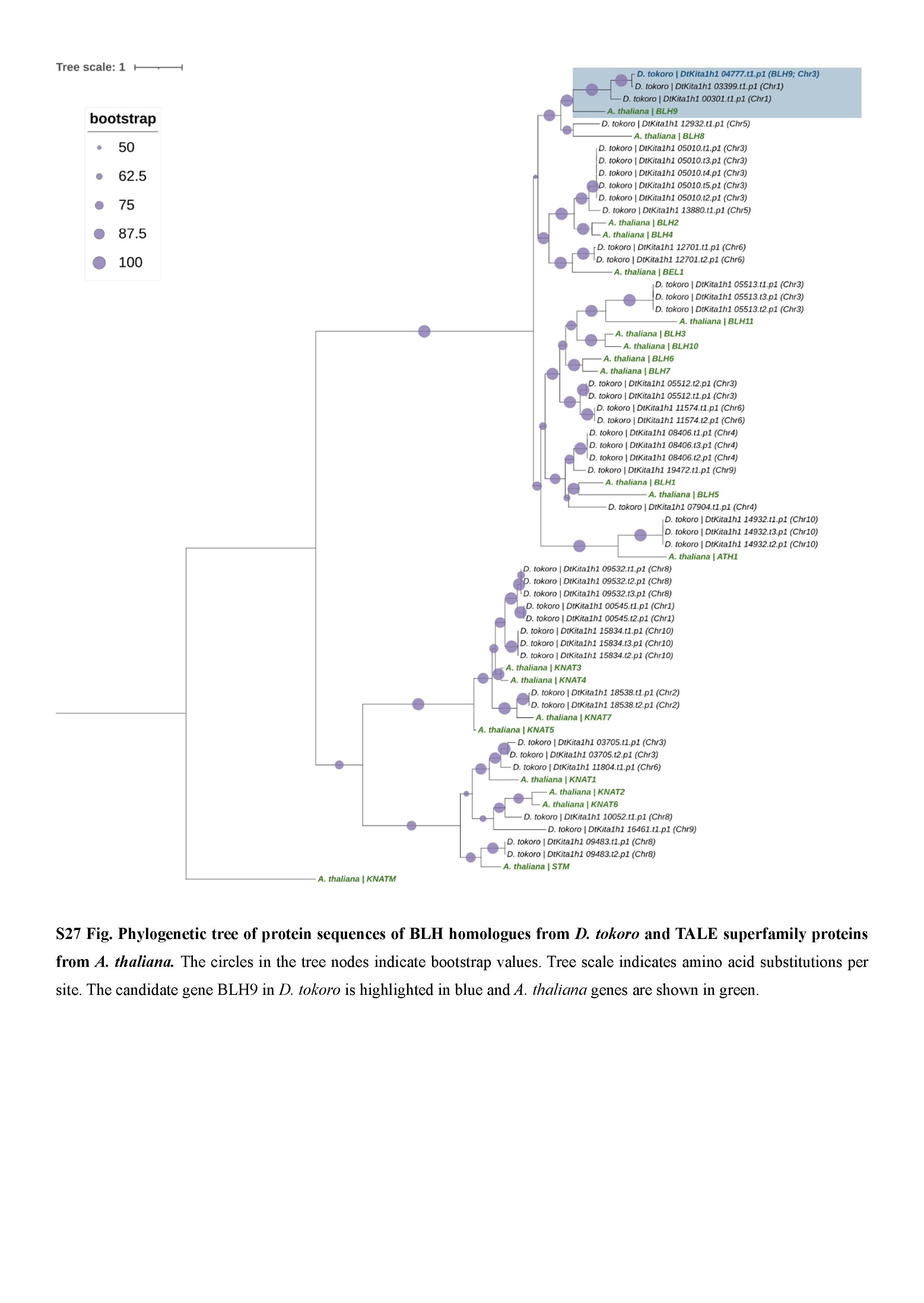

Supplement: S27 Fig — (TIF) [file pgen.1012123.s028.tif]

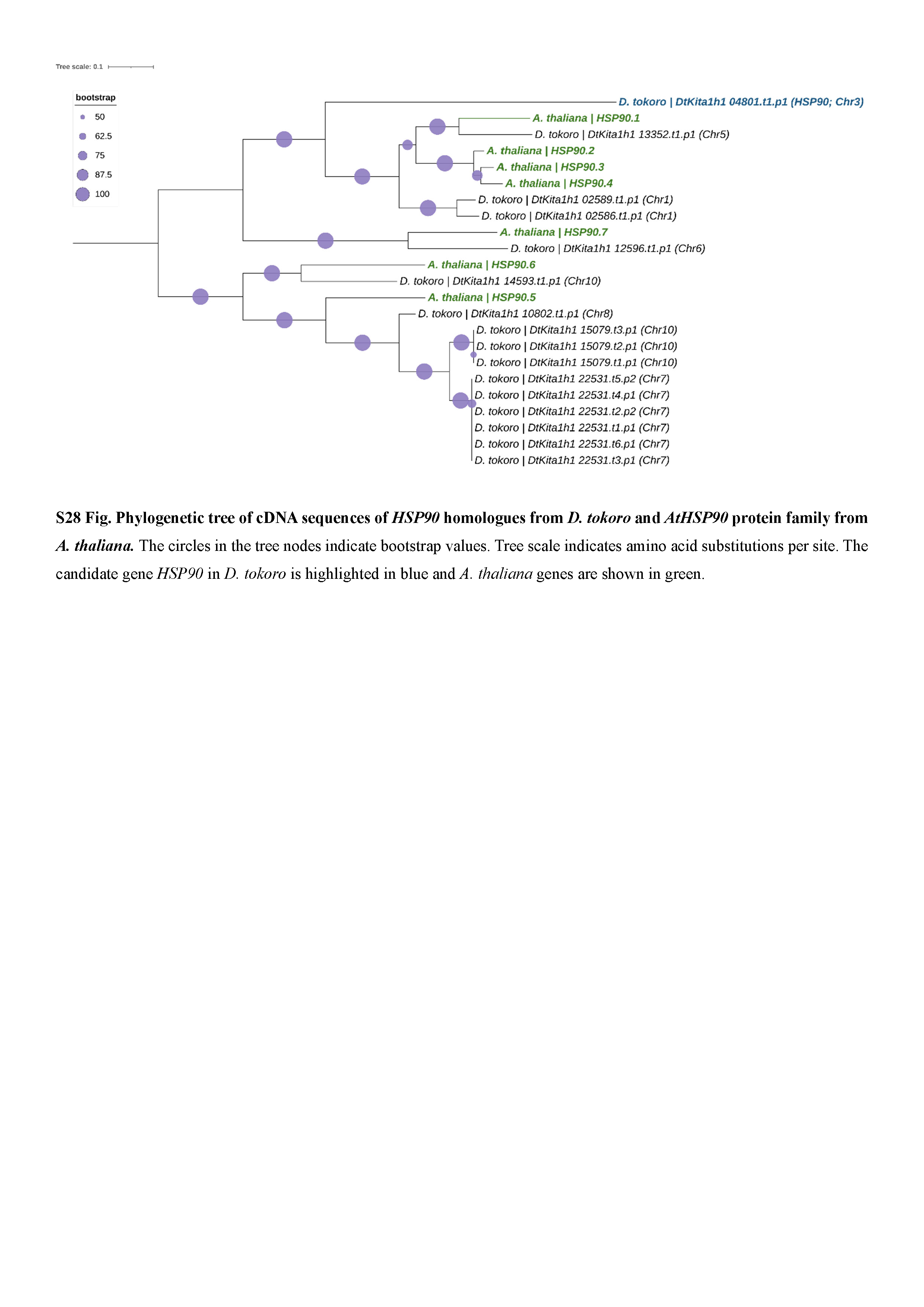

Supplement: S28 Fig — (TIF) [file pgen.1012123.s029.tif]

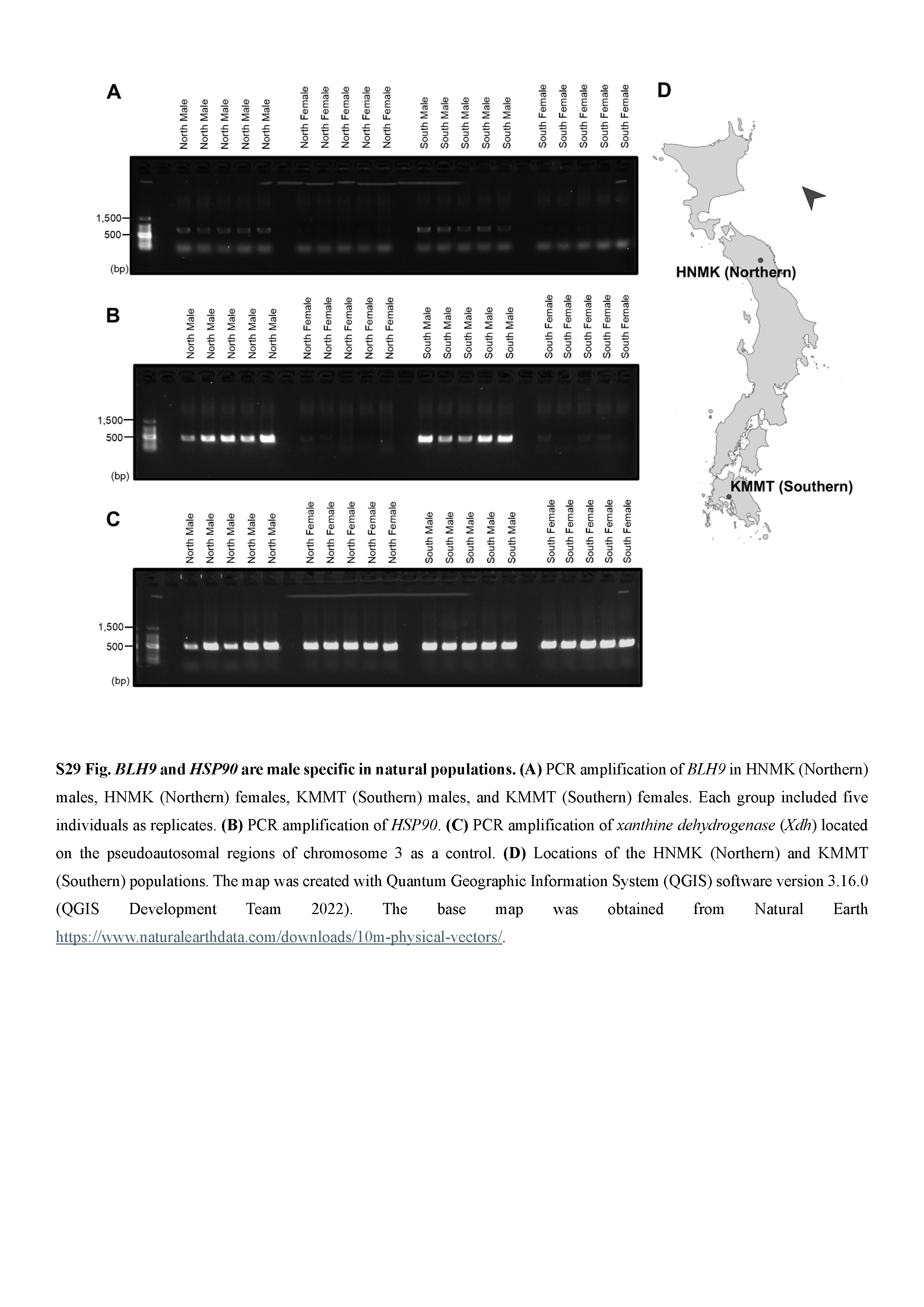

Supplement: S29 Fig — (TIF) [file pgen.1012123.s030.tif]

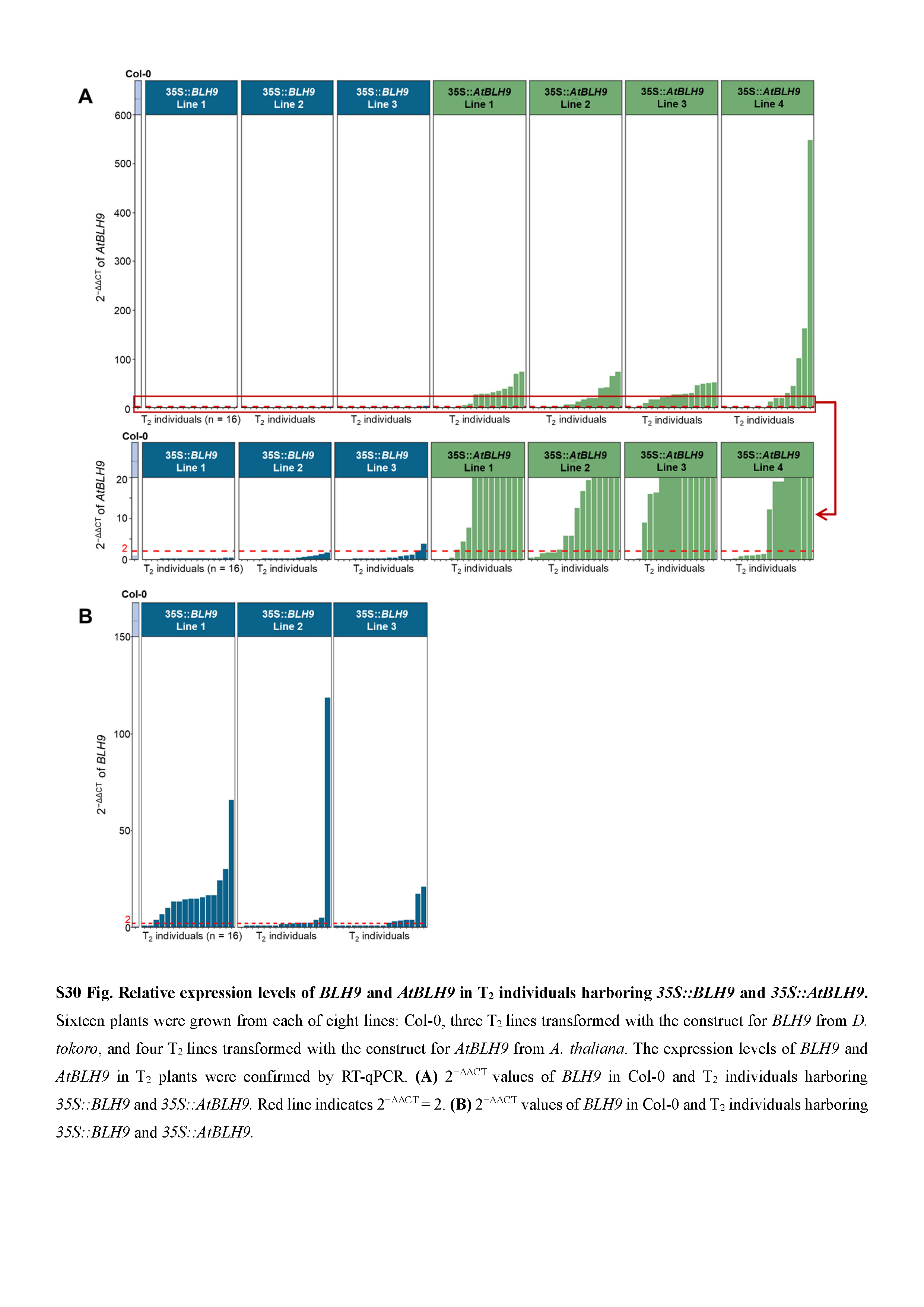

Supplement: S30 Fig — (TIF) [file pgen.1012123.s031.tif]
