# Supplementary material for: Whole-genome sequencing reveals a possible molecular basis of sex determination in the dioecious wild yam Dioscorea tokoro
Source: PLoS Genet. 2026 Apr 20;22(4):e1012123. doi: 10.1371/journal.pgen.1012123 (PMC13128126; doi:10.1371/journal.pgen.1012123)
Supplement: Attachment — Submitted filename: Response to Reviewers.docx [file pgen.1012123.s043.docx]

**Response to reviewers**

Dear Editor,

Thank you very much for taking care of our manuscript PGENETICS-D-25-00572. We are pleased to submit the fully revised version taking the valuable comments of you and the three reviewers.

In the following replies to the reviewers, we re-numbered the comments. Original comments are in black, and our replies are in blue letters. The line numbers in our replies correspond to those in the current (revised) version of the manuscript.

**Reply to the Editor:**

**Additional Editor Comments:**

1. We have now received three referee reports for your manuscript on the elucidation of the genetics of sex determinism in the wild yam Dioscorea tokoro. All referees found merits in the manuscript; however, several serious concerns were raised, in particular about the assembly quality, the lack of clarity about SNP analyses, non-optimal mapping for RNAseq investigation, the lack of dS analyses, the lack of support for hemizygosity and allelic relationships, as well as the lack of discussion of the apparent gain-of-function mutations, of the relevant literature and of technical limits.

I also found that the study is very interesting, with the discovery of the sex chromosomes and of candidate genes for sex determination in a new plant, that are much less studied than animals on these aspects. The study uses both genomics and experimental validation. However, I also have to agree that the manuscript lacks clarity and important analyses, and that some results lack supports due do non-optimal methodological choices. In addition, the beginning of the introduction and the end of the discussion should broaden the context, by replacing the findings in comparison to other plant genera too. The context in the introduction and the discussion is sometimes awkwardly explained (see some examples below). More importantly, the inference of the sex determining region being in the pericentromeric region seems unwarranted: transposable elements also accumulate in non-recombining regions; the presence of many TEs is not sufficient evidence for being a centromeric region. The statement L214 also seems wrong about TEs suppressing recombination, the causality is opposite, with TEs accumulating on non-recombining regions. I also agree with the referees that some classical analyses are missing, such as plotting the synonymous divergence per gene between the X and the Y chromosome along the X chromosome to further support the location of the sex-determining region and to look for stepwise extension of recombination suppression. The results P12 also lack some features of the sex-determining region: size, number of genes, TE enrichment, …

I would encourage resubmission if you are able to revise the manuscript along these lines. The referees also provide a list of excellent additional suggestions and questions, which should also be addressed.

Thank you very much for your positive comments on the manuscript. We have revised the manuscript in accordance with your comments as well as the reviewers' comments.

**More specific comments:**

2. -L45: the male or female sex is heterogametic, not the sex chromosome

Thank you for the comment. We corrected it in L44-45. “The sex phenotype is usually controlled by either male or female sex heterogametic system (XY/XX or ZZ/ZW).”

3. -L47-48: unclear, explain better and cite examples; or delete the sentence, not sure this is really useful for the paper

We removed the sentence.

4. -L48-49: the transition between the genetic system (type of sex chromosomes) and the genes involved is abrupt, as it if the two concepts were confounded, while they are completely different.

Thank you for the comment. We added sentences to connect the two concepts in L46-49.

“Recent advances in genome sequencing, however, made it possible to identify small sex-linked regions, which allowed the discovery of major sex-determining genetic components over the past decade [6].”

5. -L68-69: unclear if different systems have been proposed for the same species or different species.

We rephrased the sentence in L70-71.

“multiple sex-determination systems have been proposed for different species in the genus”

6. -L77-78: awkward wording

We rephrased the sentence in L79-81.

“Different sex-determination systems occur within individual sections (a taxonomic rank below the genus) of the genus.”

7. -L89: a ZZ/ZW system

We corrected it in L92.

8. -L124-125: explain what is Waka1 and Kita1; how do you know this is a XX or XY genotype?

We provided explanations of the samples Waka1 and Kita1 with their presumable XX/XY genotypes based on a previous study in L126-132: “A previous linkage study suggested that this species employs an XY/XX male heterogametic sex-determination system [34]. Therefore, we attempted to generate a consensus assembly from the filtered ONT reads from a female individual (Waka1) collected from Tahara, Wakayama Pref., Japan, presumably with XX genotype. We also obtained separate assemblies using filtered ONT reads for haplotypes 1 and 2 of a male individual (Kita1) collected from Kitakami, Iwate Pref., Japan, presumably with XY genotype.“

9. -L130: contig assemblies

We changed it to “contig assemblies” in L136.

10. -L138: explain what is a pseudo-testcross approach

We added a schematic diagram for the pseudo-testcross method in Fig S3. We also revised the main text in L144-148: “To anchor the assembled sequences to chromosomes by linkage analysis, we used a pseudo-testcross approach [48] (Fig 1F), a method to address the linkage of two DNA markers, both of which are heterozygous in one parent and homozygous in another parent, by testing their co-segregation or independent segregation in the F_1_ progeny population (Fig S3).”

11. -L173: a test or tests

We changed it to “tests” in L183.

12. -L180 a component

We corrected it to “components” in L192.

13. -L184: what are PA markers? avoid abbreviations as much as possible

PA markers indicate presence/absence polymorphism markers. We explained it in the main text in L195-198: “In the genomic region associated with sex phenotype, we detected a higher concentration of presence/absence polymorphism (PA) markers (188 markers in the 10-Mbp region around the marker showing the peak association value) than SNP markers (20 markers in the 10-Mbp region)”

14. -L233/ clarify what is the linkage distance exactly and how it has been measured.

We added an explanation of the linkage distance in L254-L256.

“Linkage distance, recombination frequency between a pair of DNA markers on a chromosome, was obtained based on the segregation of SNP markers among the F_1_ progeny.”

15. -L235: the pattern

We corrected it in L257.

16. -L237: I do not understand, there seems to be several inversions

Thank you for your comment. We tried to compare linkage distance (recombination frequency among F_1_) and physical distance of two genomic positions. If the former is significantly lower than the latter, it suggests recombination suppression in the genomic region. To clearly explain this, we changed the sentence in L254-262 as follows: “Linkage distance, recombination frequency between a pair of DNA markers on a chromosome, was obtained based on the segregation of SNP markers among the F_1_ progeny. Although we observed a small reduction in linkage distances as compared physical distances in the center of chromosome 3, the pattern of relationship between linkage and physical distances was not greatly different from those of other chromosomes (Fig S20, S21). This suggests that the NRYs, even if exist, may be restricted to the small X- and Y-specific regions near the centromere with suppressed recombination, while most part of chromosome 3 constitutes pseudoautosomal regions (PARs) undergoing recombination.”

17. -L262-268: this should be listed in a Table rather than in the text

We removed the list of Y-linked genes from the text and provided them in Table 1.

18. -LL342: this should be stated in the result section, with the number of genes

We moved the sentence to the result section in L224-226: “These chromosomes are differentiated into X- and Y-specific regions spanning ~3.4 Mb with 158 genes (Table S3) and ~2.5 Mb with 34 genes (Table 1), respectively.”

19. -L346, 355 and elsewhere: no abbreviation at the beginning of a sentence

We corrected them in L392, 425, 615.

20. -Do not cite Figures in the discussion, they should have been explained well enough in the result section.

Thank you for the comment. We removed the citation of Figures in the discussion.

21. -L366-367: unclear if you mean the genus or the species; the genus is cited but the introduction gives a lot of results on this across the genus?

We revised as “Despite the previous studies of sex-determination systems in the genus *Dioscorea*, the molecular basis for its sex determination has not been reported.” in L455-456.

22. -L393-L394: not sure this inference is warranted, it is not because you have two candidate genes that the cited hypothesis is supported: only one could actually act, or one could have been recruited later depending on the exact function.

Thank you for your comment. We toned down the inference and revised the sentence in L481-L482.

23. -The discussion recapitulates too much the results without broadening the context.

We revised the discussion to place the results in a broader context, especially in L413-L453 and L481-L506.

24. -All abbreviations should be defined in all legends.

We defined all abbreviations in the legends.

25. -Gypsy elements have been renamed into Ty3 elements

Thank you for your suggestion. We replaced “Gypsy” by “Ty3”.

26. -The legend in Figure 3 includes unwarranted inference about pericentromeric regions (see above)

We revised the legend in Figure 3.

Dear Editor, Again, thank you very much for your valuable comments to improve the manuscript. And thank you for your precious time and effort to help us.

**Reply to the REVIEWER #1:**

27. Kudoh et al. present in this manuscript a high-quality genome assembly of Dioscorea tokoro. Through a combination of comparative analyses between male and female populations, mapping of random sequencing reads, and direct comparison of the X and Y chromosomes, they identify the sex-determining gene as residing in the pericentromeric region of the Y chromosome. They further narrow down the candidate genes using detailed expression data and conduct functional analyses for some of the candidates.

First, the genome assembly, based on current standards including ONT data and extensive use of genetic anchoring, results in a highly refined genome. From this assembly, the authors successfully identified X- or Y-specific regions, while recombination inactivation would not substantially evolve surrounding sex-determining regions in this species. This suggests that the sex chromosomes are either evolutionarily recent or have remained homomorphic over time.

They performed transformation experiments on a BLH-like sex-determining candidate gene using Arabidopsis, a model plant, which is a sound decision. This is because, from a theoretical standpoint regarding the evolution of dioecy, whether under a one-factor or two-factor model, at least one of the sex-determining factors is likely to act as a dominant negative. In the evolutionary context, such a factor would function as a gain-of-function suppressor of feminization. From this perspective, selecting a model plant for functional validation is highly appropriate, and given prior studies of sex-determining genes, it is reasonable to expect that transformation in a model species will produce informative phenotypes, an expectation that has been met in several past cases, like in Diospyros or Actinidia.

The identification of candidate sex-determining factors in a plant species like Dioscorea, which has undergone multiple transitions in its sex determination system, is an outstanding work. The system identified in this study could serve as a starting point for gaining various new insights in sex chromosome/determinant turnovers.

Overall, I nicely enjoyed reading this manuscript. I highly rate the authors’ detailed analyses throughout the study. I would like to raise three major (but simple) points of concern here. While it may not be absolutely necessary to address all of them, I am confident that these comments would help improve the quality of this manuscript.

Thank you very much for your positive evaluation of the manuscript. We incorporated your suggestions to improve it as you see below.

28. It would further improve the manuscript if some discussions are added regarding the evolutionary context and estimated age of the sex-determining region. This is particularly relevant given that Dioscorea species are known to exhibit frequent turnovers of the sex-determining locus (or potentially genes). How ancient this sex-determining factor (in D. tokoro) is, and over what evolutionary timescale it has persisted, even in the absence of a fully developed recombination-suppressed region, is a key question. To address this, the detection of synonymous substitution rates (dS) between X and Y alleles (or dSxy) would be informative. In the MSY (male-specific region of the Y chromosome), even if synteny or sequence conservation is no longer detectable, X–Y alleles often remain scattered throughout the region (please see an example in Silene latifolia, Moraga et al. 2025; Akagi et al. 2025). By analyzing the distribution of dS values among these alleles, one can infer the age and evolutionary trajectory of the sex-determining region. An important question is whether this region is truly male-hemizygous. As will be mentioned later, it would be helpful to clarify whether the term “Y-specific” refers to a region that is strictly hemizygous (present only on the Y) or if X–Y allelic pairs still exist. If such gametologs are present, their dS values might provide insights into the approximate age of the establishment sex-determining system.

Thank you for the valuable comment. We compared DNA sequence divergence of the genes on the Y- and X- specific regions following the methods of Akagi et al. 2025 and Horiuchi et al. 2023 (Fig. S22). Accordingly, we found a total of 13 genes on Y regions that have corresponding gametologs on X-specific regions. The average dS value of the X-Y gametologs was 0.032. As compared to the average divergence of genes between *D. tokoro* and *D. rotundata* (peak of dS value: 0.37) or *D. tokoro* and *D. alata* (peak of dS value: 0.39), dSxy is ~10%, indicating that the divergence time between Y- and X- located genes are not as old as the divergence between the two species. This result was added in L266-L283. The methods for calculation of dS were added in L750-759.

29. Regarding the phenotypic evaluation of the candidate gene, the result showing a “fruitless” phenotype would suggest the possibility that it functions as a SuF (suppressor of feminization) (by the way, more detailed phenotyping of the transformants, especially in flower organs, may be resulted in better conclusions, I guess). If so, it is likely a gain-of-function of a dominant-negative function. This raises the question of how such a gain-of-function might have occurred within the monophyletic clade given in Figure 5A. Typically, gain-of-function mutations arise through neofunctionalization of duplicated genes or changes in expression patterns (like Y-CLV3 in S. latifolia, SOFF for Asparagus, Shy Girl for Actinidia, all SuF), albeit with a few exceptions. Is there any indication of such a scenario in this case? In other words, does the branch leading to Dioscorea BLH1 in Fig. 5A show any unique features that might point to such a change? In the phylogeny, the use of Arabidopsis thaliana alone as a comparative reference may limit the resolution. A more focused analysis restricted to narrow BLH-like genes, including those from other species (especially monocots), could provide a clearer picture of the gene's evolutionary history and possible functional divergence.

Thank you for your comment. We revised the phylogenetic tree of TALE superfamily by extending the entries of the clade containing *D. tokoro* BLH9 (Fig. 5A). Specifically, we included genes from monocotyledonous genera: *Asparagus*, *Dioscorea*, *Oryza* and *Phoenix*. This tree shows that *D. tokoro* Y-specific gene BLH9 is closely related to a *D. tokoro* gene DtKita1h1 03999.t1.p1 located on the chromosome 1. This gene may be the ancestor of Y-specific BLH9 gene. This result was also supported by the phylogenetic tree including all putative TALE superfamily genes of *D. tokoro* (Fig. S23). This result was added in L330-332, L356-L360 and discussed in L498-506.

30. The allelic status (or X–Y gametolog relationship) should be clearly described, especially for the candidate genes (e.g., those in Table 3). The authors suggest that these genes are expressed at higher levels in males than in females. If that is the case, do they have corresponding X alleles (or X gametologs)? Alternatively, are they Y-specific at the genomic level, lacking X allele (I don’t mean the X-Y synteny)? This point also relates to the described query regarding the detection of dS between X and Y alleles (or dSxy). The term “Y-specific” may be better to more finely defined (depending on the situation), in terms of “synteny level”, “genomic level”, “gene level (I mean no existence of the X-allele, even in MSY)” or “expression level”.

We revised Table1 and added the information about the allelic status, Y-specific or X-Y gametologs. We also added a list of 13 possible X-Y gametologs in Fig. S22B and their genomic localization in Fig. S22C.

**Minor points**

31. L. 49 Diospyros lotus is generally called Caucasian persimmon.

Thank you for the comment. We corrected it in L51.

32. L. 58 There has been about 10 “suggested” sex determinant candidates but most of them have not been experimentally validated. Also from this viewpoint, physiological characterization of the candidate gene (in this study) is worth to publishing.

Thank you very much for your positive comments.

33. L. 77-78 A representative genus including both XY and ZW systems would be Populus or Salix, of which molecular mechanisms have been (at least partially) uncovered (although I will leave it up to the authors to decide whether to include that information or not.)

We added the previous reports of *Populus* and *Salix* in the discussion in L443-446.

34. Tables 1 & 2 may be better suited as supplementary data, considering the recent standards in genome biology for plants.

Thank you for the comment. We moved Tables 1 & 2 to supplementary data, Tables S1 & S2.

35. Similarly, I would place Tables 4 and 5 in the supplementary materials. Contents of Table 4 are barely mentioned in the main text. Table 5 presents BLAST results for the candidate genes. If anything is essential in the main text, it would be a figure showing their phylogenetic relationships, that is, a phylogenetic tree (like given in Fig. 5A).

We moved Tables 4 & 5 to supplementary data, Tables S4 & S5. Again, thank you very much for your comments to improve the manuscript.

**Reply to the REVIEWER #2:**

36. Kudoh et al. report the identification of sex chromosomes in the dioecious yam Dioscorea tokoro, an endemic species. Using Oxford Nanopore (ONT)-based genome assemblies of both male and female individuals, they identify two candidate sex-determining genes through transcriptomic analysis. Notably, this is the first study to investigate sex determination in D. tokoro specifically, although sex determination has been previously studied in other species of the Dioscorea genus, including some recent genomic studies. Given the variability of sex determination mechanisms within Dioscorea, this study provides a valuable contribution.

Among the strengths of the work, one of the two male haplotypes appears to be of good quality based on standard assembly metrics (Table 1). The identification of sex-determining gene candidates, as presented in Figures 4 and 5, looks solid and convincing. The authors use a comprehensive RNA-seq dataset, including both reproductive and vegetative tissues, multiple developmental stages, and both poly(A) and small RNA libraries. Additionally, one of the candidate genes was experimentally tested in Arabidopsis, which strengthens this part of their work.

On the downside, the female genome assembly is of relatively poor quality, with a high number of contigs and a low N50. The second male haplotype is also relatively short. These limitations are reflected in discrepancies between the genetic and physical maps shown in Figures S7–S9. The absence of Hi-C data, particularly for the female assembly, likely contributes to these inconsistencies.

Thank you very much for your overall positive comment on the manuscript and the suggestions to improve it. We thought about trying Hi-C, but due to time and resource limitation, we could not perform it. Instead, we tried confirmation of the assembly continuity around the X- and Y-specific regions with additional analysis of the genome assemblies. The corresponding methods and results were added in Section 4.3, *Confirmation of the assembly continuity around the X- and Y-specific regions*, in the Supplementary Materials and Methods. First, we performed the assembly by two methods, PECAT and Hifiasm. The results of dot-plot analysis are shown in Fig. SM3, which indicate that independent assembly methods give consistent results. Also, following the suggestion by Reviewer 3, we tested the continuity of these regions by aligning ONT long reads to the assembled contigs (Figs. SM4, SM5), which suggests no obvious switch of assembly between X- and Y- regions.

37. A key issue is that the quality of the genome assemblies is not adequately discussed in the manuscript. While Table 1 contains important assembly statistics, these are not discussed. Similarly, the mismatches between the genetic and physical maps in Figures S7–S9 are not addressed. Moreover, the fact that different assembly pipelines were used for the male and female genomes is neither discussed nor justified. These points should be clearly addressed in the manuscript.

Thank you for the comments. We discussed the differences in the quality of genome assemblies of female (Waka1) and haplotype 1 and haplotype 2 of male (Kita1) in detail in the discussion (L392-411). We explained the mismatches between genetic and physical maps in L405-407: “In the anchoring step to construct chromosomal-level reference genomes, mismatches between the genetic and physical maps were observed. This may be caused by the inaccuracy of linkage maps due to limited number of F_1_ progeny used for mapping."

We also added the reason for the use of different assembly pipelines in L632-635.

38. An important analysis is the coverage-based detection of sex-linked regions presented in Figure 2. From the methods, it appears that unmasked genomes were used for this analysis, and the authors acknowledge that repetitive elements may introduce noise. A straightforward solution, widely adopted in sex chromosome studies, is to perform male/female coverage comparisons using repeat-masked genomes. This would reduce background noise and improve signal specificity.

Following your suggestion, we tried male/female coverage comparisons using the repeat-masked genomes. However, 80–90% of the sequence was masked in the centromeric region containing the sex-determining locus, and as a consequence, this did not improve signal specificity. The following figures show the male/female short reads depth comparisons (A, C) and N content (B, D) in the chromosome 3 based on repeat-masked genome sequences.

39. The section on recombination on the sex chromosomes (p. 12–13, lines 231–240) is somewhat unclear. The authors seem to be identifying the non-recombining region (NRY) and pseudoautosomal regions (PARs), which are standard concepts in sex chromosome studies. Explicitly using this terminology would improve clarity. It is also unclear how Figures S14 and S15 relate to the recombination data presented in Figures S7–S9; this relationship should be clarified.

Thank you for your comment. We used the terms NRY and PAR in the text to clarify the meaning (L259-L262). We also added Fig S5 to explain the difference between Fig S9–S11 (previous Fig. S7-9) and Fig S20–S21 (previous Fig. S14-15). Fig. S9-S11 are based on all DNA markers including SNPs and PA markers, whereas Fig S20-S21 are based only on SNP markers. The latter analysis was performed since SDR shows X- and Y- specific regions and we judged that the use of PA marker in such regions may not provide correct information for linkage analysis. We explained it in L740-742.

**Other comments:**

40. p. 4, line 38: The claim that “Approximately 30,000 dioecious plant species have been identified, representing 5–10% of angiosperm species” appears to be incorrect. According to Renner (2014), already cited in the manuscript, the estimated number is closer to 15,000 species. Please revise accordingly.

Thank you for the comment. We revised the estimated number to 15,600 in L38.

41. Methods & Supplementary Methods: Please indicate the ONT flow cell chemistry used (e.g., R9.4.1, R10.4.1, etc.). The sequencing error profile varies significantly between chemistries, and this information is important for interpreting the assembly quality. A brief discussion of the limitations of ONT, particularly for older chemistries, would also be valuable. The quality score of the consensus sequence is not mentionned.

We added the ONT flow cell chemistry in L564-567 of the materials and methods. The Waka1 (female) libraries were sequenced on R9.4.1 flow cells (FLO-MIN106) using a MinION Mk1C device, and the Kita1 (male) libraries were sequenced on R10.4.1 flow cells (FLO-PRO114M) using a PromethION 2 Solo device. In Discussion, we addressed the difference of assembly quality based on the ONT versions in L401-404. The quality scores of the consensus sequences were mentioned in Table SM5 and SM6 of the Supplementary materials and methods.

42. p. 12, lines 215–217: The statement that “X- and Y-specific regions were located in a genomic region with lower gene density and higher density of repetitive DNA sequences, represented by gypsy long terminal repeats (LTRs)” (Fig. 3A, B) should be revised. The gene/repeat density does not directly indicate recombination suppression, although it may correlate. The primary evidence for suppressed recombination is the recombination map (Figures S7–S9). Please rephrase accordingly here and in the Discussion (pp. 17–18, lines 349–351).

Thank you for your comment. We revised the section *X- and Y-specific regions are likely located in the pericentromeric regions* (L228-L283). We revised the statement as: “The X- and Y-specific regions revealed to be located in a genomic region with lower gene density and higher density of repetitive DNA sequences, represented by Ty3 long terminal repeats (LTRs) (Fig 3B, C). Centromeres and pericentromeric (near the centromere) regions are known to have suppressed recombination [58] and contain a higher density of retrotransposons and transposons [59,60]; particularly high levels of Ty3 LTRs are reported in multiple monocot species [61–63]. Co-localization of X- and Y-specific regions to the gene-paucity and repetitive DNA-rich regions suggests that *D. tokoro* X- and Y-specific regions likely correspond to the pericentromeric regions (near the centromere) of chromosome 3.”

We also revised the Discussion in L419-422: “Moreover, recent studies suggested that sex chromosome formation may occur in regions with pre-existing recombination suppression [78], raising the possibility that the establishment of the SDR in *D. tokoro* was also influenced by such pre-existing recombination suppression.”

43. p. 17, lines 341–342: “This chromosome is differentiated into X- and Y-specific regions spanning ~3.4 and ~2.5 kb, respectively.” This should almost certainly be Mb, not kb.

Thank you for the comment. We revised to Mb and moved the sentence to L225.

44. pp. 19–20, lines 393–405: The discussion on the two-gene model of sex determination and gene number comparisons with other systems is currently superficial. A more nuanced discussion, grounded in recent reviews and studies across plants, would enhance the manuscript. Please ensure all relevant literature is cited.

Thank you for the comment. We added the relevant references and discussion in L481-L506.

**Reply to the REVIEWER #3:**

45. In this manuscript, Kudoh et al assembled the genome of one male and one female of the dioecious plant Dioscorea tokoro, using Oxford Nanopore Technology (ONT) long read sequencing. The ONT contigs were scaffolded using a genetic map based on RAD-seq of a family. The authors use a SNP analysis to determine the species carries an XY system. Then they use a coverage analysis to locate the sex-linked regions. The male-specific region is located in chromosome 3 pericentromeric region. Both the X and the Y haplotypes are lowly recombining in males and females. Using RNA-seq data from various tissues and developmental stages, the authors propose two sex-determining genes (male-biased in their expression) that could be responsible for sex determination. I found the manuscript interesting and it provides a lot of new resources on this species. However, some control analyses are required to ensure the proper assembly of the X and Y specific regions. I also believe there is a problem with the reference used in the RNA-seq analysis, potentially biasing the identification of sex-specific expression. Also, traditional analyses of sex chromosomes are lacking that I would like to suggest the authors include in a revised version of their manuscript. I also would like to suggest that the authors tone down their affirmations in some places, as the sex-determining genes have not been validated yet.

Thank you very much for your valuable comments. We did our best to improve the manuscript by incorporating your kind suggestions.

46. Assemblies:

It is not clear to me whether the X and Y haplotypes were assembled properly. It is particularly difficult to assemble them due to high repeat content. What is more, both haplotypes don’t recombine, making the genetic map useless to scaffold the X and Y-specific region. The authors could rely on other technology such as BioNano or Hi-C to check the quality of their assembly. If funding is an issue, I would recommend at the very least to map the ONT reads onto the assemblies and check if the X and Y-specific regions are contiguous. In the case of the male genome assembly, mapping of ONT reads onto haplotype 1 and 2 should not lead to heterozygosity. If heterozygosity is detected, the Y haplotype might in fact be a mosaic between the X and the Y. Haplotype 2 of the male genome seems problematic because it has fewer genes and it is shorter. If haplotype 2 really is the X haplotype, it should resemble the female genome assembly, but this is not the case, suggesting there are issues with haplotype 2 assemblies. Lines 162-164 of the main text should be toned down in agreement with these limitations.

Thank you for your comment. We aligned female and male ONT reads to the female and male reference genomes including X- and Y- specific region. It shows the X- and Y- regions are contiguously covered by long ONT reads (please see Fig. SM4, SM5 in Supplementary Materials and Methods). Also, we reconducted *De novo* assembly using other software (PECAT and Hifiasm). The new assemblies by Hifiasm included telomere-to-telomere contigs, and the comparison with the constructed assemblies of Kita1 (male) suggested the constructed assemblies is not software-dependent and seems to be reliable (see Fig. SM3).

47. SNP analysis:

It took me a lot of time to figure out what PA markers were, how they differed from other markers and how the SNP analysis had been made. In the end I had to guess. A figure illustrating the SNPs and their segregation would greatly help the understanding of the method (it could be supplementary).

Thank you for your comment. We added a Supplementary figure to illustrate the SNP and PA markers to be used for mapping (Fig. S5) and cited it in L152.

48. The authors should explain better in the text how they are able to exclude a ZW system using their SNP analysis. Importantly, excluding a W chromosome cannot be done when mapping is made on male haplotype 1, therefore the female reference is also very important and should be stressed (it is currently in the Supp data).

Thank you for your comment. We added Fig. S12 to illustrate how we judged that *D. tokoro* has XY male heterogametic system and cited it in L183. If *D. tokoro* has ZW female heterogametic system, female-parent-heterozygous markers should show association with sex phenotypes around the sex-determination regions, but such association was not observed.

49. The association analysis between SNPs and sex provides results that are not precise (the entire chromosome 3 is sex-linked), suggesting a lack of statistical precision. I would suggest repeating the analysis with equal numbers of males and females, rather than unbalanced numbers which increase variance and can inflate p-values.

Thank you for your comment. We tried the association analysis and linkage analysis with equal number of male and female F_1_ (Fig. S14). The result was identical to that shown in Fig. S13.

50. In Figure S10B it is confusing to see that male alleles are detected with the female reference. I suspect this is due to the presence of X-hemizygous SNPs? I suggest the authors separate in their analyses the different SNP and PA marker types: X-linked, Y-linked and show them in different Figures. This would help to identify Y-specific regions and X-hemizygous regions. Alternatively, the authors could use programs developed with this aim such as SEX-DETector or SDpop.

Thank you for the comments. In Fig. S13B (Fig. S10B in the previous version), the blue plots indicate male-parent-heterozygous markers based on the presence/absence polymorphism information in the parents (Fig. S5). The male- and female-parent-heterozygous markers are located on all chromosomes (including pseudo-autosomal regions) and does not mean that these are male- or female-specific alleles. After obtaining the male- and female-parent-heterozygous markers, we conducted the association analysis (Fig. S12). In the case of an XY sex-determination system, the association between male-parent-heterozygous markers and sex phenotypes is predicted to be strongest within the sex-determining region and to gradually weaken with increasing distance from it. Because of the linkage, we observed the association peak with male-parent-heterozygous markers in Fig. S13B.

We tried the association analysis using 4 types of markers: (A) X-linked (aligned on X chromosome) SNP markers, (B) X-linked PA marker, (C) Y-linked (aligned on Y chromosome) SNP markers, (D) Y-linked PA markers. The results were identical to that of Fig. S13.

We also tried SDpop using short reads of 5 females and 5 males newly obtained from the northern population of Japan. We aligned the short reads to the female reference genome to obtain the X-hemizygous sites. However, in the XY-type sex chromosome model of SDpop, the proportions of sites of X-hemizygous were low: autosomal, 0.99; X-hemizygous, 0.0046; XY-gametologous, 0.0021. This may be because the Y chromosome and the X chromosome share most of their regions as autosomal regions (pseudo-autosome), with differentiation occurring only in a small region possibly near the centromere.

51. Read coverage analysis:

I would recommend re-running the read coverage analysis after excluding the coverage information from repetitive regions. I am currently not convinced that the identified region is indeed sex-linked, because coverage information is not consistent with the expected 1X in males and 2Xs in females.

Thank you for your comment.

The coverage is not consistent with the expected 1X in males and 2Xs in females because repetitive elements likely obscure the coverage estimates, particularly in the possible centromeric region. However, we observe a female/male coverage difference only on chromosome 3, even though all chromosomes contain repetitive elements around their centromeres. We added the read coverage analysis for all chromosomes in Fig. S16 and Fig S17 and explained in L211-215.

Following the comment of Reviewer 2, we tried the read coverage analysis using repeat-masked genomes, but this did not improve signal specificity because the regions around the centromere were mostly masked. Please see the figures for comment 38.

52. RNA-seq analysis and candidate genes:

It is not appropriate to estimate female expression levels from mapping on a Y haplotype. X and Y haplotypes have diverged and prevent mapping of X reads onto the Y reference and vice versa. It is not surprising that the authors find male-biased genes with this method, since female expression is not well measured. I am afraid this bias might have affected the choice of the two candidate genes explored by the authors, which is why I am not convinced about the candidates. It is a shame because a lot of work was done on testing one candidate with over-expression mutants in A. thaliana.

Thank you for the comment. Most Y-specific genes are absent from females, and their expression is undetectable in female samples. For *BLH9* and *HSP90*, we also confirmed their presence in Y-region and absence from X-region by genomic PCR (Fig. S25). We showed the result of mapping of female transcripts to Y-chromosome (Fig. 4E middle) in order to verify that no homologous sequences of Y-specific genes were expressed from X-chromosome or autosomes. To clarify the Y-specificity of genes located in Y-specific regions, we added this information to Table 1.

53. Could the authors specify if stage S0 of developing buds already have organs formed inside? Ideally to study sex-determination the buds should be sampled before organ primordia are formed. It should be noted that female flowers carry aborted stamens, suggesting that sex-determination in females might happen at a later stage. The gene responsible for stamen abortion should therefore be studied at a later stage. Carpel abortion seems very early in males though. This could be discussed in terms of the pathways followed to evolve dioecy in this plant group: monoecy of gynodioecy ? Please see Barrett (2002).

Thank you for the comment. In the target species, *Dioscorea tokoro* which is a climbing plant, the earliest floral organs are formed at the tip of the vine (defined as stage S0; Fig. A). Inside S0, an inflorescence primordium is present, but no distinct pistil or stamen primordia were observed (Fig. B). As the inflorescence develops, buds containing pistil and stamen primordia become visible (Fig. C, D), and develop into mature buds (Fig. E). Therefore, S0 represents the stage before organ primordia are formed, whereas S1 and S2 correspond to stages in which organ primordia are being formed and developed. Figures A–E are shown below.

54. The authors should justify better in the text why they wanted to study sRNAs.

Thank you for the comment. We performed sRNA analysis since there are cases where mi-RNA is involved in the sex determination (Akagi et al., 2014; Müller et al., 2020). We added them in L290-291.

55. A candidate sex-determining gene does not have to be Y-specific, it can be an X/Y genes with a dosage difference between males and females. It should be clarified if the candidate genes have no X copy, if so, where did they come from? Were they translocated from an autosome onto the Y? Do they have autosomal homologs?

Thank you for your valuable comment. Out of the 34 genes in Y-specific region, 13 genes have corresponding related genes (gametologs) in X-chromosome, so we added this information in Table 1 and Fig. S22BC. The two candidate genes, *BLH9* and *HSP90*, are Y-specific and have no gametologs in X-chromosome. To infer the origin of *BLH9* and *HSP90* on the Y-region, we included phylogenetic trees of *BLH9* and *HSP90* genes and their putative homologues (Fig. S23, S24), as well as a discussion on their origins (L498–L503).

56. I am not convinced that BLH9 prevents fruit formation, its phenotype in A. thaliana rather suggests it may lead to sexual dimorphism (differences in plant heights for example), but the plants still have fruits although smaller.

Thank you for your comment. The limitation arises from functional analyses using distantly related model plants, as a genetic transformation system has not yet been established for *D. tokoro*. In such cases, the target traits may not be fully recapitulated. For example, in the case of *SyGI* in kiwifruit, *N. tabacum* transgenic lines expressing *SyGI* under the control of the CaMV35S promoter occasionally exhibited inhibited carpel growth, with 3 of 10 lines showing irregular carpel development (Akagi et al., 2019). These incomplete functional outcomes may result from differences in the gene expression patterns across organs and/or in the downstream regulatory systems between the target dioecious species and the model plants. We added a sentence in discussion in L468-L470.

57. The gene candidates should be discussed in light of their dominance level. The Charlesworths “2 genes model” makes very precise predictions about the recessivity and dominance of mutations that can lead to the formation of sex chromosomes. For X/Y sex chromosomes, the female sterility factor is expected to be dominant and Y linked, while male sterility should be recessive and X linked. Are the results in agreement with this model? Other alternative models involve just one gene, such as in Salicaceae.

For the DESeq2 analysis, were genes filtered based on their fold-change? Were lowly expressed genes removed?

Thank you for your comment. In Discussion (L481-506), we discussed the possible function of BLH9 as the suppressor of femaleness (SuF) and HSP90 as the male-promoting sex determinant (M factor) in: “The two primary candidate genes for sex determination in *D. tokoro*, described above, may be consistent with a major hypothesis for the evolution of dioecy: two sequential mutations affecting male and female fertility are supposed to have led to the evolution of separate sexes controlled by the male-promoting sex determinant (M factor) and the suppressor of female development (SuF) [91–93]”. We think the presence of dominant *BLH9* in the Y-region conforms to the proposed model and described it in L501-503 “These results suggest that the putative dominant SuF gene, *BLH9* in *D. tokoro*, may have been established by gene duplication, whereas the putative M gene, *HSP90* in *D. tokoro*, may have been lost from the X chromosome.” For the DESeq2 analysis, we compared early developmental stages of female and male flowers and selected genes with an adjusted p-value < 0.05 and log₂(Male/Female) > 2. Lowly expressed genes were not removed in the analysis.

58. Other suggested analysis:

It is important to annotate properly X-Y homologs (with permissive blasts to accommodate for X-Y divergence) and identify Y-specific genes and X-hemizygous genes. These genes should be represented in a Figure comparing the X and Y haplotypes with links between X/Y pairs. Their numbers is informative for sex chromosome degeneration level (in particular the proportion of X-hemizygous genes). The age of the sex chromosomes should be estimated with dS on X-Y gene pairs. Potential strata on the X should be annotated also with dS. These results should be confronted with the other species of the clade. I suggest the authors inspire themselves from recent papers in the field such as She et al. (2025). It would help to present a Figure of a phylogeny of other species where the sex chromosomes have been characterized. It is not sufficient to say that the non-recombining sex-specific region is on the same chromosome to say that the system is ancestral, gene content should be compared as well as X-Y dS values. It is possible than the same chromosome became the sex chromosome independently twice.

We performed dS analysis as suggested by Reviewer 1 and provided the data on Fig. S22 and Table 1.

59. Other suggestions:

The title at first read suggests that the authors have identified the sex-determining genes of D. tokoro, which we discover is not the case when reading the manuscript. Please tone done the conclusion of your title. In the abstract “BLH9 is thought to suppress female organ development” is too strong a statement. The gene is male-specific in D. tokoro and does not make A. thaliana female sterile, so there is no base yet to think BLH9 suppresses female organ development, for now it is just a candidate. Again in the abstract “we describe the molecular basis of sex determination in the monocot Dioscorea tokoro”, “describe” is too strong, please rather use explore. In the abstract “Within the Y-specific region, we identified two candidate genes that are likely involved in sex determination” please replace identified with propose. In the abstract “These results shed light on the complex evolution of dioecy in plants” is somewhat vague and in disagreement with the lack of discussion of this topic in the manuscript. I would either suggest the authors remove this sentence from the abstract or indeed discuss this topic in the discussion.

Thank you for your comment. We revised the title and abstract according to your suggestions. We kept the last sentence of the abstract with more discussion in L413-453.

60. Many key references are missing from the sex chromosome literature in this manuscript, I suggest the authors include at the very least the following references: (Charlesworth and Charlesworth 1978; Renner 2014)

More references can be found in this recent review of the literature: (Saunders and Muyle 2024) especially references regarding work by Stephen Wright on lowly recombining regions prior to the formation of sex chromosomes.

Thank you for your comment. We added Charlesworth and Charlesworth 1978; Renner 2014 in L40 and L485. We also added the references and discussion about the recombination suppression and transitions of sex chromosomes in L416-453.

61. In the introduction the authors ask very general questions about sex chromosomes and sex determination but these questions are not really answered in the discussion. The introduction should be re-written to tackle the more specific aims of this study and the clade studied. Very interesting questions can be asked with this Dioscorea model about sex chromosome turnover for example. A better comparison to other Dioscorea species with a Figure showing a phylogeny would be welcome.

Thank you for the comment. We added the discussion in L416-453. The comparison to other *Dioscorea* species was added in Fig S27.

62. Please write sex-determining genes (not determination) throughout the text.

We revised “sex-determination genes” to “sex-determining genes” in the text.

63. The flowcell used for ONT sequencing should be specified, as well as the basecalling model version. For DNA-seq, what was the fragment length, read length and sequencing mode (paired-end?)?

We added the ONT flow cell chemistry in L564-567 and the DNA-seq information in L580-582 of the materials and methods in main text. We also added the information in the Supplementary materials and methods.

64. Line 541 what do you mean by aligned transcriptomes? What version of R/qtl was used (line 600)?

Thank you for the comment. We revised as “To obtain transcriptomes, the filtered RNA-seq reads were aligned to the assembled contigs with HISAT2 v2.2.1 [110]. The transcriptomes were assembled using StringTie v3.0.0 [111], …” in L648-650. We added the version of R/qtl in L687.
