# Supplementary material for: Whole-genome sequencing reveals a possible molecular basis of sex determination in the dioecious wild yam Dioscorea tokoro
Source: PLoS Genet. 2026 Apr 20;22(4):e1012123. doi: 10.1371/journal.pgen.1012123 (PMC13128126; doi:10.1371/journal.pgen.1012123)
Supplement: Attachment — Submitted filename: Response letter_second_revise.PLOSG_v12.0_rt.docx [file pgen.1012123.s044.docx]

**Response to the review**

Dear Editor,

Thank you very much for taking care of our manuscript PGENETICS-D-25-00572. We are pleased to submit the fully revised version taking valuable comments from you and the reviewer #2.

In the following replies to the review, we re-numbered the comments. Original comments are in black, and our replies are in blue letters. The line numbers in our replies correspond to those in the current (revised) version of the manuscript. We do hope this revision is satisfactory for the paper to be accepted by PLoS Genetics.

**Reply to the Editor:**

**Additional Editor Comments:**

1. We have now received two referee reports for your revised manuscript on the molecular basis of sex determination in the dioecious wild yam Dioscorea tokoro. One of the referees still had major concerns, in particular about the lack of delimitation of the sex-determining region and inconsistencies in the GWAS and dS analyses. They recommend additional analyses, notably high-quality recombination maps and plotting the distribution of sex-associated SNPs along the sex chromosome. I concur with these suggestions and add a few further recommendations below. I encourage resubmission if the manuscript is carefully revised along these lines and the other excellent suggestions made by the referee.

Thank you very much for your comments on the manuscript. We added the additional analyses: (A) high-quality recombination maps and (B) plot of distribution of sex-associated SNPs along the sex chromosome. A summary of these results is provided below. We have also revised the manuscript in accordance with your comments as well as the reviewer's comments.

**(A) High-quality recombination maps**

To construct high-quality recombination maps, we incorporated additional RAD-seq data for the parental individuals used for generating the F_1_ population and reconstructed the genetic maps with more stringent thresholds for marker selection. Addition of RAD-seq data increased the total number of available RAD markers. For marker selection, we retained only the alignments with high mapping quality (≥60) and properly paired reads, which made the marker selection more reliable. In addition, we excluded markers affected by segregating distortion in the F_1_ progeny based on a more stringent threshold. In the previous genetic maps, we selected markers following the expected 1:1 segregation by performing a binomial test, assuming that 186 F_1_ individuals segregate equally between the two allelic types. Markers showing segregation ratio with p < 0.001 (corresponding to test statistic Z > 3.29) were regarded as showing segregation distortion. In the new genetic maps, to select the markers more strictly following the expected 1:1 segregation, the markers with p < 0.01 (Z > 2.58) were regarded as having segregation distortion and were discarded. Based on the more reliable markers, we reconstructed the new genetic maps (Fig S9, S10, S11). In the previous genetic maps, several chromosomes showed discrepancies between male- and female-parent heterozygous maps, but in the new genetic maps these inconsistencies were largely resolved. All subsequent analyses were reperformed using the new pseudo-chromosomes based on the high-quality genetic maps. Please refer to our response to comments 12 for the details.

**(B) Plot of distribution of sex-associated SNPs along the sex chromosome**

To plot the distribution of sex-associated SNPs along the sex chromosome, we added two analyses; SDpop (Käfer et al., 2021) and RADSex (Felon et al., 2021) as suggested by the reviewer. SDpop analysis was conducted using Illumina short read sequences of five females and five males collected from natural population. SDpop’s inferences of sex-association clearly indicated the SDR and PAR boundaries in Y chromosome (Fig S20 in the revised version). RADSex analysis was performed using RAD-seq data of 127 flowering F_1_ progeny segregating to male and female, showing the RAD markers associated with sex in the middle of chromosome 3 (Fig S21 in the revised version). Taking these new data into consideration, we redefined the SDRs and performed all subsequent analyses based on these. Please refer to our response to comments 13 for the details.

2. -L15: similar as

We corrected it in L14-15: “BLH9 functions in a similar way as AtBLH9 in *Arabidopsis thaliana*”.

3. -L95, L111, L126 and elsewhere: “employs” sounds too anthropomorphic

Thank you for the comment. We rephrased it in L29, 95, 111, 126, 487 and 538.

4. -L203: coverage instead of dosage? Dosage is usually used for expression and there can be compensation

We corrected it in L202: “Furthermore, the coverage of the male X-specific region is…”.

5. -L230-234: still a bit awkward. Recombination suppression is a key step in sex chromosome evolution. The accumulation of repetitive DNA sequences is a conspicuous feature of non-recombining regions, not only “primitive”, whatever this means. There are several examples in fungi of recombination suppression on mating-type chromosomes without inversions (e.g., <https://academic.oup.com/mbe/article/38/6/2475/6130827>; <https://www.nature.com/articles/s41467-018-04380-9>; <https://pubmed.ncbi.nlm.nih.gov/29074958/>)

Thank you for the comment. We revised the sentences of recombination suppression in L252-259, “Recombination suppression is a key step in SDR evolution. This suppression is caused by various mechanisms, such as chromosome rearrangements including inversions and transpositions [6,54,55]. Recombination suppression has also been reported even in absence of structural rearrangements, accompanied by gene loss and accumulation of repetitive DNA sequences [56,57]. Especially, accumulation of repetitive DNA in the non-recombining regions (NRY) surrounding the SDR are reported as conspicuous feature of plant sex chromosomes and thought to involved in sex chromosome evolution [58–62].”.

6. -L268-283: S is subscript in dS; the idea actually of dS was to plot it along the X chromosome gene order to see if there were evolutionary strata, please add this analysis (e;g., <https://www.science.org/doi/10.1126/science.adj7430>). I don’t find the “Fig S22BC ».

We added the plot of distribution of dS values of all gene pairs across X chromosome (Fig S23). No clear evolutionary strata were detected in the X chromosome of *D. tokoro*. This result suggests that X–Y differentiation in *D. tokoro* may be relatively recent compared with *Silene* and *Humulus* (Akagi et al., 2025). We explained the result in L312-319.

7. -L427, 441, 442, and elsewhere : do not refer to figures in the discussion. There is no figure S27 right ?

Thank you for the comment. We moved the description of figure (Fig S24 in revised version) to Results section (L321-L331).

Again, thank you very much for your time and efforts in handling our manuscript.

**Reply to the REVIEWER #1:**

8. The authors properly revised the manuscript satisfying my requests, and I have no more comments. I am looking forward the publication.

Thank you very much for your previous suggestions and positive comments.

**Reply to the REVIEWER #2:**

9. Despite the additional data and analyses provided in the revised manuscript, I believe that important information is still missing.

Thank you very much for your valuable comments to improve the manuscript. We revised the manuscript by incorporating your kind suggestions.

10. Based on the GWAS, it is clear that chromosome 3 harbors the SDR. However, the precise boundaries of the SDR and the PARs remain unclear. This uncertainty prevents a full and reliable characterization of the SDR. Without a clear definition of these boundaries, it is difficult to robustly assess the gene content of the X and Y haplotypes of the SDR, the extent of X–Y differentiation, or the degree of Y degeneration.

The coverage analysis presented is not entirely convincing and does not allow proper characterization of the X and Y haplotypes within the SDR. The observation that masking the genome does not improve the signal is surprising (figures in the rebuttal), although it may result from low X–Y divergence. In any case, this suggests that coverage analysis is not the most appropriate approach to characterize this SDR, especially since this method was originally designed for highly diverged animal systems.

Thank you for the comment. Following your suggestions, we tried our best to delineate SDR and PAR regions with additional analyses. Please see below for the details of additional results.

11. The dS analysis of X/Y gametologs reveals very low values, comparable to those observed for random orthologs across the genome (new Figure S22). Furthermore, only a few gametolog pairs were identified, and they appear scattered across chromosome 3 — which is unexpected given the low overall X–Y differentiation.

Thank you for your comment. With a careful examination of SDRs in the new pseudo-chromosome 3, we identified 22 X/Y gametologs corresponding to the Y-specific regions (see Fig S22B). Accordingly, the average dS value of X/Y gametologs was 0.23, indicating that the divergence between Y- and X- located genes are not as old as the divergence of the genes of *D. tokoro* from those of *D. rotundata* (dS average = 0.71) and *D. alata* (dS average = 0.70). We also added a figure of distribution of dS values between Waka1 (X) and Kita1-hap 1 (Y) genes across the chromosome 3 (Fig S23). We observed low dS values across the entire chromosome without significant peak in the SDRs, suggesting a low level of X-Y differentiation.

12. The genetic maps are somewhat confusing. Comparing male and female recombination maps is typically informative, as it can reveal the non-recombining Y haplotype and the boundaries of the PARs. At present, several chromosomes show large discrepancies between maps built from male-heterozygous versus female-heterozygous SNPs, and these maps are not consistent between the male and female reference genomes (Figures S20, S21). It is not entirely clear whether male and female recombination data have been directly compared.

Thank you for your comment. To reconstruct high-quality recombination maps, we incorporated additional RAD-seq data for the parental individuals used for generating the F_1_ population and reconstructed the genetic maps with more stringent thresholds for marker selection (see Table A below). Addition of RAD-seq data increased the total number of polymorphic RAD markers between the parents. For the selection of markers for linkage analysis, we retained only the alignments with high mapping quality (≥60) and properly paired reads, which made the marker selection more reliable. In addition, we excluded markers affected by segregating distortion in the F_1_ progeny based on a more stringent threshold. In the previous genetic maps, we selected markers following the expected 1:1 segregation by performing a binomial test, assuming that 186 F_1_ individuals segregate equally between the two allelic types. Markers showing segregation ratio with p < 0.001 (corresponding to test statistic Z > 3.29) were regarded as showing segregation distortion. In the new genetic maps, to select the markers more strictly following the expected 1:1 segregation, the markers with p < 0.01 (Z > 2.58) were regarded as having segregation distortion and were discarded. Based on the more reliable markers, we reconstructed the new genetic maps (Fig S9, S10, S11). In the previous genetic maps, several chromosomes showed discrepancies between male- and female-parent heterozygous maps, but in the new genetic maps these inconsistencies were largely resolved.

| **Table A** | **Previous genetic map** | **Current genetic map** |
| --- | --- | --- |
| **Total number of RAD-seq reads obtained for parental lines** | | |
| Female parent | 2,003,754 | 4,428,694 |
| Male parent | 1,941,994 | 4,109,122 |
|  |  |  |
| **No. of markers before filtering** |  |  |
| *Waka1* |  |  |
| Female-parent-heterozygous SNP & PA | 11,736 | 21,211 |
| Male-parent-heterozygous SNP & PA | 7,870 | 20,378 |
| *Kita1 (hap1)* |  |  |
| Female-parent-heterozygous SNP & PA | 10,365 | 22,515 |
| Male-parent-heterozygous SNP & PA | 9,688 | 20,829 |
| *Kita1 (hap2)* |  |  |
| Female-parent-heterozygous SNP & PA | 7,859 | 16,780 |
| Male-parent-heterozygous SNP & PA | 7,664 | 17,448 |
|  |  |  |
| **Thresholds applied in the selection of markers used for linkage mapping** | | |
| Mapping quality | ≥40 | ≥60 |
| Properly paired read selection | Not applied | Applied |
| Segregation distortion threshold | p < 0.001 (Z > 3.29) | p < 0.01 (Z > 2.58) |
|  |  |  |
| **No. of markers after filtering** |  |  |
| *Waka1* |  |  |
| Female-parent-heterozygous SNP & PA | 4,095 | 1,683 |
| Male-parent-heterozygous SNP & PA | 2,817 | 1,290 |
| *Kita1 (hap1)* |  |  |
| Female-parent-heterozygous SNP & PA | 3,908 | 1,704 |
| Male-parent-heterozygous SNP & PA | 2,711 | 1,224 |
| *Kita1 (hap2)* |  |  |
| Female-parent-heterozygous SNP & PA | 2,867 | 1,216 |
| Male-parent-heterozygous SNP & PA | 2,378 | 1,216 |

In the previous version, we presented two versions of linkage maps; one with SNP and Presence/Absence (PA) markers (previous Fig S9, S10, S11) and the other with SNP markers only (previous Fig S20, S21). To evaluate the relationship between linkage and physical distances, we used the maps based on SNP markers, since the selection of PA markers near SDR was not reliable. In the current improved high-quality maps, marker selection became stringent and the inconsistencies of linkage distances between male and female heterozygous markers were resolved. Therefore, we excluded the previous Fig S20, S21, and presented the maps based on all markers (SNP + PA markers) for evaluation of the recombination rate (current Fig S9, S10).

All subsequent analyses were reperformed using the new pseudo-chromosomes based on the high-quality genetic maps.

The new reference genomes were uploaded in zenodo: https://doi.org/10.5281/zenodo.14899024.

13. I strongly recommend including the distribution of sex-linked SNPs along chromosome 3. The authors should map the density of sex-associated SNPs and/or male-heterozygous+female-homozygous (presumably XY) SNPs along this chromosome. Applying published methods for such analyses (e.g., refs. 1, 2, or RAD-seq–specific approaches such as ref. 3) would be highly beneficial. Cross-comparison of these results with high-quality male and female recombination data would help delineate the SDR and PAR boundaries, thereby greatly strengthening all downstream analyses.

References

1. <https://gitlab.in2p3.fr/sex-det-family/sex-detector-plusplus>

2. Käfer J, Lartillot N, Marais GAB, Picard F. Detecting sex-linked genes using genotyped individuals sampled in natural populations. Genetics. 2021 Jun 24;218(2):iyab053. doi: 10.1093/genetics/iyab053. PMID: 33764439; PMCID: PMC8225351.

3. Wilson CA, High SK, McCluskey BM, Amores A, Yan YL, Titus TA, Anderson JL, Batzel P, Carvan MJ 3rd, Schartl M, Postlethwait JH. Wild sex in zebrafish: loss of the natural sex determinant in domesticated strains. Genetics. 2014 Nov;198(3):1291-308. doi: 10.1534/genetics.114.169284. Epub 2014 Sep 18. PMID: 25233988; PMCID: PMC4224167.

Thank you for the valuable comments to improve the manuscript. To show the distribution of sex-associated SNPs along the sex chromosomes, we added two analyses; SDpop (Käfer et al., 2021, suggested as ref. 2) and RADSex (Felon et al., 2021, an optimized approach of ref. 3).

SDpop analysis was conducted using newly obtained Illumina short read sequences of five females and five males collected from a natural population. SDpop’s inferences of sex-linkage clearly indicated the SDR and PAR boundaries in Y chromosome (Fig S20A in the revised version). No clear SDR and PAR boundaries were detected in X chromosome by SDpop analysis (Fig S20B).

RADSex analysis was performed using RAD-seq data of 127 flowering F_1_ progeny segregating to male and female, showing the RAD markers associated with sex in the middle of chromosome 3 (Fig S21 in the revised version).

We explained these new results in the main text L220-L241. Based on the results of our association analysis, mapping coverage analysis, and the new SDR detection analyses, we redefined the putative X- and Y-specific SDRs spanning ~3.4 Mb and ~4.5 Mb, respectively. All subsequent analyses were reperformed based on the putative X- and Y-specific SDRs. Finally, we thank you again for the suggestions to improve the paper.
