# Supplementary material for: Whole-genome sequencing reveals a possible molecular basis of sex determination in the dioecious wild yam Dioscorea tokoro
Source: PLoS Genet. 2026 Apr 20;22(4):e1012123. doi: 10.1371/journal.pgen.1012123 (PMC13128126; doi:10.1371/journal.pgen.1012123)
Supplement: Attachment — Submitted filename: Response to reviewers_3rd revise_v9.0_rt_ak_rt_form.docx [file pgen.1012123.s045.docx]

**Response to reviewers**

Dear Editor,

Thank you very much for taking care of our manuscript PGENETICS-D-25-00572R2. We are pleased to submit the fully revised version taking valuable comments from you and the reviewer #2. In the following replies to the review, we re-numbered the comments. Original comments are in black, and our replies are in blue letters. The line numbers in our replies correspond to those in the current (revised) version of the manuscript.

**Reply to the Editor:**

**Additional Editor Comments:**

1. I was pleased to see that this manuscript has been carefully revised, and the Referee also found the revisions overall satisfactory, they only had a few additional suggestions left, in particular on the SDpop analysis and the dS analyses. I also have additional suggestions below.

Please revise carefully all figure legends: all abbreviations should be defined in all legends, all information should be given on what is seen on the figures in all legends, and edit them for clarity. All figures should be understandable on their own without reading the text or the other figures.

Thank you very much for your comments to improve the manuscript. To address the problems caused by lower quality genome assembly of Waka1 female individual (XX genotype), we (A) newly conducted Oxford Nanopore sequencing of the Waka1 individual using R10.4.1 flow cells and totally updated the female reference genome containing X-chromosome (Waka1 haplotype 1 assembly), which attained a high quality similar to the Kita1 (male) haplotype 1 assembly. All the analyses including SDpop were newly performed using the Waka1 haplotype 1 assembly (with X-chromosome) and the Kita1 haplotype 1 assembly (with Y-chromosome). In addition, following the suggestion of reviewer 2, we (B) updated the *d_S_* analysis. Details are given below. We revised all figure legends to ensure that they include all the necessary information. We also fully revised the manuscript in accordance with your comments as well as the reviewer's comments.

**(A) A new female reference genome using R10.4.1 flow cells**

To improve sequence quality of the female reference genome, we re-sequenced the female individual (Waka1) using R10.4.1 flow cell. We obtained a new female genome assembly of 425.1 Mb containing 212 contigs (two telomere-to-telomere contigs) for haplotype 1, and 342.2 Mb containing 346 contigs (no telomere-to-telomere contig) for haplotype 2. After anchoring the contigs, we obtained the X-chromosome containing only three contigs. For both the newly assembled X-chromosome and the previously assembled Y-chromosome (consisting of only two contigs), the putative SDRs were located in the middle of contigs (Fig. 2D, Fig SM3, SM4), suggesting that we are able to address their structural divergence with confidence. We performed all the analyses using this improved female reference (Waka1 haplotype 1 with X-chromosome) as well as the previously obtained male reference (Kita1 haplotype 1 with Y-chromosome). Accordingly, we updated the composition of Fig 2, Fig. 3, and Fig. S22. We revised Fig S17 with simple interval mapping plots.

Based on the newly constructed female assembly, we conducted the SDpop analysis of chr3-X. The SDpop analysis using 5 females and 5 males showed clear SDR boundaries on chr3-Y. However, SDR boundaries on chr3-X were not as clear as those on chr3-Y (Fig S23 in revised version). Since chr3-Y and chr3-X reference sequences have comparable qualities, we think the difference in SDpop results is not due to the quality difference of the reference genomes. At the moment we have no clear answer why such difference was observed. We will address the reason in future study.

**(B) Updates on the *d_S_* analysis**

Following the reviewer’s comment, we revised Fig S25 and Result to report the median of *d_S_* values instead of the average. We removed the gametolog showing *d_S_* value > 1 because they could not be unambiguously classified as gametologs. We revised Fig S26 to show only the homologous genes located on X and Y regions along the sex chromosomes and highlighted the *d_S_* values of the XY gametologs.

2. -Figure S23: the legend is highly unclear and incomplete. For example: dS should be defined, dS had been computed between X and Y gametologs right? This is unclear from the figure legend, it reads as if it was between X or between Y alleles? The red and blue traits are not defined.

Thank you for the comment. We revised the legend to include the definition of *d_S_* and explanations of the red and blue traits in Fig S26 (revised version). We tried our best to make the legend explanation as clear and complete as possible.

3. -L256-259: unclear and awkward. Remove “especially”, as it has no relationship with the previous sentence. Delete also “and thought to be involved in sex chromosome evolution”, as it sounds vague and unclear. In addition, it is a typical feature of non-recombining regions, not only plant sex chromosomes.

Thank you for the comment. We revised the sentence in L 288-L290, “Accumulation of repetitive DNA in the non-recombining regions (NRY) surrounding the SDR are reported as a conspicuous feature of sex chromosomes [68–72].”

4. -L292, L816 and elsewhere: synonymous, not silent

Thank you for the comment. We revised it in L315 and L833.

5. -L270-271: this is more like discussion, and with also discussion of sex-determining regions being located in pericentromeric regions in plants (e.g. in Silene latifolia and Rumex).

We moved the explanation to the Discussion, L479-L482. We added the previous reports of *Silene latifolia* and *Rumex hastatulus* in L 484-485.

6. -L293: “to infer the evolutionary context”: this is too vague and unclear.

We revised it in L317-L318, “to test whether the X–Y differentiation is relatively ancient or recent compared with species divergence within the genus.”

7. -L294-298: this is unclear, explain what are the hypotheses tested, and even in the introduction already

Thank you for the comment. We added the hypothesis and explanation in L107-L119, “It is therefore important to clarify the evolutionary history of sex determination system in the genus. Which species maintain the ancestral state of the sex determination of the genus? How and when did the transitions happen from the ancestral state? Once we identify the SDRs, we may be able to identify the homologous genes located on the X- and Y-specific regions (XY gametologs) [45,46] and estimate their divergence by calculating their synonymous substitution rate to infer the evolutionary history of SDRs of the species. Also, in recent studies, stepwise sequence divergence patterns in the SDRs referred to as the evolutionary strata have been reported as the consequence of serial recombination suppression in the SDRs in animal and fungal sex chromosomes [47–50], as well as in plant sex chromosomes of *Silene latifolia* and its relative species [51–54] and hops [22]. Elucidating the trajectory of sex chromosome evolution and evolutionary strata of SDRs in the genus *Dioscorea* will also advance our understanding of the evolution of sex-determination systems.”

8. -L312-315: this is also the case in many animal and fungal systems, and should be introduced in introduction rather than in the result section

We cited the cases in animal and fungal systems and moved the sentence to the Introduction L113-L117, “Also, in recent studies, stepwise sequence divergence patterns in the SDRs referred to as the evolutionary strata have been reported as the consequence of serial recombination suppression in the SDRs in animal and fungal sex chromosomes [47–50], as well as in plant sex chromosomes of *Silene latifolia* and its relative species [51–54] and hops [22].”

9. -L318-319 and elsewhere: the lack of evolutionary does not mean that divergence is recent, but that there has been no successive extension with time. It is the value of the dS in the sex-determining region that indicates that it is recent. All this needs to be explained in the introduction.

We revised it in the Results L341-344, “The distribution of *d_S_* values of X–Y gene pairs in *D. tokoro* showed no clear evolutionally strata throughout the X and Y chromosomes (Fig S26); we could not detect any signature of successive extension of recombination suppression following the X–Y differentiation in *D. tokoro*.”

As explained above, we added the explanation about evolutionary strata in the Introduction L113-119, “Also, in recent studies, stepwise sequence divergence patterns in the SDRs referred to as the evolutionary strata have been reported as the consequence of serial recombination suppression in the SDRs in animal and fungal sex chromosomes [47–50], as well as in plant sex chromosomes of *Silene latifolia* and its relative species [51–54] and hops [22]. Elucidating the trajectory of sex chromosome evolution and evolutionary strata of SDRs in the genus *Dioscorea* will also advance our understanding of the evolution of sex-determination systems.”

10. -L459 and elsewhere: do not refer to figures in the discussion

We removed all referring of figures in the discussion.

11. -L480: the dS analysis (S in subscript across the whole manuscript)

Thank you for the comment. We revised it in the whole manuscript.

12. -L482: comma after that

We added a comma in L501. Thank you very much for your suggestions to improve the paper.

**Reply to the REVIEWER #2:**

13. I thank the authors for their efforts in addressing my previous comments.

I still have two comments:

Thank you very much for your valuable comments to improve the manuscript. We fully revised the manuscript by incorporating your two suggestions.

14. The SDpop analysis of chr3-Y nicely shows the SDR boundaries (Fig. S20). I was expecting to observe a similar pattern for chr3-X, which is not the case. As far as I understand, separate mappings were performed against the haploid female reference genome and against the male reference genome (haplotype 1 only). It appears that mapping to the female genome may be less efficient; providing the percentage of mapped reads for both references would help clarify this. If BWA parameters were identical for both mappings, the discrepancy may stem from differences in sequence quality, as the female genome was generated using R9.4.1 flow cells, which have a higher error rate. Could the authors indicate the Phred quality scores for both assemblies?

Thank you for the comment. To solve the problems caused by the lower sequence quality of the female reference genome, we newly performed Oxford Nanopore sequencing of the female individual Waka1 (with XX genotype) using R10.4.1 flow cells and obtained the haplotype 1 and 2 assemblies (Table S1). We believe the quality of the newly obtained Waka1 haplotype 1 (Table S1) is sufficient to infer the structure of X-chromosome. Accordingly, we newly performed all the analyses including SDpop using Waka1 haplotype 1 assembly (with X-chromosome) and the previously obtained Kita1 haplotype 1 assembly (with Y-chromosome).

Specifically, resequencing of the Waka1 female individual using R10.4.1 flow cell largely improved the amount of the sequence data and the Phred quality scores (see Table A below). Based on the new sequence data, we obtained a new female genome assembly of 425.1 Mb containing 212 contigs (two telomere-to-telomere contigs) for haplotype 1, and 342.2 Mb containing 346 contigs (no telomere-to-telomere contig) for haplotype 2. The new pseudo-chromosomes for female were reconstructed based on the high-quality genetic maps. After construction of the pseudo-chromosomes, we obtained X-chromosome containing only three contigs. For both the newly assembled X-chromosome and the previously assembled Y-chromosome (consisting of only two contigs), the putative SDRs were located in the middle of contigs (Fig 2D, SM3, SM4). All the subsequent analyses were performed again using this new reference sequence.

**Table A**

| Feature | Kita1 (male) | Waka1 (female)  Previous version | Waka1 (female)  New version |
| --- | --- | --- | --- |
| Flow cell | R10.4.1 | R9.4.1 | R10.4.1 |
| Number of reads | 3,890,192 | 668,621 | 2,175,454 |
| Total base pairs (Gbp) | 42.6 | 15.9 | 48.5 |
| Genome coverage | 109.8x | 41.1x | 125.1x |
| Q20 (% of bases with Phred score > 20) | 81.7 | 53.3 | 91.8 |
| Q30 (% of bases with Phred score > 30) | 70.1 | 16.2 | 85.3 |

After construction of the high-quality female reference genome, we conducted SDpop analysis again. Mapping rates of individuals were sufficiently high (Table B). SDpop analysis using 5 females and 5 males showed clear SDR boundaries on chr3-Y. However, SDR boundaries on chr3-X were not as clear as those on chr3-Y (Fig S23 in revised version). Since chr3-Y and chr3-X reference sequences have comparable qualities, we think the difference in SDpop results is not due to the quality difference of the reference genomes. At the moment we have no clear answer why such difference was observed. We will address the reason in future study.

**Table B**

|  | Male haplotype 1 | Female haplotype 1  (newly constructed) |
| --- | --- | --- |
| 5 females | 98.5% | 98.7% |
|  | 98.1% | 98.3% |
|  | 99.4% | 99.6% |
|  | 98.5% | 98.8% |
|  | 98.9% | 99.2% |
| 5 males | 98.5% | 98.7% |
|  | 98.5% | 98.6% |
|  | 98.8% | 98.9% |
|  | 99.2% | 99.4% |
|  | 99.3% | 99.6% |

15. Figure S22 clearly shows that the SDR is younger than the divergence between D. tokoro and its relatives. However, given that the dS distributions are non-normal, I would recommend reporting median rather than mean values. The gametologs showing dS values > 1 are surprising — do they belong to multigene families? Could these be paralogs rather than true X/Y gametologs?

In Figure S23, I suggest displaying dS values only for the gametologs. For PAR genes, only allelic differences are expected. Showing dS for all homologous genes, including paralogous ones is confusing.

Thank you very much for your valuable comments. We revised Fig S25 (Fig S22 in the previous version) and Results to report the median *d_S_* value instead of the average value. We removed the gametolog showing *d_S_* value > 1 because they could not be unambiguously classified as gametologs.

We revised Fig S26 (Fig S23 in the previous version) to show *d_S_* of only the homologous genes of the X and Y chromosomes and highlighted the *d_S_* values of the XY gametologs.

Again, thank you very much for your comments to improve the paper.
