# Supplementary material for: Whole-genome sequencing reveals a possible molecular basis of sex determination in the dioecious wild yam Dioscorea tokoro
Source: PLoS Genet. 2026 Apr 20;22(4):e1012123. doi: 10.1371/journal.pgen.1012123 (PMC13128126; doi:10.1371/journal.pgen.1012123)
Supplement: Attachment — Submitted filename: Response to reviewers_4th revise_v3.0.docx [file pgen.1012123.s046.docx]

**Response to reviewers**

Dear Editor,

Thank you very much for taking care of our manuscript PGENETICS-D-25-00572R3. We are pleased to submit the fully revised version taking valuable comments from you and the reviewer #2. In the following replies to the review, we re-numbered the comments. Original comments are in black, and our replies are in blue letters. The line numbers in our replies correspond to those in the current (revised) version of the manuscript.

**Reply to the Editor:**

**Additional Editor Comments:**

1. This manuscript has been further improved, but the Referee still had important concerns about the SDpop analysis, with a clear pattern when using the Y chromosome as a reference but not when using the X chromosome, and with the dS analysis, with higher values in the pseudo-autosomal region than in the non-recombining region. There may be paralogs included and it may be helpful to seek expert advice for resolving these issues.

Thank you very much for your comments on the manuscript. We obtained an expert advice from Dr. Hideki Innan of The Graduate University for Advanced Studies, Japan, a prominent population geneticist. Dr. Innan provided helpful suggestions to solve the problems and is now included as a co-author of the revised version of the paper. Following his suggestions, we reanalyzed SDpop analysis and *d_S_* analysis.

In the SDpop analysis (Fig S23), we used only the polymorphic sites located within the coding regions of orthologous gene pairs of chromosome 3Y (from Kita1 haplotype 1) and chromosome 3X (from Waka1 haplotype 1). As a results, high posterior probabilities of XY gametolog segregation type and low posterior probabilities of autosomal segregation type were detected in the middle of chromosome 3 for both the Waka1 (female) haplotype 1 assembly and the Kita1 (male) haplotype 1 assembly.

In the *d_S_* analysis (Fig S26), we applied a threshold of *d_S_* < 0.5 to filter out potential paralogous X–Y gene pairs with excessively large *d_S_* values, based on the maximum *d_S_* values in the SDRs reported in the previous studies (Akagi *et al.*, 2025; Moraga *et al.*, 2025). As a result, the distribution of *d_S_* values of X–Y gene pairs showed almost zero throughout the X and Y chromosomes. We obtained similar results with a less stringent threshold of *d_S_* < 1.0.

**Reply to the REVIEWER #2:**

2. I thank the authors again for their efforts in addressing my comments. In particular, the sequencing and assembly of the female genome represent a very important addition to this work. Unfortunately, this does not resolve the puzzling pattern obtained with SDpop when using the female genome as a reference.

Thank you very much for your comments to improve the manuscript. Following the editor’s suggestion, we sought an advise from Dr. Hideki Innan, a prominent population geneticist to improve the paper. Accordingly, in this revised version, we used only the polymorphic sites located within the coding regions of the orthologous gene pairs of chromosome 3Y (from Kita1 haplotype 1) and chromosome 3X (from Waka1 haplotype 1). As a results, high posterior probabilities of XY gametolog segregation type and low posterior probabilities of autosomal segregation type were detected in the middle of chromosome 3 for both the Waka1 (female) haplotype 1 assembly and the Kita1 (male) haplotype 1 assembly (Fig S23). SDpop analysis using only SNPs on the genic regions has been reported in *Silene latifolia* (Käfer et al., 2021) and has an effect to remove the noises potentially caused by using SNPs in non-orthologous positions.

Käfer, J. et al. (2021). Detecting sex-linked genes using genotyped individuals sampled in natural populations. *Genetics*, *218*(2), iyab053. <https://doi.org/10.1093/genetics/iyab053>

3. Figure S25 is much improved, but Figure S26, which shows dS values along the 3X and 3Y chromosomes, remains difficult to interpret. dS reaches very high values, much higher than expected for two alleles in a pseudoautosomal regions (PARs). In addition, dS values in the SDR seem actually somewhat lower than those in the PARs, which is again unexpected. Moreover, the sliding-window analysis shows markedly different patterns for the SDR-3X and SDR-3Y, with a peak present only in SDR-3Y, which I find difficult to understand. This figure could likely be further improved.

Thank you for the comment. We applied a threshold of *d_S_* < 0.5 to filter out potential paralogous X–Y gene pairs with excessively large *d_S_* values, based on the maximum *d_S_* values in the SDRs reported in the previous studies (Akagi *et al.*, 2025; Moraga *et al.*, 2025). As a result, the distribution of *d_S_* values of X–Y gene pairs showed almost zero throughout the X and Y chromosomes. We obtained similar results with a less stringent threshold of *d_S_* < 1.0 (Fig S26).

Akagi, T. et al. Evolution and functioning of an X–A balance sex-determining system in hops. Nat. Plants 11, 1339–1352 (2025). <https://www.nature.com/articles/s41477-025-02017-6>

Moraga, C. et al. The Silene latifolia genome and its giant Y chromosome. Science387,630-636 (2025). <https://www.science.org/doi/abs/10.1126/science.adj7430>
