# Supplementary material for: Whole-genome sequencing reveals a possible molecular basis of sex determination in the dioecious wild yam Dioscorea tokoro
Source: PLoS Genet. 2026 Apr 20;22(4):e1012123. doi: 10.1371/journal.pgen.1012123 (PMC13128126; doi:10.1371/journal.pgen.1012123)
Supplement: Attachment — Submitted filename: Response to reviewers_260403_ak.docx [file pgen.1012123.s047.docx]

**Response to reviewers**

Dear Editor,

Thank you very much for taking care of our manuscript PGENETICS-D-25-00572R4. We are pleased to submit the fully revised version taking into account the comments from you and the reviewer, as detailed below. We re-numbered the comments. Original comments are in black, and our replies are in blue letters. The line numbers in our replies correspond to those in the current (revised) version of the manuscript.

**Editor Comments:**

1. I was pleased to see that this manuscript has been carefully revised. The referee still has some minor suggestions, but other than that, I congratulate the authors for this nice piece of work that will be a great contribution to the journal.

Thank you very much for your comments on the manuscript. We revised the manuscript following the suggestions of the reviewer.

**Reviewer comments:**

2. The manuscript has significantly improved since I last read it after its first submission. I have some last comments to further improve it before publication.

We are grateful for these final comments from the reviewer, which have indeed improved the manuscript.

Regarding the dS analysis: please state well in the main text that you filtered dS values to be lower than 0.5.

We add the explanation in L339-341, “We applied a threshold of *d_S_* < 0.5 to filter out potential paralogous X–Y gene pairs with excessively large *d_S_* values, based on the maximum *d_S_* values observed in SDRs in previous studies [22,51]. We also evaluated it with a less stringent threshold of *d_S_* < 1.0.”.

3. In Figure 2F we can see some female reads mapping on the male haplotype 1, unlike stated in the text lines 237-238 that coverage is zero in females.

We revised L236-239, “When we mapped the Illumina short reads to the Kita1 (male) haplotype 1 assembly, we identified a region in the middle of chromosome 3 that was enriched for sites covered by male reads at 0.5× depth and female reads at 0× depth (Fig 2F)”.

4. Line 258-260: the SDpop results suggest that both X and Y reads are able to map on male haplotype 1 and female haplotype 1, because X/Y SNPs are found. This result suggests that X and Y sequences are poorly diverged (which is consistent with the dS analysis).

However, in Figure 2E-F you show that mapping is 0.5 for male illumina reads on male haplotype 1. Which is contradictory with the SDpop analysis.

It is contradictory to have at the same time half coverage on the X in males and X/Y SNPs. It is usually one or the other. These correspond to different stages of sex chromosome evolution. Please refer to reviews on identifying sex chromosomes such as Palmer et al 2019 or Muyle et al 2017.

Thank you for the comment. We hypothesize that the observed ~0.5x coverage of male reads on the X chromosome, together with the presence of X/Y SNPs, is caused by a mixture of highly diverged and less diverged regions within the SDRs of X- and Y-chromosomes. This interpretation is supported by three observations: (1) direct sequence comparison of SDR regions of X- and Y-chromosomes suggest a mixture of conserved and divergent regions (Fig 2DG). (2) a region in the middle of chromosome 3 of X chromosome is enriched for sites covered by male reads at 0.5× depth and female reads at 1× depth, but this pattern is not contiguous across the region (a similar tendency was also observed on the Y chromosome), and (3) the X- and Y- regions contain both X- or Y- specific genes and XY gametologs.

We added these observations by revising L232-L239, “We identified a region in the middle of chromosome 3 that was enriched for sites covered by male reads at 0.5× depth and female reads at 1× depth in the Waka1 (female) haplotype 1 assembly (Fig 2E). Such consistent depth differences between male and female reads were not observed in other chromosomes (Fig. S18). When we mapped the Illumina short reads to the Kita1 (male) haplotype 1 assembly, we identified a region in the middle of chromosome 3 that was enriched for sites covered by male reads at 0.5× depth and female reads at 0× depth (Fig 2F)”. We also added Fig 2G and L277-280, “These candidate genes include X- or Y-specific genes and putative orthologous genes (gametologs; [45,46]). A total of 22 genes in the Y- region had corresponding genes in the X-region so that they were defined as XY gametologs (Fig 2G)”.

We referred to Palmer et al 2019 and Muyle et al 2017 in L228.

5. The only explanation I see is maybe how mapping was done. Did you map illumina reads on haplotypes one by one for coverage analysis and SDpop? Or did you combine all haplotypes into a single reference and map illumina reads on this combined reference for the coverage analysis? For SDpop, the only way to obtain X/Y SNPs was to map reads on a single haplotype. The procedure should be clarified in the Results and Methods.

We aligned the Illumina short reads on haplotypes one by one for the coverage analysis and SDpop.

We clarified this in Results L228-230, “To assess whether this is the case for *D. tokoro*, we aligned Illumina short reads obtained from female and male individuals to each haplotype of chromosome 3 and studied the mapping depth”, and L254-257, “Based on the alignments of these sequencies to each haplotype reference, empirical Bayes posterior probabilities in the SDpop model inferred three segregation types at each polymorphic site: autosomal segregation type, X-hemizygosity segregation type and XY gametology segregation type”.

We also added it in Methods L797-798, “The alignment to each haplotype reference was conducted using BWA v0.7.17-r1188 [129]” , and L810-811, “The alignment to each haplotype was conducted using BWA v0.7.17-r1188 [129]”.

6. Figure 2D should have another version zoomed on the X and Y specific region. This would be a Figure similar to Figure S25C, with ribbons connecting X/Y gametologs, please make it a main Figure, but zoom on the X/Y specific region and comment it in the main text. This Figure highlights rearrangements between the X and the Y and is important.

We added the zoomed-in image of SDR as an inset of Fig 2D and also provided Fig 2G with ribbons connecting X/Y gametologs, and described it in L275-280, “These chromosomes are differentiated into putative X- and Y-specific regions spanning ~4.8 Mb with 54 candidate genes (Table S3) and ~4.5 Mb with 56 candidate genes (Table 1), respectively. These candidate genes include X- or Y-specific genes and putative orthologous genes (gametologs; [45,46]). A total of 22 genes in the Y- region had corresponding genes in the X-region so that they were defined as XY gametologs (Fig 2G).”

7. Lines 320-326 describing gene orthology should appear before the dS description line 313.

We moved the sentence before L323, where detailed of *d_S_* comparison was described. As L315 provides an overview of this analysis, we consider it more appropriate to place the sentence before L323.

8. I believe Figure S27 should be a main Figure. It is a lot more interesting than Figure 3 for example.
   The result showing synteny between D tokoro and alata should be discussed further. In Figure S27B it is not possible to say if the tokoro SDR is syntenic to the alata SDR, a Figure with a zoom would be needed. If the two SDRs are syntenic, it would be interesting to consider if they evolved in the ancestor of the two species, or if they result from convergent evolution. The dS analysis comes in handy to suggest it is convergent evolution.

We moved Fig S27 to Main Fig 4. For the zoomed comparison between *D. tokoro* and *D. alata*, although the most recent preprint (Dossa et al., 2025) reported SDR position in *D. alata*, the updated gene annotation data is not publicly available. Therefore, our capacity is currently limited in conducting a detailed gene-order-based synteny analysis to assess the correspondence of SDRs in the two species.

We revised the discussion L500-505 to describe the possibility of convergent evolution, “The genomic region around the SDR of *D. tokoro* is also syntenic with the sex chromosome of *D. alata* in our synteny analysis. However, *d_S_* analysis suggested that the divergence between *D. tokoro* Y- and X-linked genes is more recent than the divergence between *D. tokoro* and *D. alata*. These findings suggest a possibility of convergent evolution in which the same genomic regions came to be involved in sex determination independently in *D. tokoro* and *D. alata*.”

Komivi Dossa, Xiaojun Su, Yedomon Ange Bovys Zoclanclounon et al. Haplotype-resolved genome assembly of a male Dioscorea alata cultivar reveals the structure and evolution of sex chromosomes, 09 September 2025, PREPRINT (Version 2) available at Research Square [https://doi.org/10.21203/rs.3.rs-5627324/v2]

9. Could you add the BLH9 and HSP90 gene names in Table 1 please to identify more easily the lines corresponding to these candidates.

We added the gene names in Table 1.

10. Line 501 what do you mean by "the genes involved in sex determination may have diverged"?

I rather think the conclusion is that the same region became sex-linked convergently in the two species.

We revised the sentence in L503-505, “These findings suggest a possibility of convergent evolution in which the same genomic regions came to be involved in sex determination independently in *D. tokoro* and *D. alata*”.
